# Total synthesis and antimicrobial evaluation of natural albomycins against clinical pathogens

**DOI:** 10.1038/s41467-018-05821-1

**Published:** 2018-09-04

**Authors:** Zihua Lin, Xiaobo Xu, Sheng Zhao, Xiaohong Yang, Jian Guo, Qun Zhang, Chunmei Jing, Shawn Chen, Yun He

**Affiliations:** 1 Chongqing Key Laboratory of Natural Product Synthesis and Drug Research, School of Pharmaceutical Sciences, Chongqing University, 55 Daxuecheng South Road, 401331 Shapingba, Chongqing China; 2 College of Chemistry and Pharmaceutical Engineering, Huanghuai University, 463000 Zhumadian, China; 3 Medicine Laboratory, Ministry of Education Key Laboratory of Child Development and Disorders, Children’s Hospital of Chongqing Medical University, 136 Zhongshan 2nd Rd, 400014 Yuzhong, Chongqing China; 4 Global Health Drug Discovery Institute, School of Pharmaceutical Sciences, Tsinghua University, 30 Shuangqing Rd, 100084 Haidian, Beijing China

## Abstract

Development of effective antimicrobial agents continues to be a great challenge, particularly due to the increasing resistance of superbugs and frequent hospital breakouts. There is an urgent need for more potent and safer antibiotics with novel scaffolds. As historically many commercial drugs were derived from natural products, discovery of antimicrobial agents from complex natural product structures still holds a great promise. Herein, we report the total synthesis of natural albomycins *δ*_1_ (**1a**), *δ*_2_ (**1b**), and *ε* (**1c**), which validates the structures of these peptidylnucleoside compounds and allows for synthetic access to bioactive albomycin analogs. The efficient synthesis of albomycins enables extensive evaluations of these natural products against model bacteria and clinical pathogens. Albomycin *δ*_2_ has the potential to be developed into an antibacterial drug to treat *Streptococcus pneumoniae* and *Staphylococcus aureus* infections.

## Abstract (web summary)

Albomycins are promising drug candidates for the treatment of bacterial infections. Here, the authors describe the total syntheses of albomycins *δ*_1_, *δ*_2_, and *ε*, and evaluate their antimicrobial activity, identifying albomycin *δ*_2_ as a strong agent against *S. pneumoniae* and *S. aureus* infections.

## Introduction

Sideromycins are a class of antibiotics covalently linked to siderophores^1^. They are actively transported into bacterial cells via siderophore uptake pathways commonly found in bacterial pathogens, by the so-called “Trojan horse” strategy, resulting in outstanding cell envelope permeability and very low minimum inhibitory concentrations (MICs). These pathogen-specific antibiotics are promising drug candidates for the treatment of various bacterial infections^2–4^. A few naturally occurring sideromycins have been discovered. Among these, albomycins, originally reported as grisein, were first isolated from soil microorganism *Streptomyces griseus* in 1947^5–9^. Albomycins exhibited potent inhibitory activities against a number of Gram-negative, as well as Gram-positive bacteria, including multi-drug resistant strains^1,10,11^. For instance, albomycins exhibited an MIC value of 10 ng/mL against *Streptococcus pneumoniae* and 5 ng/mL against *Escherichia coli*, which is almost tenfold more potent than penicillin^12^. Moreover, no toxicity was observed during in vivo studies of albomycins, and it was well tolerated and safe up to a maximum dose evaluated in mice^10^. Albomycins have been successfully used to treat human bacterial infections in the Soviet Union^10^.

**Fig. 1:**
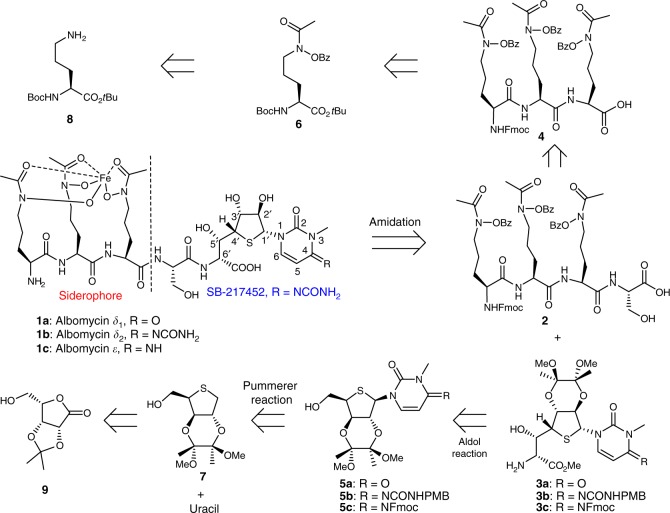
Chemical structures of albomycins *δ*_1_, *δ*_2_, and *ε*, and their synthetic strategies. The key disconnections involve a Pummerer reaction for nucleobase introduction and an aldol reaction to expand the side chain of thionucleoside. *tBu* *tert*-butyl, *Bz* benzoyl, *Fmoc* fluorenylmethyloxycarbonyl, *PMB* *p*-methoxybenzyl

The structures of albomycins were fully elucidated by Benz and coworkers in 1982^13,14^, 35 years after their initial isolation. Albomycins *δ*_1_ (**1a**), *δ*_2_ (**1b**), and *ε* (**1c**) are all composed of a tri-δ-*N*-hydroxy-L-ornithine peptide siderophore and a thionucleoside warhead with six consecutive chiral centers, and differ only in the C4 substituent (R) of the pyrimidine nucleobase (Fig. 1). As for **1b**, the thionucleoside warhead is a potent seryl-tRNA synthetase inhibitor known as SB-217452^15^. The highly complex and densely functionalized structures, together with their important therapeutic potential, have made albomycins attractive targets for synthesis. Different synthetic strategies for the tri-δ-*N*-hydroxy-L-ornithine peptide siderophore have been described by the groups of Benz^16,17^ and Miller^18–20^. The synthesis of the thionucleoside moiety of **1a** was briefly described by Holzapfel et al. in 1991^21^, though with incomplete data. However, no total synthesis of albomycins has been reported. In one study^22^, an oxygen analog of **1a** was synthesized. Surprisingly, a single replacement of the sulfur with oxygen resulted in the complete loss of antibacterial activity, suggesting a critical role of the sulfur atom in the activity of albomycins. A biosynthetic approach has also been attempted for the generation of albomycin analogs^23^, but albomycin production by *S. griseus* is difficult and has yet to be scaled up efficiently^24^. Herein, we describe the total synthesis of the three natural albomycins (**1a**–**c**), which features a Pummerer reaction for nucleobase introduction and an aldol reaction to expand the side chain of thionucleoside. Their biological evaluations demonstrate that albomycin *δ*_2_ is a promising lead candidate for treating *S. pneumoniae* and *S*. *aureus* infections and warrants further development.

## Results

### Synthetic strategy

The retrosynthetic analysis of albomycins (**1a**–**c**) in a collective fashion is shown in Fig. 1. The amide bond linking to the thionucleoside core was first disconnected to generate tetrapeptide fragment **2** and thionucleosides **3a**–**c**. Tetrapeptide **2** could be readily accessed via the condensations of tripeptide **4** with L-serine *tert*-butyl ester. Tripeptide **4** could be derived from amino acid **6**, which could arise from the direct oxidation of protected L-ornithine **8**. We anticipated that thionucleosides **3a**–**c** could be installed by substrate-directed asymmetric aldol condensation reaction from thionucleosides **5a**-**c**, which could in turn be prepared through a Pummerer reaction from thiosugar **7** and uracil. Thiosugar **7** could be further traced back to the known L-(+)-lyxose derivative **9**. Due to the structural feature and labile nature of albomycins, the protecting group strategy had to be delicately selected to accomplish the total synthesis. Because of the presence of reducible hydroxamic acids in the siderophore moiety and an imine group in the thionucleoside moiety of **1b** and **1c**, protecting groups such as Cbz and Bn which usually need to be deprotected under reducing conditions of H_2_ and Pd/C in the final stage were avoided. As for the hydroxyl groups in the thionucleoside fragment, orthogonality of protecting groups and their influence on stereoselectivity of uracilation were also key considerations.

### Total synthesis of albomycins *δ*_1_, *δ*_2_, and *ε*

**Fig. 2:**
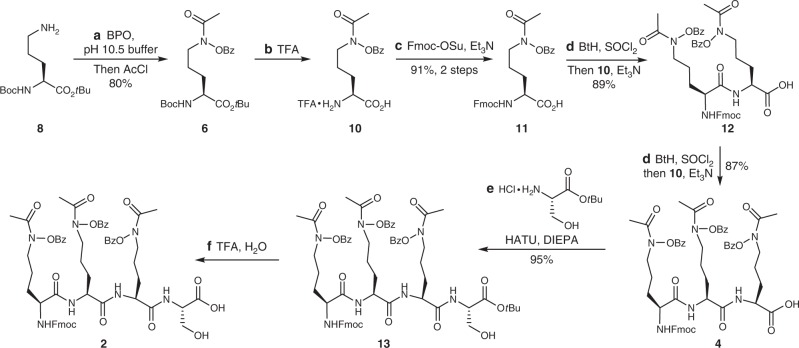
Synthesis of tetrapeptide **2**. Reagents and conditions: **a** BPO (2.0 equiv), pH 10.5 buffer, CH_2_Cl_2_, 3 h, then AcCl (1.2 equiv), CH_2_Cl_2_, 80%; **b** TFA, H_2_O, 12 h; **c** Fmoc-OSu (1.2 equiv), Et_3_N (3.0 equiv), DMF, −15 °C, overnight, 91% (2 steps); **d** BtH (4.0 equiv), SOCl_2_ (1.0 equiv), THF, 50 min, then **10** (1.1 equiv), Et_3_N (3.1 equiv), CH_3_CN/H_2_O (5:2), −15 °C, 2 h, **12**: 89%, **4**: 87%; **e** H-Ser(*t*Bu)-O*t*Bu hydrochloride (1.1 equiv), HATU (1.5 equiv), DIPEA (2.0 equiv), DMF, −15 °C, 2 h, 95%; **f** TFA, H_2_O, CH_2_Cl_2_, 0 °C, 18 h. *BPO* benzoyl peroxide, *AcCl* acetyl chloride, *TFA* trifluoroacetic acid, *Fmoc-OSu* *N*-(9-fluorenylmethoxycarbonyloxy)-succinimide, *DMF* *N*,*N*-dimethyformamide, *DCC* dicyclohexylcarbodiimide, *NHS* *N*-hydroxysuccinimide, *BtH* 1*H*-benzotriazole, *THF* tetrahydrofuran, *HATU* 1-[bis(dimethylamino) methylene]-1H-1,2,3-triazolo [4,5-b] pyridinium 3-oxid hexafluorophosphate, *DIPEA* *N*,*N*-diisopropylethylamine

Our synthetic efforts commenced with the preparation of tetrapeptide **2** (Fig. 2). The core component hydroxamic acid **6** of tetrapeptide **2** was first synthesized from *N*^2^-Boc-L-ornithine *tert*-butyl ester **8**^25^. Oxidation of the free amine in *N*^2^-Boc-L-ornithine *tert*-butyl ester **8** with benzoyl peroxide^25–27^, followed by acylation under biphasic conditions afforded the fully protected hydroxamate **6** in 80% yield. Removal of *tert*-butyl-carbonate (Boc) and *tert*-butyl (*t*-Bu) groups with trifluoroacetic acid (TFA) provided the TFA salt **10**. The resulting amine group was protected with Fmoc to deliver acid **11** in 91% yield over two steps. Then, we turned to coupling **11** and **10** under active ester-mediated coupling conditions (DCC, NHS)^20^, which proceeded smoothly to produce dipeptide **12**. Unfortunately, application of the same conditions to synthesize tripeptide **4** led to significant epimerization. Failure to optimize the condition after numerous efforts prompted us to explore an alternative strategy. Fortunately, under active amide-mediated coupling conditions developed by Katritzky^28–31^, tripeptide **4** could be obtained in an iterative fashion from **11** in 77% yield over two steps without any detectable epimerization. Tripeptide **4** was condensed with L-serine *tert*-butyl ester hydrochloride in the presence of HATU and DIPEA to generate *tert*-butyl ester **13** in 95% yield. Treatment of **13** with TFA in dichloromethane at 0 °C proceeded smoothly to yield the corresponding tetrapeptide **2**.

**Fig. 3:**
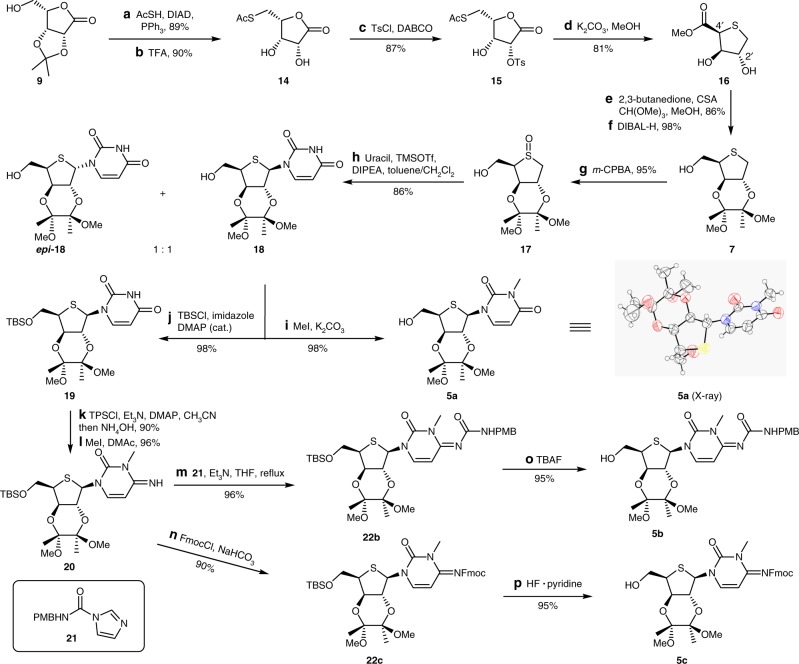
Synthesis of intermediates **5a**-**c** and X-ray crystal-structure diagram of **5a**. Reagents and conditions: **a** AcSH (2.2 equiv), DIAD (2.2 equiv), PPh_3_ (2.2 equiv), THF, 0 °C, 5 h, 89%; **b** TFA, CH_2_Cl_2_, H_2_O, 5 h, 90%; **c** TsCl (1.2 equiv), DABCO (1.1 equiv), CH_3_CN, 1 h, 87%; **d** K_2_CO_3_ (1.5 equiv), MeOH, −30 °C, 3 h, 81%; **e** 2,3-butanedione (1.2 equiv), trimethoxymethane (4.0 equiv), CSA (0.1 equiv), MeOH, reflux, 24 h, 86%; **f** DIBAL-H (2.1 equiv), CH_2_Cl_2_, 0 °C, 2 h, 98%; **g** *m*-CPBA (1.0 equiv), CH_2_Cl_2_, 0 °C, 0.5 h, 95%; **h** uracil (2.0 equiv), TMSOTf (8.0 equiv), DIPEA (8.0 equiv), toluene, CH_2_Cl_2_, 3 h, d.r. 1:1, 86%; **i** methyl iodide (1.2 equiv), K_2_CO_3_ (1.5 equiv), DMF, 2 h, 98%; **j** TBSCl (2.2 equiv), imidazole (2.2 equiv), DMAP (0.1 equiv), CH_3_CN, 2 h, 98%; **k** TPSCl (2.0 equiv), Et_3_N (2.0 equiv), DMAP (2.0 equiv), CH_3_CN, 1 h, then NH_4_OH, 20 h, 90%; **l** methyl iodide (3.0 equiv), DMAc, 3.5 h, 96%; **m** **21** (2.0 equiv), Et_3_N (1.0 equiv), THF, reflux, 96%. **n** Fmoc-Cl (2.0 equiv), NaHCO_3_ (4.0 equiv), THF/H_2_O, 90%; **o** TBAF (1.3 equiv), THF, 2 h, 95%; **p** HF·pyridine (5.0 equiv), THF, 36 h, 95%. *AcSH* thiolacetic acid, *DIAD* diisopropyl azodiformate, *DABCO* 1,4-diazabicyclo[2.2.2]octane, *CSA* camphorsulfonic acid, *DIBAL-H* diisobutylaluminum hydride, *TPSCl* 2,4,6-triisopropylbenzenesulfonyl chloride, *DMAP* 4-dimethylaminopyridine, *DMAc* *N*, *N*-dimethylacetamide, *TBAF* tetrabutylammonium fluoride

With tetrapeptide **2** in hand, we initiated the synthesis of thionucleosides **5a**-**c** (Fig. 3). At first, lactone **9**^32^ was transformed into lactone **14** by a two-step sequence involving Mitsunobu reaction and removal of the isopropylidene protecting group with TFA. Selective mono-tosylation of lactone **14** with tosyl chloride/DABCO generated tosylate **15** in 87% yield. In this case, use of pyridine led to low conversion because of its weaker basicity, whereas Et_3_N to a bis-tosylation byproduct which further underwent elimination to produce the corresponding *α,β*-unsaturated tosylate. The transformation of **15** to **16** with K_2_CO_3_ in MeOH at room temperature gave the product with the undesired configuration at C4' exclusively. A variety of bases and temperatures were examined for the 5-membered thio-ring closure reaction, and eventually the use of K_2_CO_3_ as a base at −30 °C in MeOH smoothly led to **16** in 81% yield without any detectable epimerization. It was surprising that the reaction temperature had such an impact on the stereoselectivity. As mentioned above, the proper choice of protecting group for the *trans*-1,2-diol is crucial, because the neighboring group effect could influence the stereoselectivity of the following uracilation reaction^33^. Initially, when the *trans*-1,2-diol was protected as a diester, the Pummerer reaction indeed provided the desired product as a single diastereomer with moderate yield. However, this protected diester was incompatible with the subsequent selective methyl ester reduction and later stage aldol reaction. Thus, *trans*-1,2-diol **15** was protected with 2,3-butanedione under acid catalysis, followed by reduction of the methyl ester with DIBAL-H to afford sulfide **7**. Treatment of sulfide **7** with *m*-CPBA provided the corresponding sulfoxide **17** in 95% yield, and subsequent Pummerer reaction^34,35^ with DIPEA as a base gave rise to **18** and *epi*-**18** in 86% yield as a 1:1 diastereoisomeric mixture. Commonly used Et_3_N was not suitable for the Pummerer reaction because it could act as a nucleophile, leading to formation of a triethylammonium adduct, and thus reduced yield^36^. The *N*-methyl group was introduced by a conventional procedure to afford **5a**, which is an advanced intermediate for the synthesis of albomycin *δ*_1_. The structure of **5a** was verified by X-ray crystallographic analysis (Fig. 3). To achieve the synthesis of albomycin *δ*_2_ and *ε*, a practical access to the imine **20** was required. First, **18** was protected as its TBS ether **19**, which was treated with TPSCl in the presence of DMAP and Et_3_N, followed by NH_4_OH to produce a cytosine derivative in 90% yield over two steps in one pot, and then methylation of N3 delivered **20** in 96% yield. For the subsequent installation of the N4 carbamoyl group, aminolysis of *N*4-phenoxycarbonyl^37^ did not take place due to the steric hindrance of the N3 methyl group. Inspired by the *N*-PMB carbamoylimidazole urea formation reported by Batey et al.^38^, we developed a scalable and efficient protocol to prepare **22b**. In the presence of Et_3_N, **20** was allowed to react with **21** under reflux, giving rise to **22b** in 96% yield. Additionally, Fmoc was chosen as the protecting group for **20**, leading to **22c**, which is a key intermediate for the synthesis of albomycin ε.

**Fig. 4:**
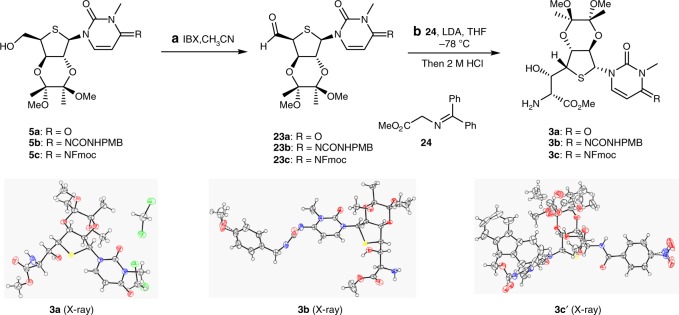
Synthesis of fragments **3a**–**c** and X-ray crystal-structure diagrams of **3a**, **3b**, and **3c'**. Reagents and conditions: **a** IBX (2.0 equiv), CH_3_CN, 55 °C, 5 h; **b** **24** (1.1 equiv), LDA (1.1 equiv), THF, −78 °C, then 2 M HCl, **3a**: 86% (d.r. 4:1), **3b**: 83% (d.r. 3:1), **3c**: 75% (d.r. 3:1), (over 2 steps). *IBX* 2-iodoxybenzoic acid, *LDA* lithium diisopropylamide

With fragments **5a**–**c** in hand, the next challenge was to install the side chain with the desired stereochemistry (Fig. 4). Oxidation of alcohols **5a**–**c** with IBX gave the corresponding aldehydes **23a**–**c** in excellent yields. Side-chain extension via aldol reaction required a delicate choice of protecting groups for the glycine moiety, which is essential for good stereoselectivity. Inspired by Trost’s work^39^, we found that the lithium salt of *N*-(diphenylmethylene) glycine methyl ester could react with aldehydes **23a**–**c** at −78 °C, and subsequent treatment of the condensation products with 2 M aqueous HCl yielded **3a** (86%, 2 steps, d.r. 4:1), **3b** (83%, 2 steps, d.r. 3:1), and **3c** (75%, 2 steps, d.r. 3:1) respectively. The structures of **3a** and **3b** were unambiguously established by X-ray crystallographic analysis, and the molecular structure of **3c** was substantiated by X-ray crystallography of its benzoyl derivative **3c'** (Fig. 4).

**Fig. 5:**
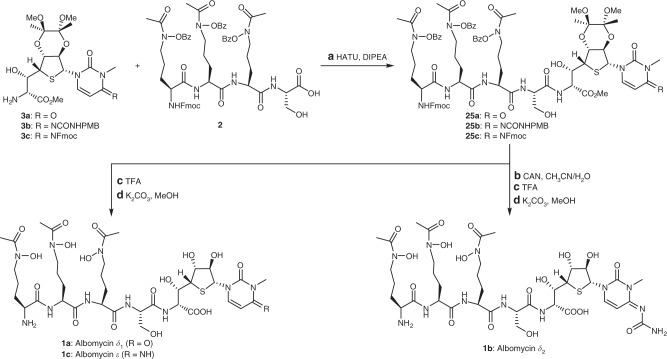
Total synthesis of albomycins *δ*_1_, *δ*_2_, and *ε*. Reagents and conditions: **a** HATU (1.5 equiv), DIPEA (2.0 equiv), DMF, −15 °C, 2 h, **25a**: 93%, **25b**: 91%, **25c**: 90%; **b** CAN (10.0 equiv), CH_3_CN/H_2_O, 30 min; **c** TFA, H_2_O, 1 h; **d** K_2_CO_3_, MeOH, H_2_O, **1a**: 80% (over 2 steps), **1b**: 72% (over 3 steps), **1c**: 75% (over 2 steps). *CAN* ceric ammonium nitrate

With key fragments **2** and **3a**–**c** in place, their assembly into albomycins (**1a**–**c**) was embarked (Fig. 5). Thionucleosides **3a**–**c** were first condensed with tetrapeptide **2** to afford **25a**–**c** in excellent yields. The final deprotection steps from **25a**–**c** to the final albomycins (**1a**–**c**) required delicate adjustments of the reaction conditions in order to preserve the sensitive amide bond^40^ on the cytosine of **1b** and **1c**. First, oxidative deprotection of the *N*-PMB group in **25b** proceeded smoothly. Addition of CF_3_CO_2_H and H_2_O to the resulting crude product removed the bisacetal moiety within the molecules. The reaction time for these two deprotection steps needed to be carefully controlled, otherwise it would lead to hydrolysis of the imine. After surveying numerous reaction conditions, we found that aqueous K_2_CO_3_ (150 mg/mL) could efficiently remove the remaining protecting benzoyl, methyl ester, and Fmoc groups in one pot to afford albomcyin *δ*_2_ (**1b**). These three deprotection steps (**25b** to **1b**) were carried out without purifying the intermediates and the final product albomycin *δ*_2_ (**1b**) was purified with Sephadex^TM^ G-15. We subsequently exploited the flexibility of this sequential deprotection. To our pleasure, following the same protocals, both albomycins *δ*_1_ (**1a**) and *ε* (**1c**) were obtained in good yields. It’s worth noting that albomcyin *ε* (**1c**) was not stable in D_2_O, and slowly converted to albomycin *δ*_1_ (**1a**) over time. Albomycin *δ*_2_ (**1b**) was much more stable than albomcyin *ε* (**1c**), and remained unchanged in D_2_O at 4 °C for over one month.

### Biological assessment

As the three naturally occurring albomycins became synthetically accessible, we evaluated their potential as therapeutic agents and determined their MIC values against three Gram-positive and three Gram-negative bacteria species following the protocol from the Clinical and Laboratory Standards Institute (CLSI). Commercial antibiotic, ciprofloxacin, was used as a positive control in the MIC determination experiment. As shown in Table 1, albomycin *δ*_1_ (**1a**) and *δ*_2_ (**1b**) exhibited 8-fold more potency than ciprofloxacin against *S. pneumoniae* ATCC 49619. **1b** inhibited *S. aureus* USA 300 strain NRS384^41^, a virulent methicillin resistant MRSA strain, with an MIC of 0.125 µg/mL, and was 16-fold more potent than ciprofloxacin. **1b** exhibited an MIC value of 0.5 µg/mL against *Bacillus subtilis* ATCC 6633, which was less active compared to ciprofloxacin. As for *Escherichia coli* BJ 5183^42^, **1b** showed about 8-fold higher potency than **1a**. Particularly impressive is the potency of compound **1a** towards the fastidious *Neisseria gonorrhoeae* ATCC 49226 with a 3.9 ng/mL MIC value while **1b** was completely inactive. These results led us to conclude that the C4 substituent of nucleobase in albomycins played an important role rendering their antibacterial activity. Biochemical analysis of albomycin nucleobase analogs will test this hypothesis. All three albomycins displayed no activity towards Gram-negative bacteria *Salmonella typhi*, and albomycin *ε* (**1c**) was inactive to all these strains, which was previously unknown.

**Table 1:** MIC values (μg/mL) of albomycins *δ*_1_ (**1a**), *δ*_2_ (**1b**), and *ε* (**1c**)

Entry	Gram-(+)			Gram-(−)		
	*S. pneumoniae* ATCC 49619	*S. aureus* USA 300 NRS 384	*B. subtilis* ATCC 6633	*E. coli* BJ 5183	*N. gonorrhoeae* ATCC 49226	*Salmonella typhi*
Ciprofloxacin	0.5	2	0.0625	0.0039	0.0039	>512
**1a**	0.0625	>512	>512	0.25	0.0039	>512
**1b**	0.0625	0.125	0.5	0.0312	>512	>512
**1c**	>512	>512	>512	>512	>512	>512

World Health Organization (WHO) published a priority list of antibiotic-resistant pathogenic bacteria for developing new and effective antibiotic treatments. Both *S. pneumoniae and S. aureus* are on the list due to their increasing multidrug resistance. Ciprofloxacin, vancomycin and penicillin G are on the WHO Model List of Essential Medicines (EML). Penicillin G is among the first medications against bacterial infections and vancomycin has been hailed as the last line of defense. To explore the potential to develop albomycin *δ*_2_ into an effective antibiotic, we screened albomycin *δ*_2_ against a random collection of 27 clinical *S. pneumoniae* and *S. aureus* isolates (three of them are MRSA strains), and compared it with ciprofloxacin, vancomycin, and penicillin G (Table 2) in inhibiting the growth of these clinical pathogens. All these *S. pneumoniae* and *S. aureus* strains were freshly isolated from patients in clinic. As shown in Table 2, albomycin *δ*_2_ exhibited excellent anti-*S. pneumoniae* and *S. aureus* activities better than the other three antibiotics in most cases, while many of these strains displayed severely natural resistance to penicillin G. The MIC values of albomycin *δ*_2_ were well below those of ciprofloxacin, vancomycin, and penicillin G, and in a number of cases reaching 1000 times lower. The influence of iron concentration on the antibacterial activity was also studied by conducting assays in iron-rich and iron-deficient media (Table 3). The antibiotic activity of albomycin *δ*_2_ against *S*. *pneumoniae* strains was significantly increased in iron-deficient media, which most closely mimics the physiological situation in a human host, wherein iron is sequestered in macromolecules such as heme^43^. Two isolates, strains S15 and S29, which showed the most resistance to albomycin *δ*_2_ in iron-rich media, became highly susceptible under iron-depleted conditions. The MIC values of albomycin *δ*_2_ against *S*. *aureus* and *E*. *coli* strains were not influenced by iron concentration. The three control antibiotics did not show any dependence on iron concentration in any of the strains tested. These results suggest that albomycin *δ*_2_ is a promising antibiotic candidate for further clinical drug development.

**Table 2:** MIC values (µg/mL) of albomycin *δ*_2_ (**1b**) against clinical isolates of *S. pneumoniae* and *S. aureus*

Strains	1b	Cipro.	Vanco.	Peni.	Strains	**1b**	Cipro.	Vanco.	Peni.
*S. pneumoniae* 30	0.0039	1	≤1	≥2	*S. pneumoniae* S13	2	0.5	≤1	≥2
*S. pneumoniae* 33	0.0039	1	0.5	≤0.0625	*S. pneumoniae* S15	8	0.5	0.25	≥2
*S. pneumoniae* 10	0.0078	0.5	0.5	≥2	*S. pneumoniae* 29	16	>4	≤1	≥2
*S. pneumoniae* 28	0.0078	1	≤1	1	*S. aureus* S13	0.0625	0.25	1	≥0.5
*S. pneumoniae* 31	0.0078	0.5	0.5	1	*S. aureus* S19	0.0625	0.25	1	≥0.5
*S. pneumoniae* 32	0.0078	1	0.5	≤0.0625	*S. aureus* S15	0.125	1	1	≥0.5
*S. pneumoniae* 42	0.0078	1	0.5	≥2	*S. aureus* S17	0.125	0.25	1	≥0.5
*S. pneumoniae* S14	0.0078	0.5	0.25	0.5	*S. aureus* S21	0.125	0.5	1	≥0.5
*S. pneumoniae* 26	0.0156	1	≤1	≥2	*S. aureus* S12 (MRSA)	0.25	0.5	1	≥0.5
*S. pneumoniae* 43	0.0156	1	0.5	≥2	*S. aureus* S14 (MRSA)	0.25	0.25	1	≥0.5
*S. pneumoniae* 44	0.0156	1	0.5	≤0.0625	*S. aureus* S16	0.25	>4	1	≥0.5
*S. pneumoniae* 09	0.0312	1	0.5	≥2	*S. aureus* S18 (MRSA)	0.25	0.5	2	≥0.5
*S. pneumoniae* 27	0.0625	0.5	≤1	≥2	*S. aureus* S22	0.25	0.5	1	≥0.5
*S. pneumoniae* 23	0.25	1	≤1	0.5					

These clinical pathogens were freshly isolated from Children’s Hospital of Chongqing Medical University, Chongqing, China

*Cipro.* Ciprofloxacin, *Vanco.* Vancomycin, *Peni.* Penicillin G

**Table 3:** MIC values (μg/mL) of albomycin *δ*_2_ (**1b**) against *S. pneumoniae, S. aureus* and *E. coli* strains in MHII and MHII-Fe

Strains	Albomycin *δ*_2_ (1b)		Ciprofloxacin		Vancomycin		Penicillin G	
	MHII	MHII-Fe	MHII	MHII-Fe	MHII	MHII-Fe	MHII	MHII-Fe
*S*. *pneumoniae* ATCC 49619	0.0625	0.0078	0.5	0.5	0.25	0.25	0.5	0.5
*S*. *pneumoniae* S14	0.0078	0.0039	0.5	0.5	0.25	0.25	0.5	0.5
*S*. *pneumoniae* S15	8	0.125	0.5	0.5	0.25	0.25	≥2	≥2
*S*. *pneumoniae* 29	16	0.5	>4	>4	≤1	≤1	≥2	≥2
*S. aureus* ATCC 29213	0.5	0.5	0.5	1	2	2	≥0.5	≥0.5
*S*. *aureus* S17	0.125	0.125	0.25	0.25	1	1	≥0.5	≥0.5
S. *aureus* S19	0.0625	0.0625	0.25	0.25	1	1	≥0.5	≥0.5
*E. coli* ATCC 25922	0.25	0.25	0.0312	0.0312	–	–	–	–
*E. coli* BJ 5183	0.0312	0.0312	0.0039	0.0039	–	–	–	–

*MHII* Mueller–Hinton broth II, *MHII-Fe* Mueller–Hinton broth II + 100 μM 2,2′-bipyridine

## Discussion

In summary, the successful execution of the convergent strategy has led to the total synthesis of albomycins *δ*_1_ (**1a**), *δ*_2_ (**1b**), and *ε* (**1c**). Antibacterial assessment of albomycins revealed that C4 substituent on the nucleobase in albomycin plays an essential role in their antibacterial activity. Albomycin *δ*_2_ exhibited potent antimicrobial activities against clinical *S. pneumoniae* and *S. aureus* isolates including MRSA. Further studies to evaluate albomycin *δ*_2_ as a potentially effective and safe antibiotic are ongoing.

## Methods

### General

All air-sensitive and water-sensitive reactions were carried out under a nitrogen atmosphere with dry solvents under anhydrous conditions, unless otherwise noted. Tetrahydrofuran (THF) was distilled over sodium and benzophenone, dichloromethane (CH_2_Cl_2_), *N*, *N*-dimethylformamide (DMF), triethylamine (Et_3_N), and *N*,*N*-diisopropylamine (DIPEA) over calcium hydride. All other solvents, as well as starting materials and reagents were obtained from commercial sources and used without further purification. Reactions were monitored by analytical thin-layer chromatography (TLC) on Merck silica gel 60 F_254_ plates (0.25 mm), visualized by ultraviolet light and/or by staining with phosphomolybdic acid in EtOH. Retention factor (R_*f*_) values were measured using a 5 × 2 cm TLC plate in a developing chamber containing the solvent system described. Yields refer to the isolated yields after silica gel flash column chromatography, unless otherwise stated. ^1^H NMR spectra were obtained on an Agilent 400MR or 600MR DD2 spectrometer at ambient temperature. Chemical shifts were reported in parts per million (ppm), relative to either a tetramethylsilane (TMS) internal standard or the signals due to the solvent. ^13^C NMR spectra were obtained with proton decoupling on an Agilent 400MR or 600MR DD2 (100 MHz or 150 MHz) spectrometer and were reported in ppm with residual portium for internal standard. Multiplicity is defined as: s = singlet; d = doublet; t = triplet; q = quartet; m = multiplet, br = broad, or combinations of the above. Coupling constants (J) are reported in Hertz. High resolution mass spectra were obtained on a Bruker SolariX 7.0 T spectrometer. Melting point was determined by WRS-2A Digital Melting Point Apparatus. Optical rotations were measured with a Rudolph polarimeter. Crystallographic data were obtained from a single-crystal X-ray diffractometer.

### Further experimental data

For NMR spectra of the synthesized compounds, see Supplementary Figs. 1–62. For detailed experimental procedures, see Supplementary Figs. 63–92, and Supplementary Methods. For the comparisons of ^1^H and ^13^C NMR spectroscopic data of the natural and synthetic albomycin *δ*_2_, see Supplementary Tables 1 and 2. For the crystallographic data of compounds **3a**, **3b**, **3c'**, and **5a**, see Supplementary Figs. 93–96, and Supplementary Tables 3–6.

### Clinical pathogens isolation and identification

The clinical pathogens were isolated according to National Guide to Clinical Laboratory Procedures and characterized by culturing in the specifically appropriate media followed by the VITEK mass spectrometry microbial identification system (bioMerieux, France). Antimicrobial susceptibility was performed for all tested clinical bacterial strains by 96-well microdilution method following Clinical and Laboratory Standards Institute (CLSI) recommendations.

### Susceptibility testing

Minimal inhibitory concentration (MIC) assays were adapted according to the recommendations of the Clinical and Laboratory Standard Institute (CLSIM07). For *N. gonorrhoeae*, MICs were determined by the standard agar dilution method on GC medium base with Isovitalex (Topbio, Shandong, China) at 37 °C with 5% CO_2_; for *S. pneumoniae*, MICs were tested using 96-well microdilution plates in Mueller–Hinton broth II with 5% horse-blood (Solarbio, Beijing, China) at 37 °C with 5% CO_2_; other strains were detected by the standard 96-well microdilution method in Mueller–Hinton broth II (Solarbio, Beijing, China) at 37 °C. Dilutions of the compound were made in quadruplicate in 96-well culture dishes. Strains were taken from an exponentially growing culture and diluted to 5 × 10^5^ CFU/mL. The bacteria were cultured in the presence of tested compounds for about 20 h and bacterial growth was monitored visually following the CLSI guidelines. *S. aureus* ATCC 29213, *S. pneumoniae* ATCC 49619, and *E.coli* ATCC 25922 were used as quality control strains, which were purchased from National Center for Clinical Laboratories.

## Electronic supplementary material

Supplementary Information

Peer Revew File

## Data Availability

The X-ray crystallographic coordinates for structures reported in this article have been deposited at the Cambridge Crystallographic Data Centre (CCDC), under deposition number CCDC 1839504 for **5a**, CCDC 1839506 for **3a**, CCDC 1839508 for **3b**, and CCDC 1839510 for **3c'**. These data can be obtained free of charge from The CCDC via (https://www.ccdc.cam.ac.uk/data_request/cif). The authors declare that other data supporting the findings of this study are available within the paper and its supplementary information files and also are available from the corresponding author upon request.
