# Supplementary material for: Total synthesis and antimicrobial evaluation of natural albomycins against clinical pathogens
Source: Nat Commun. 2018 Sep 4;9:3445. doi: 10.1038/s41467-018-05821-1 (PMC6123416; doi:10.1038/s41467-018-05821-1)
Supplement: Supplementary file 1 — Supplementary Information [file 41467_2018_5821_MOESM1_ESM.pdf]

Total Synthesis and Antimicrobial Evaluation of Natural Albomycins against Clinical Pathogens

Lin *et al*.

Supplementary Figures

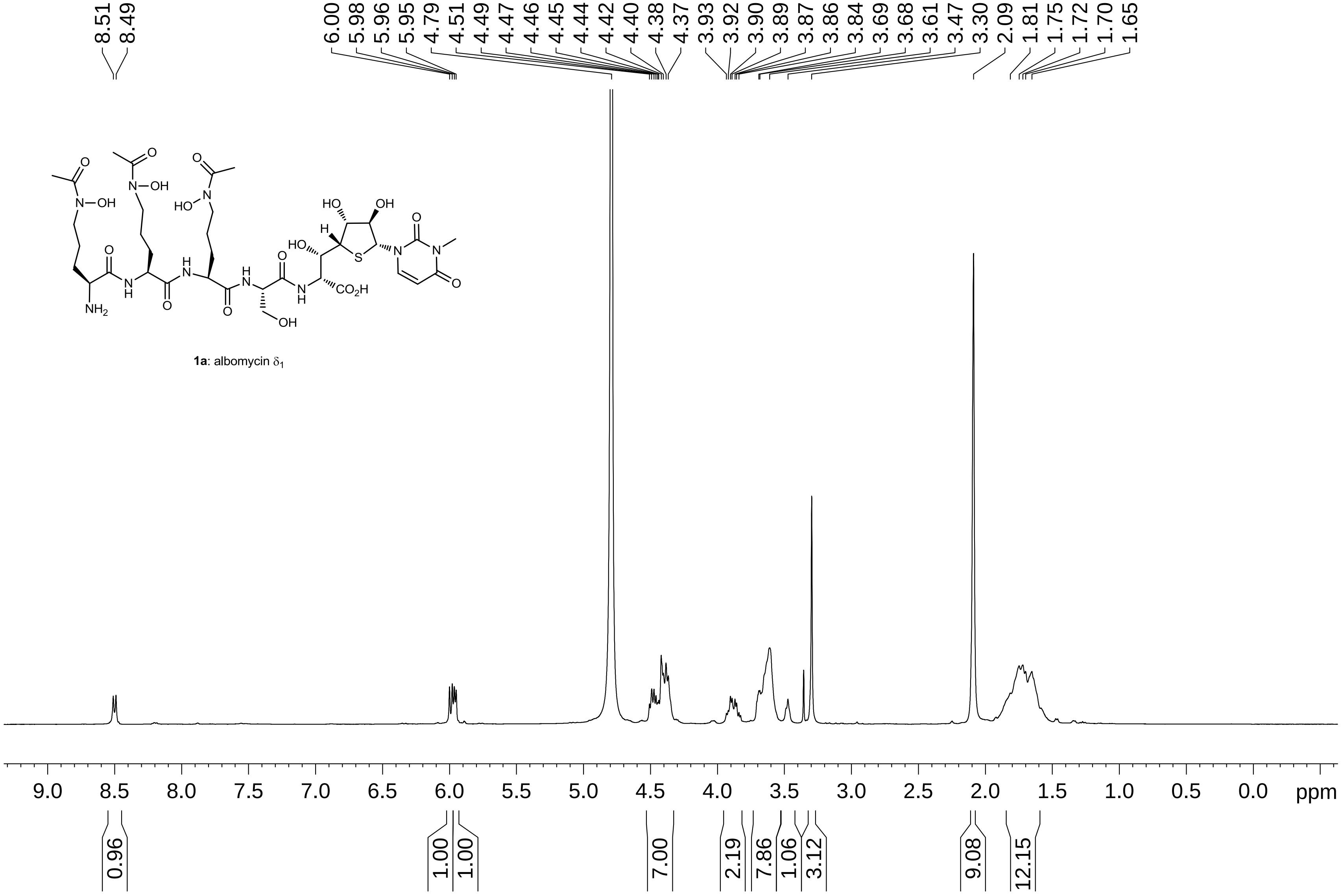

Supplementary Figure 1: ^{1}H NMR for compound **1a** (D_2O, 400 MHz).

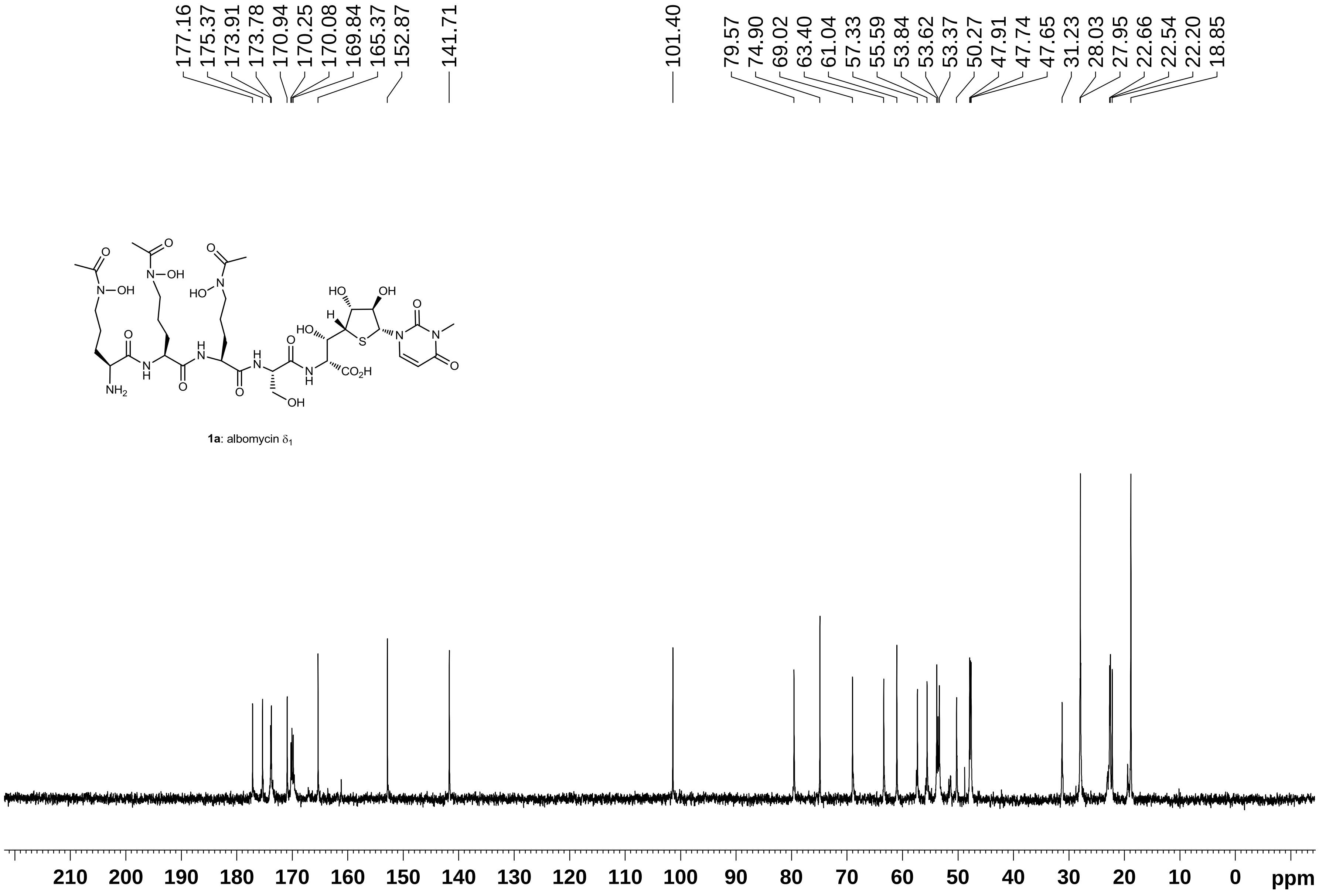

Supplementary Figure 2: ^{13}C NMR for compound **1a** (D_2O, 100 MHz).

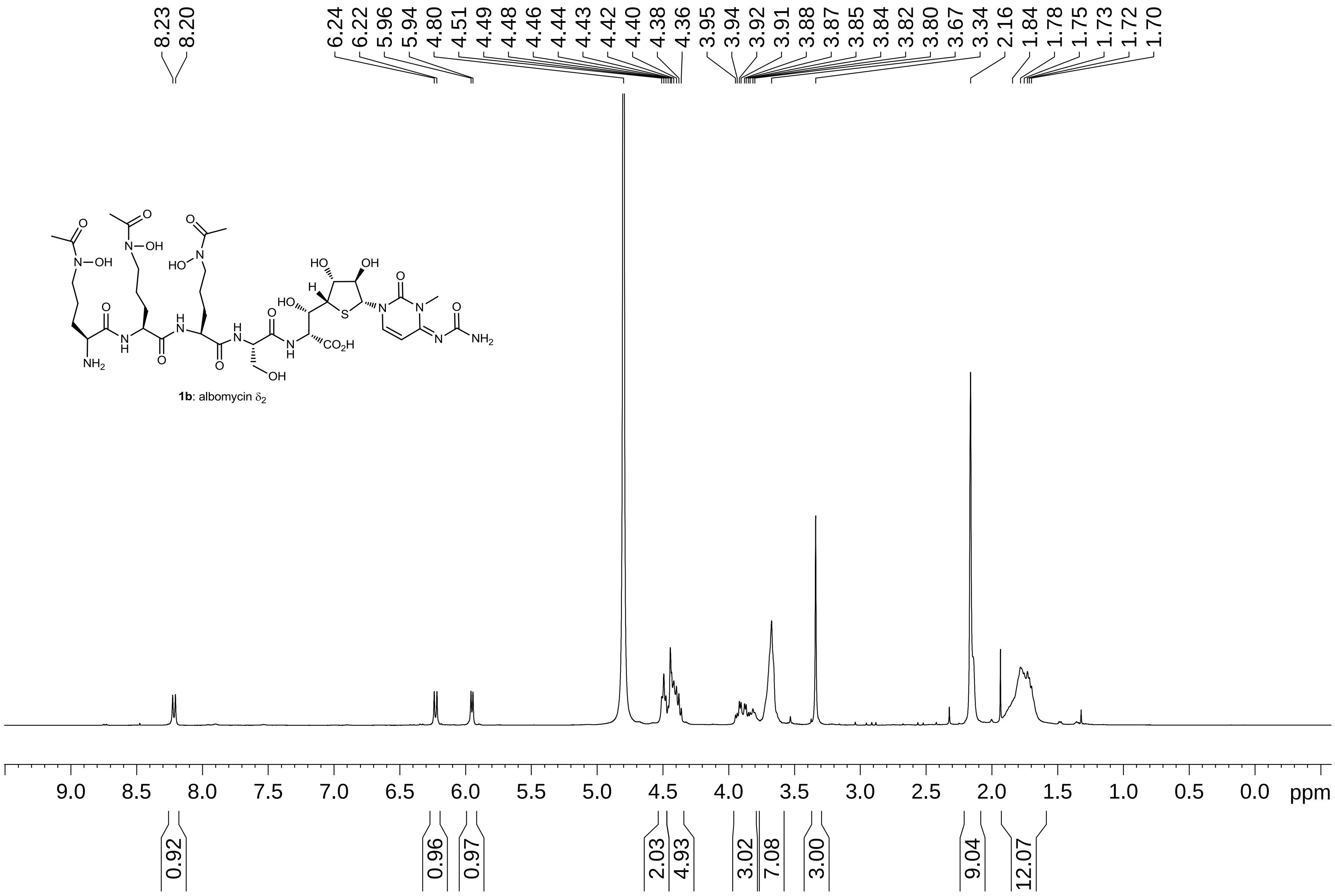

Supplementary Figure 3: ^{1}H NMR for compound **1b** (D_2O, 400 MHz).

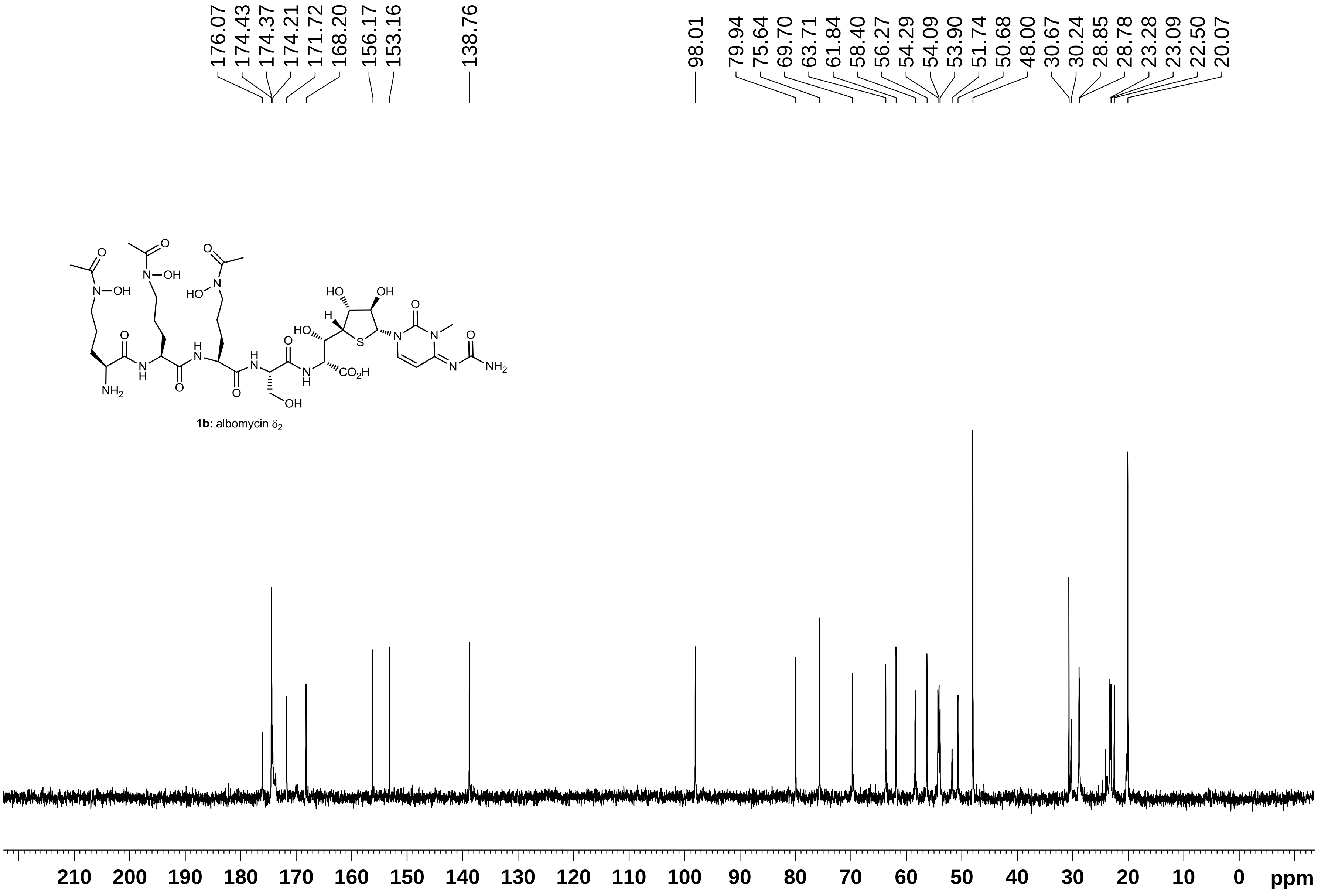

Supplementary Figure 4: ^{13}C NMR for compound **1b** (D_2O, 100 MHz).

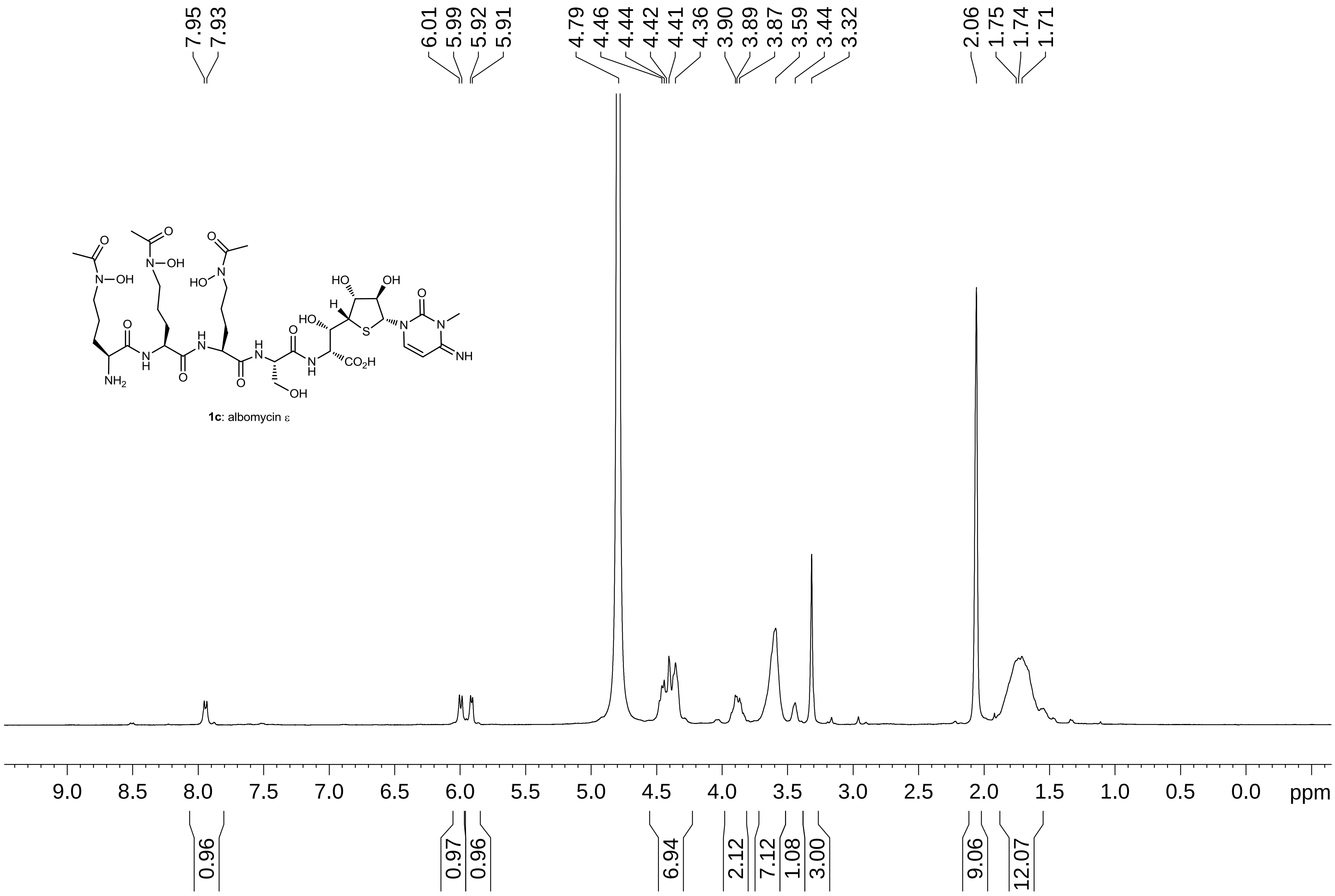

1c: albomycin ε

Supplementary Figure 5: ^{1}H NMR for compound **1c** (D_2O, 400 MHz).

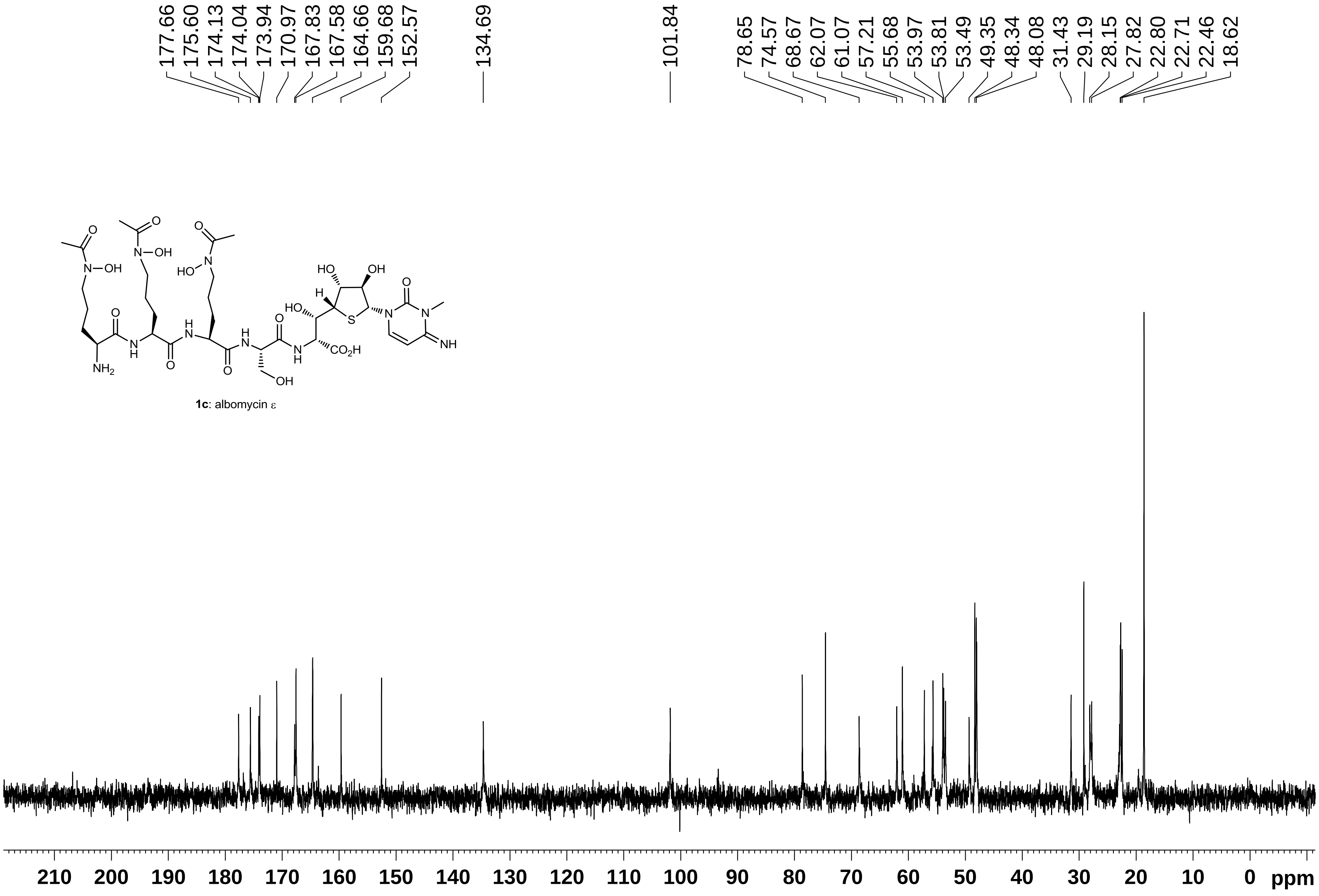

Supplementary Figure 6: ^{13}C NMR for compound **1c** (D_2O, 100 MHz).

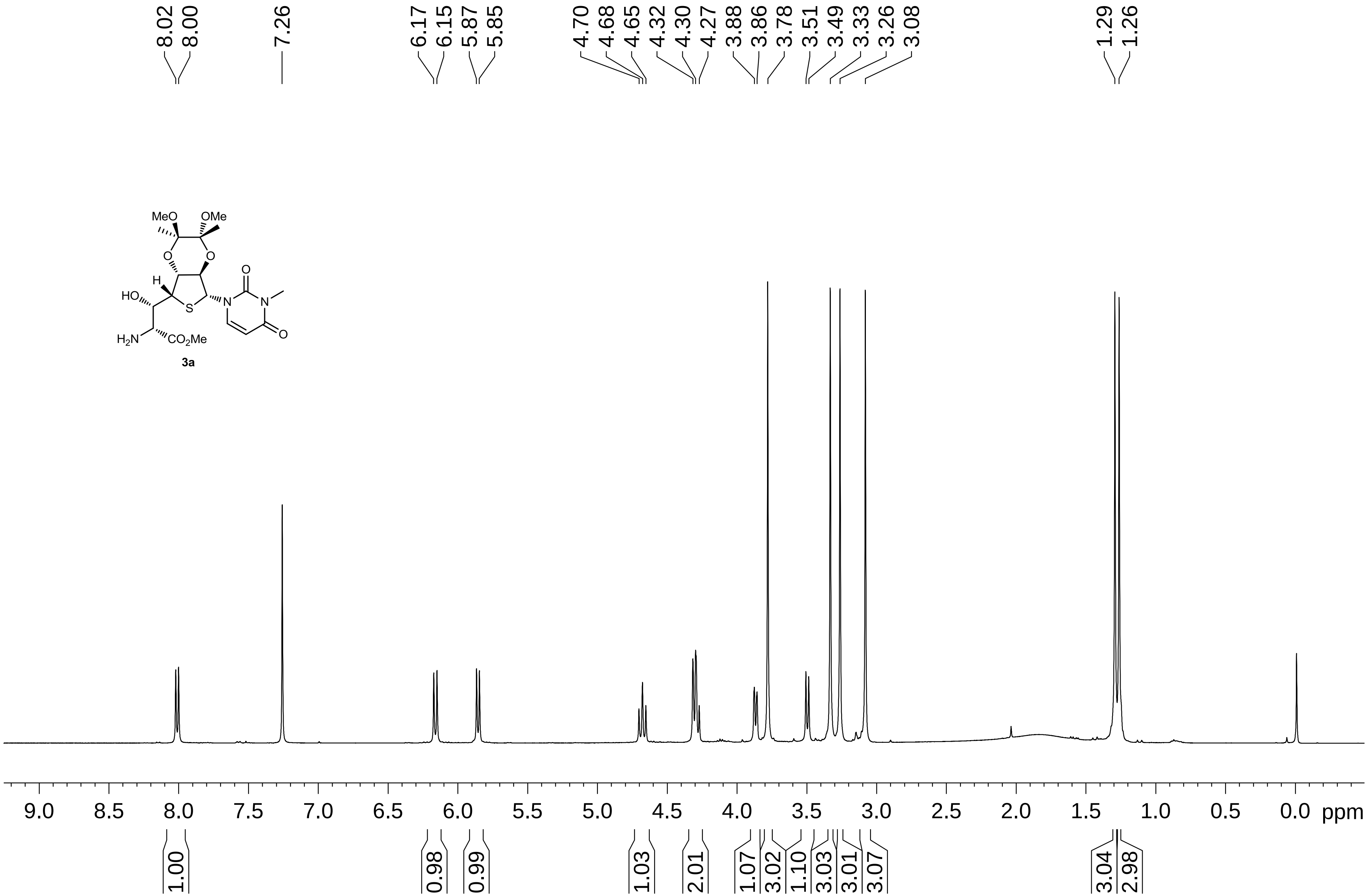

Supplementary Figure 7: ^{1}H NMR for compound **3a** ($CDCl_3$, 400 MHz).

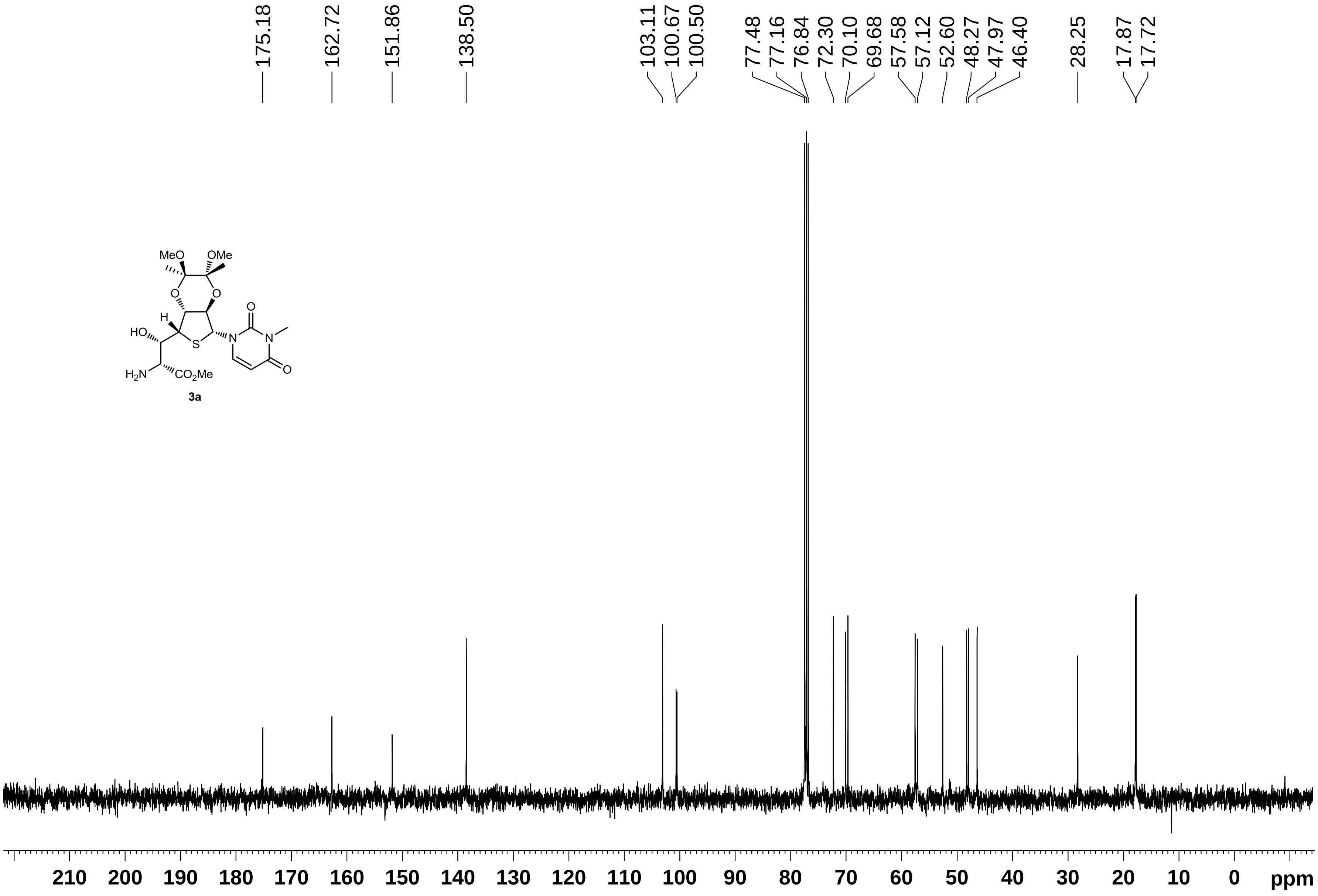

Supplementary Figure 8: ^{13}C NMR for compound **3a** ($CDCl_3$, 100 MHz).

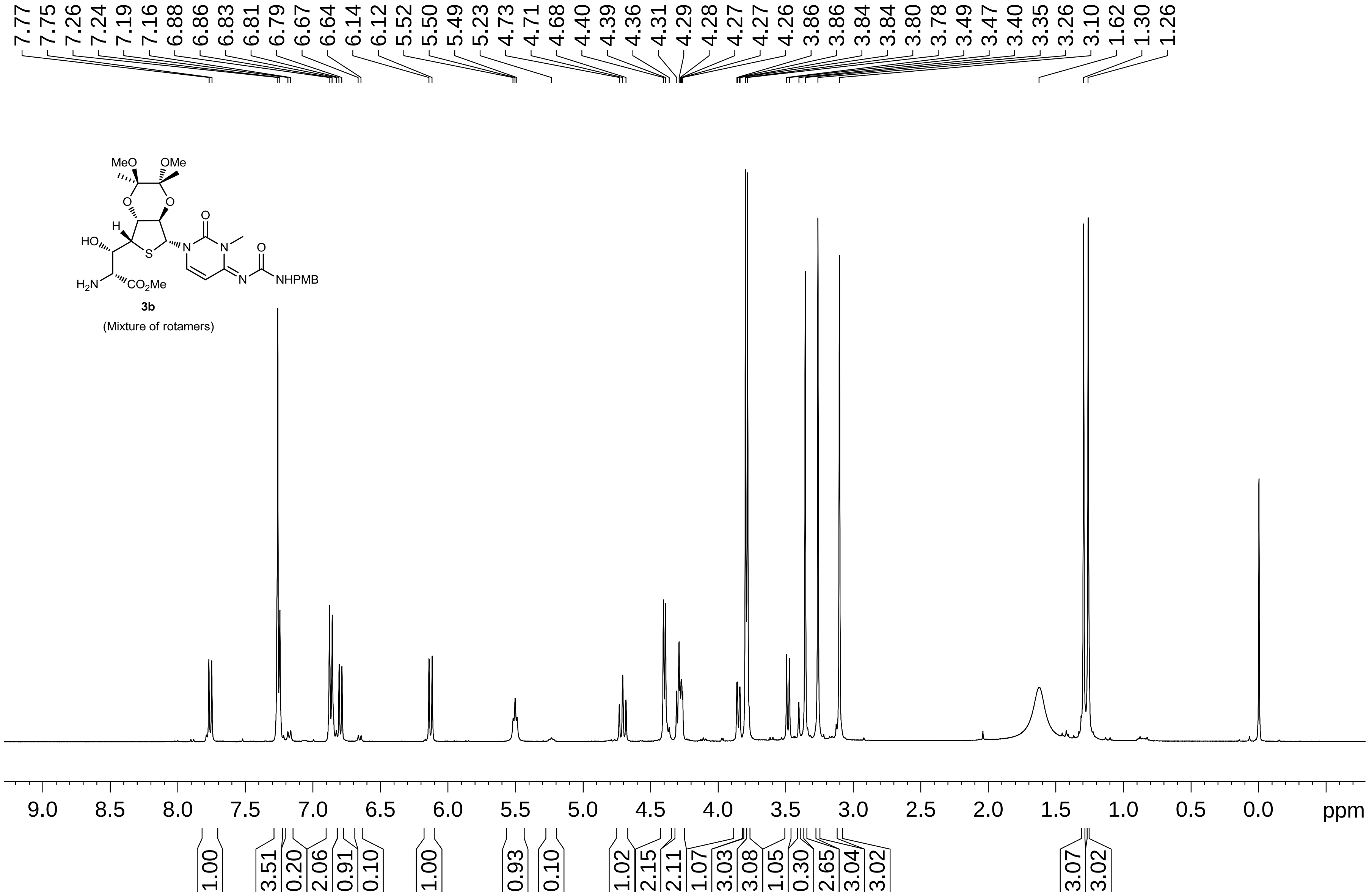

Supplementary Figure 9: ^{1}H NMR for compound **3b** ($CDCl_3$, 400 MHz).

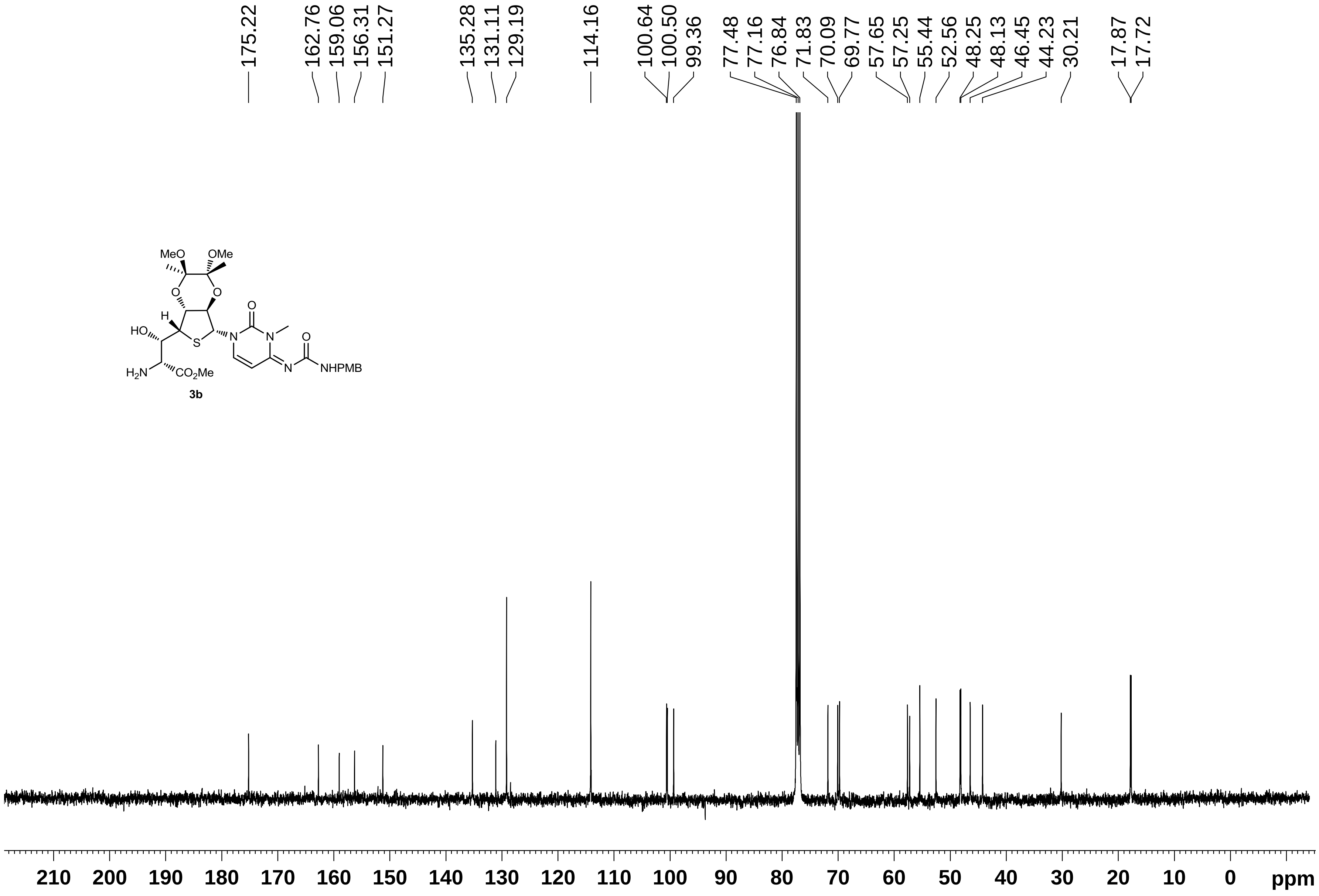

Supplementary Figure 10: ^{13}C NMR for compound **3b** ($CDCl_3$, 100 MHz).

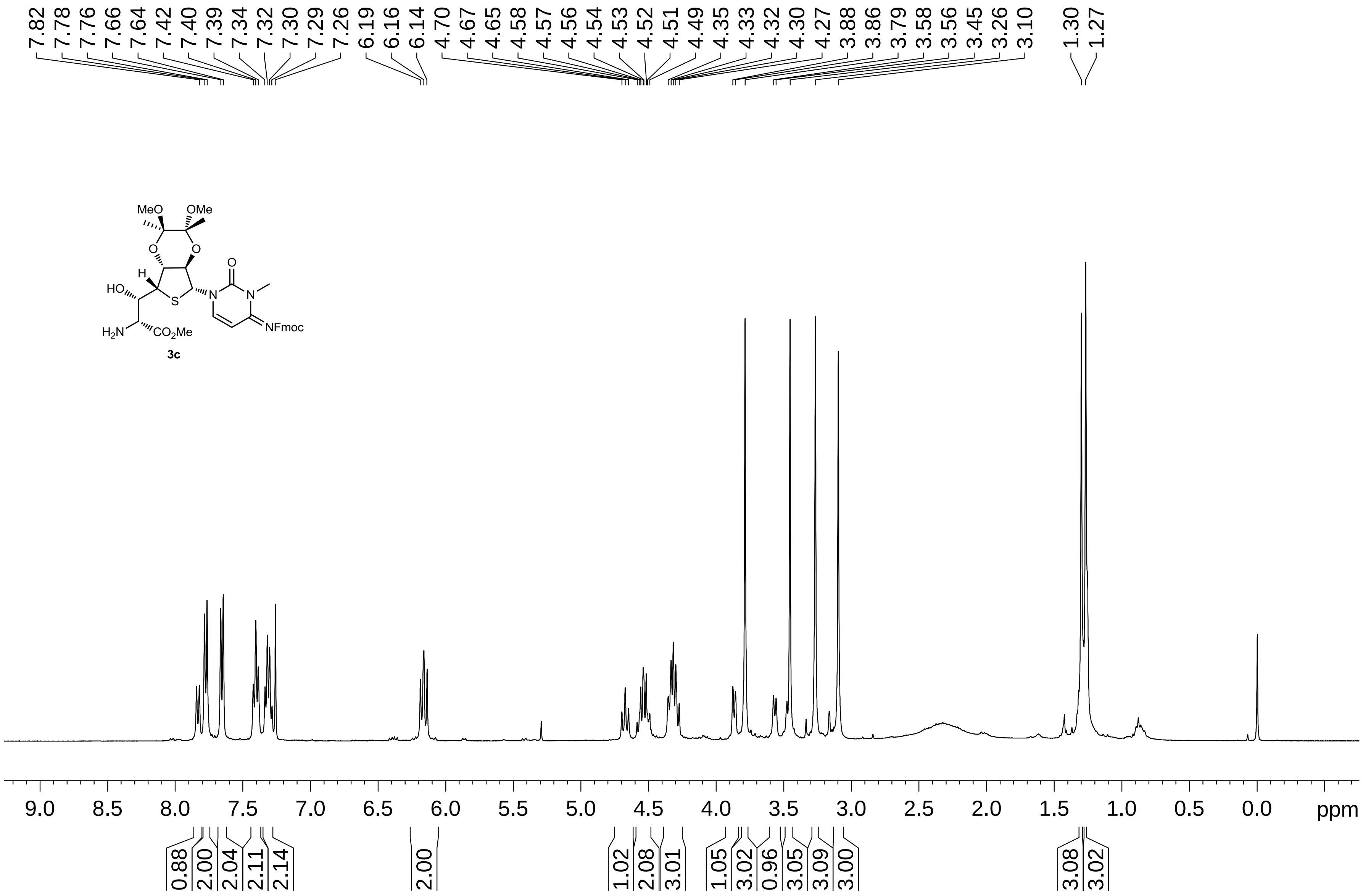

Supplementary Figure 11: ^{1}H NMR for compound **3c** ($CDCl_3$, 400 MHz).

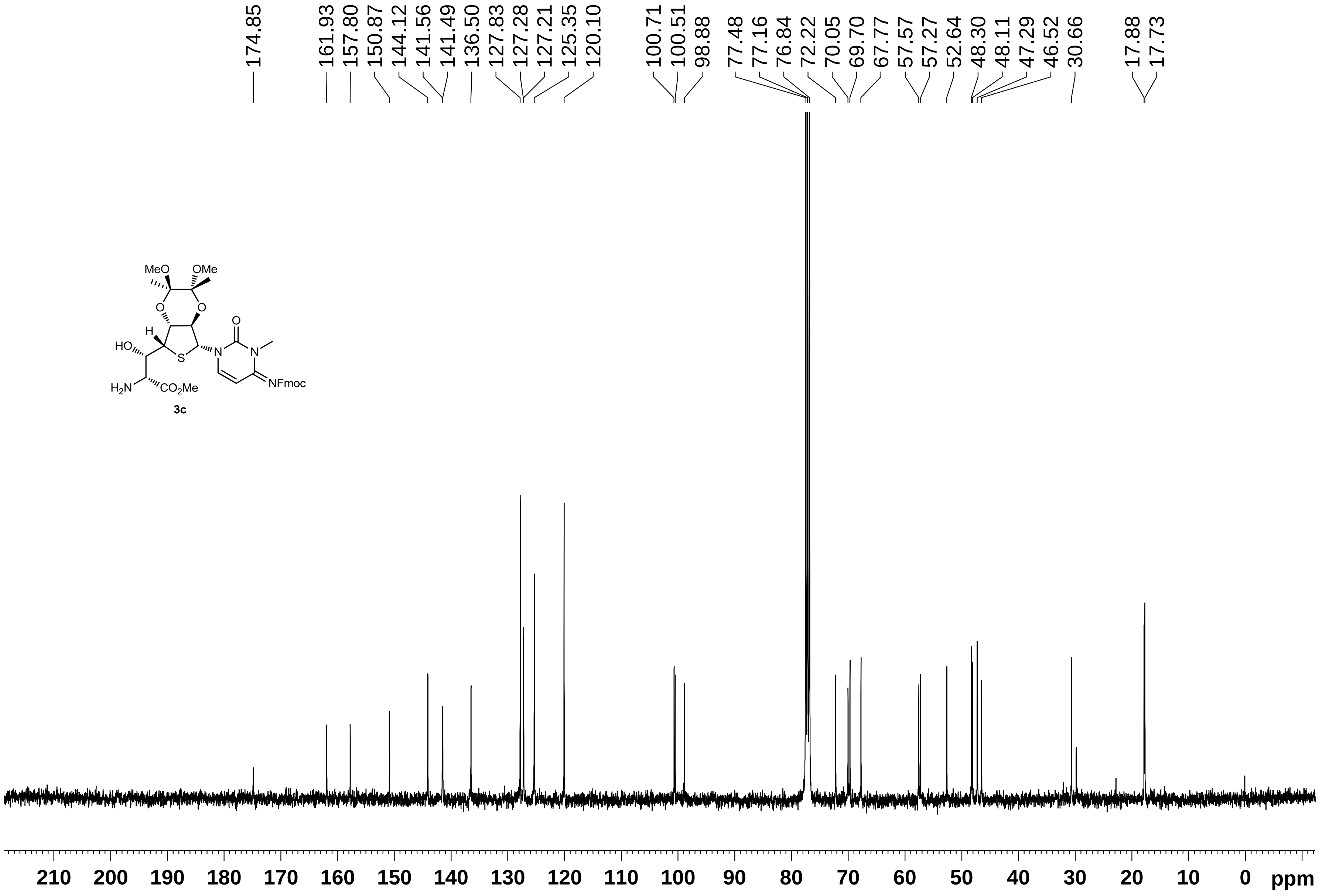

Supplementary Figure 12: ^{13}C NMR for compound **3c** ($CDCl_3$, 100 MHz).

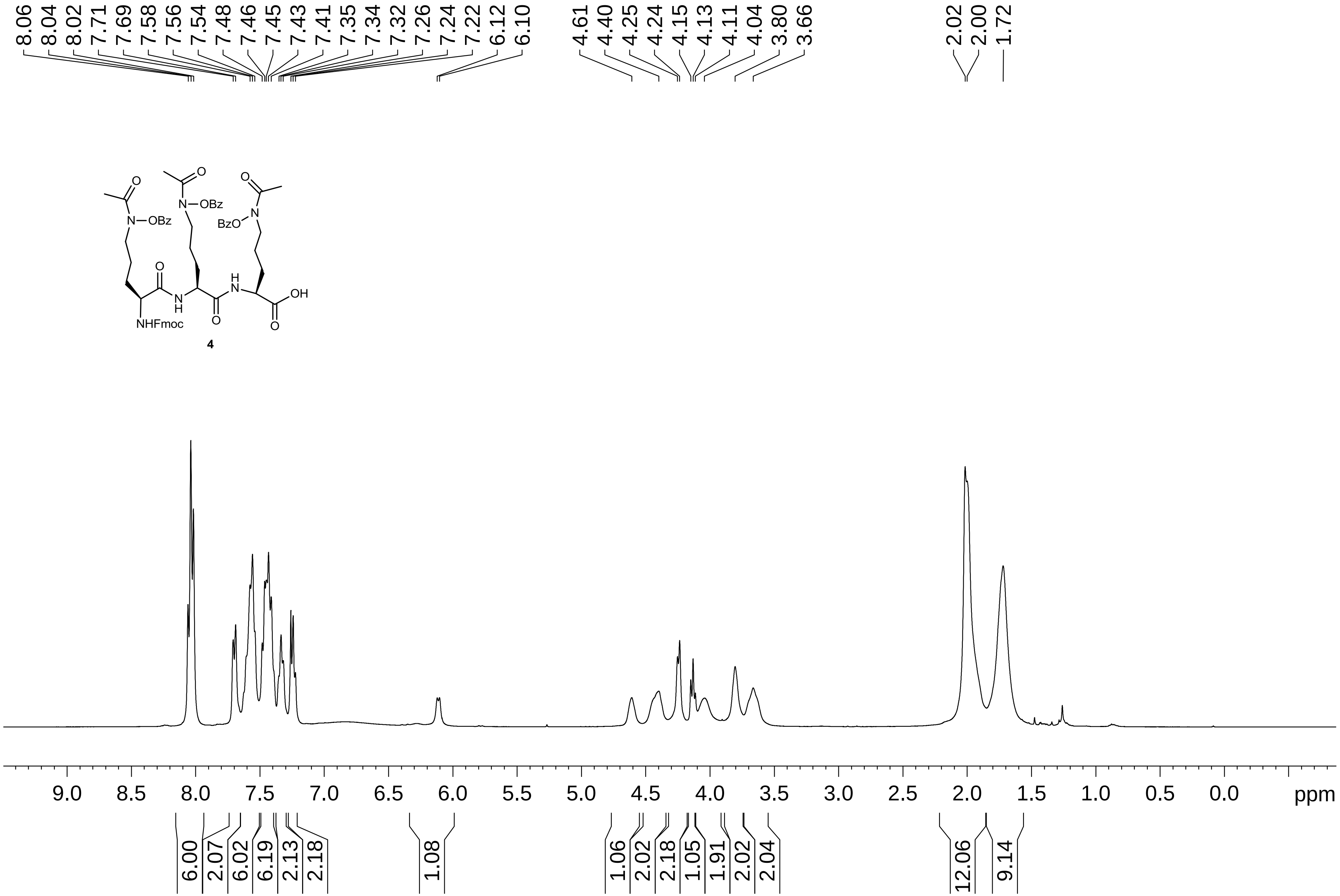

Supplementary Figure 13: ^{1}H NMR for compound **4** ($CDCl_3$, 400 MHz).

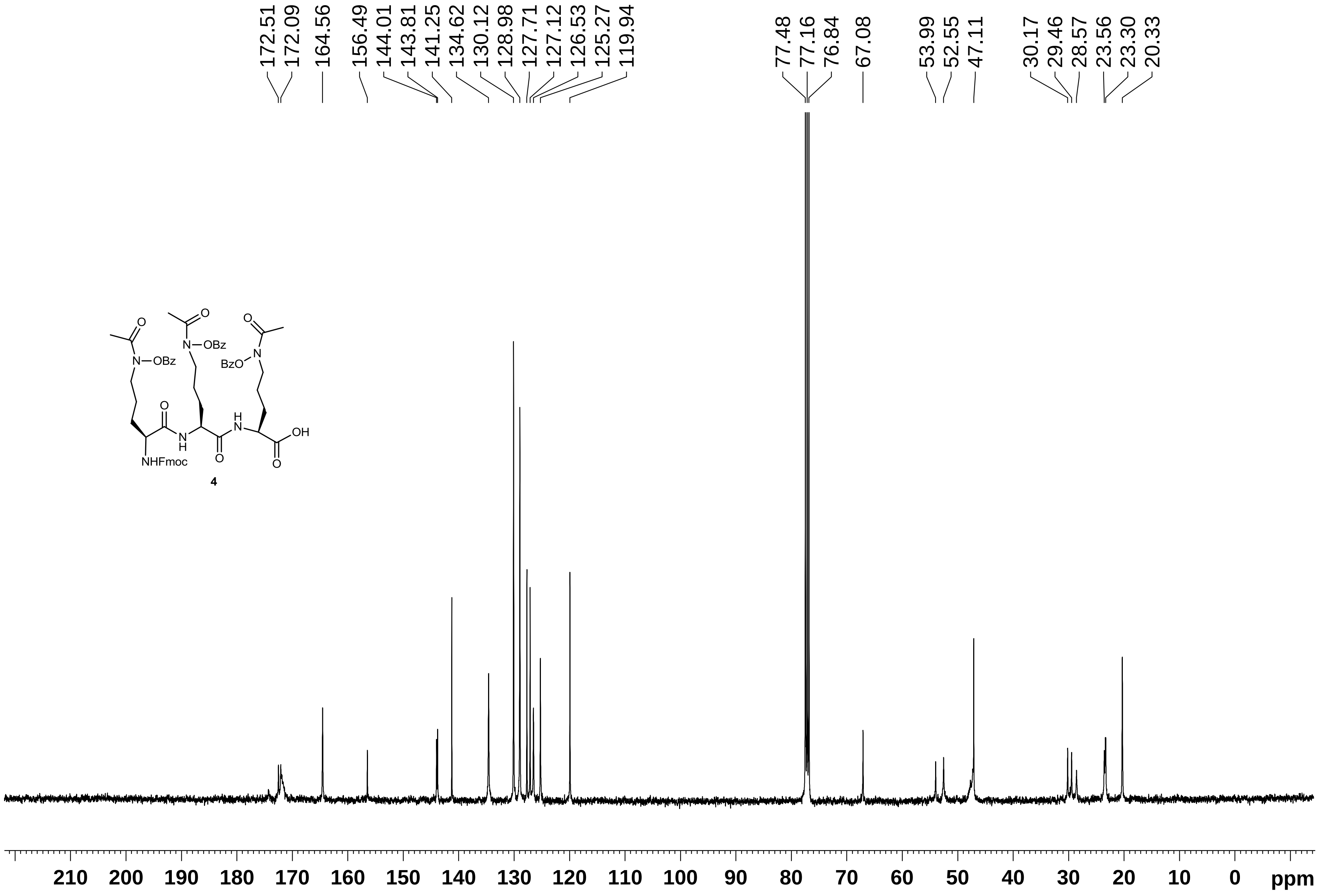

Supplementary Figure 14: ^{13}C NMR for compound **4** ($CDCl_3$, 100 MHz).

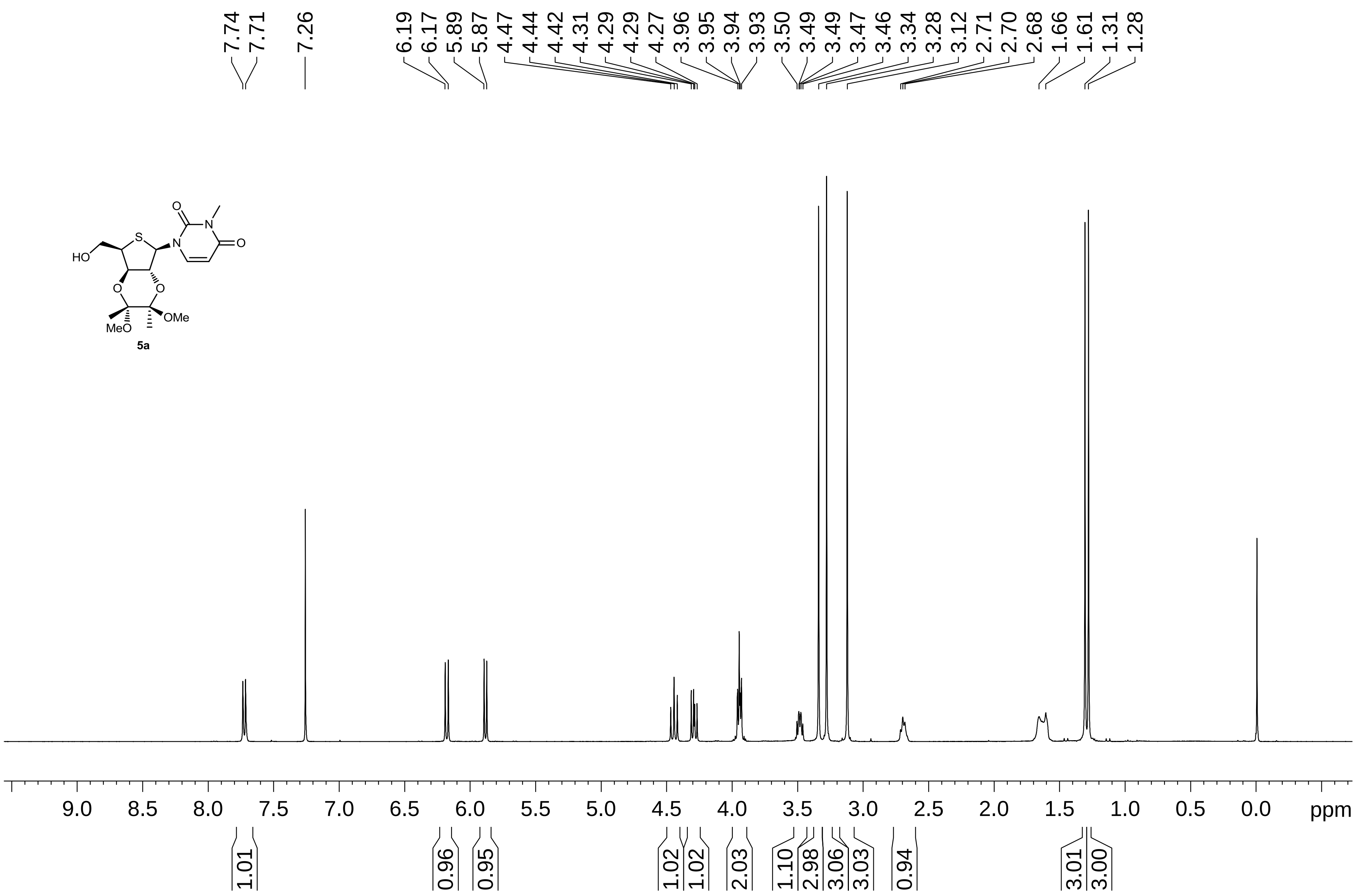

Supplementary Figure 15: ^{1}H NMR for compound **5a** ($CDCl_3$, 400 MHz).

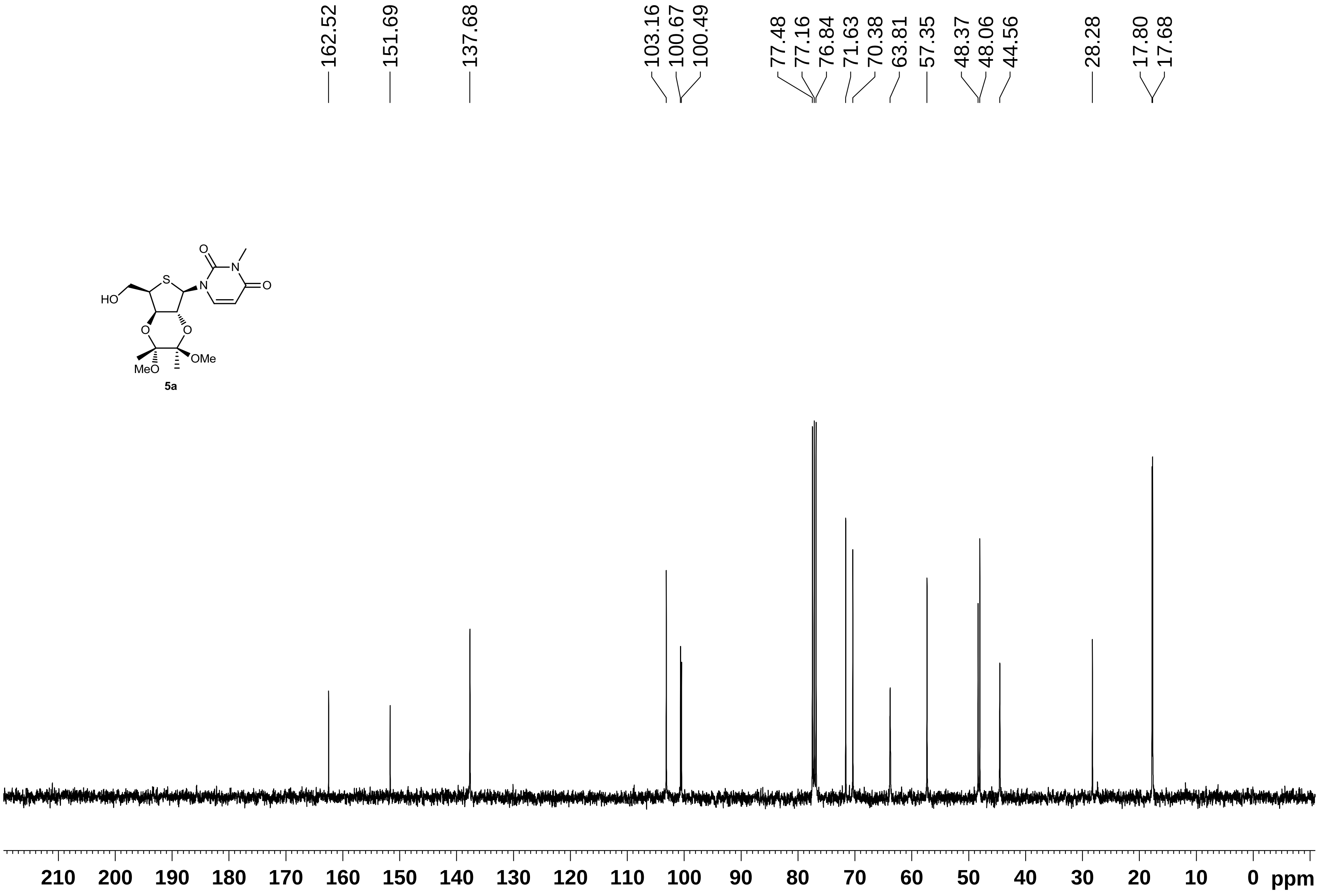

Supplementary Figure 16: ^{13}C NMR for compound **5a** ($CDCl_3$, 100 MHz).

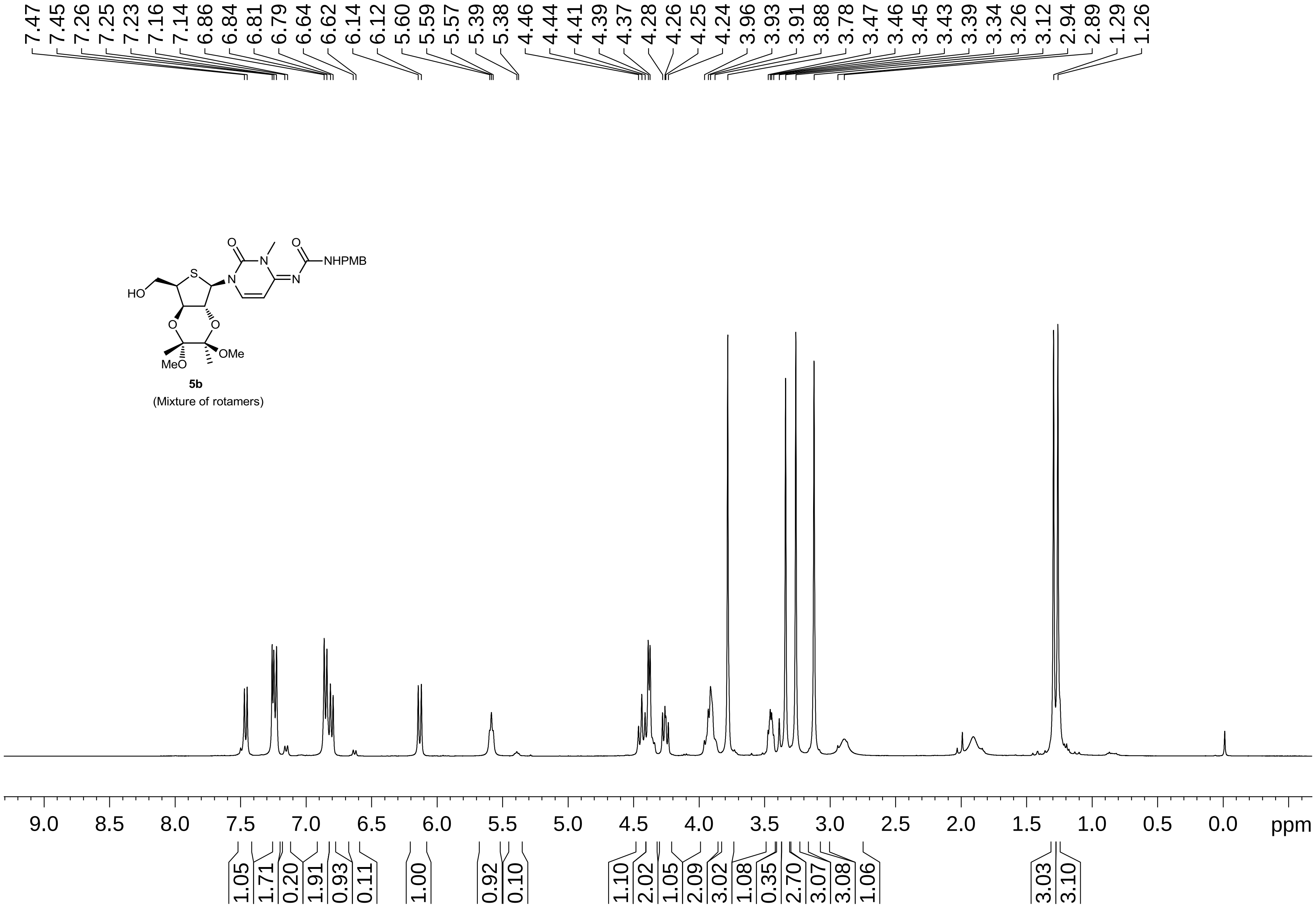

Supplementary Figure 17: ^{1}H NMR for compound **5b** ($CDCl_3$, 400 MHz).

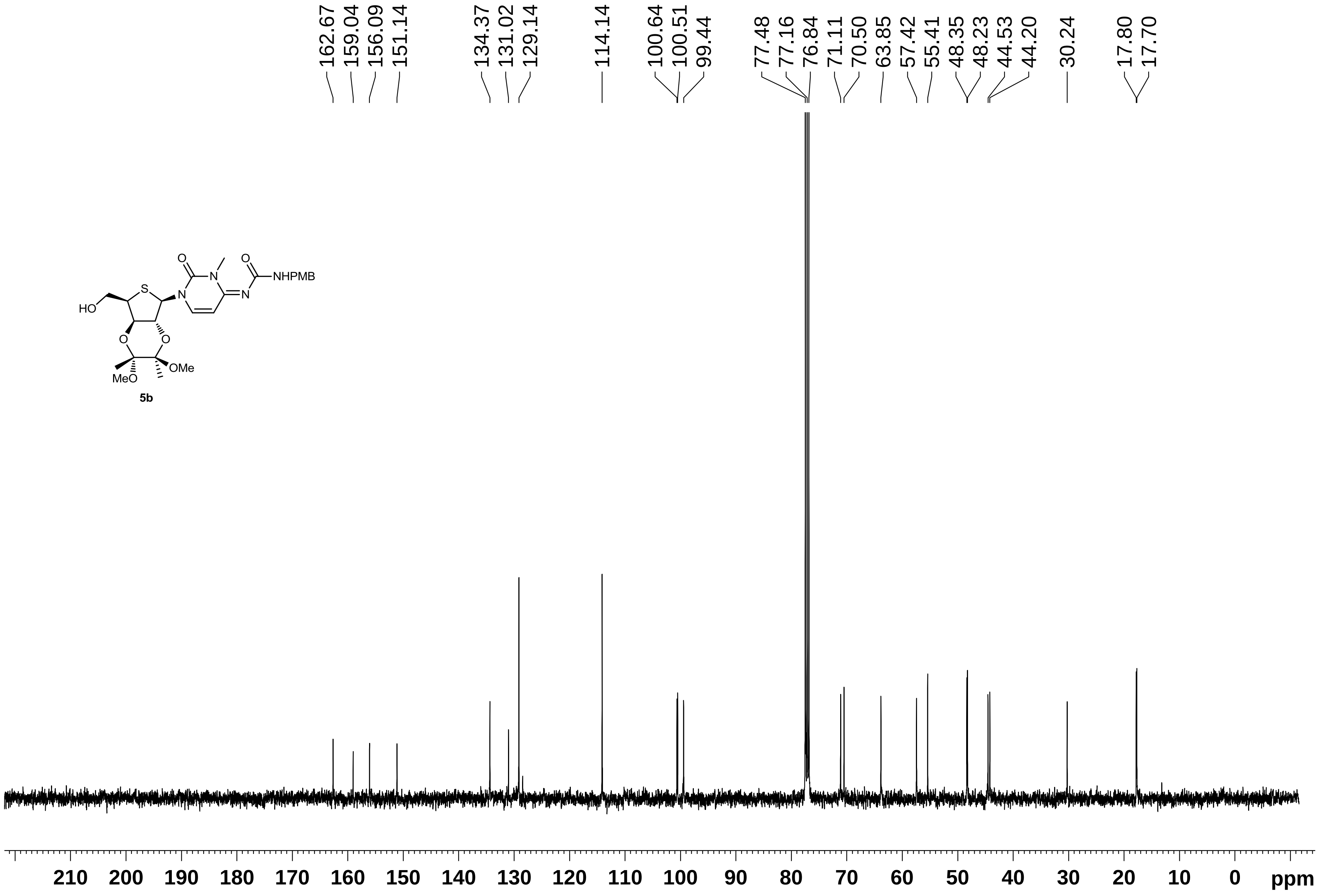

Supplementary Figure 18: ^{13}C NMR for compound **5b** ($CDCl_3$, 100 MHz).

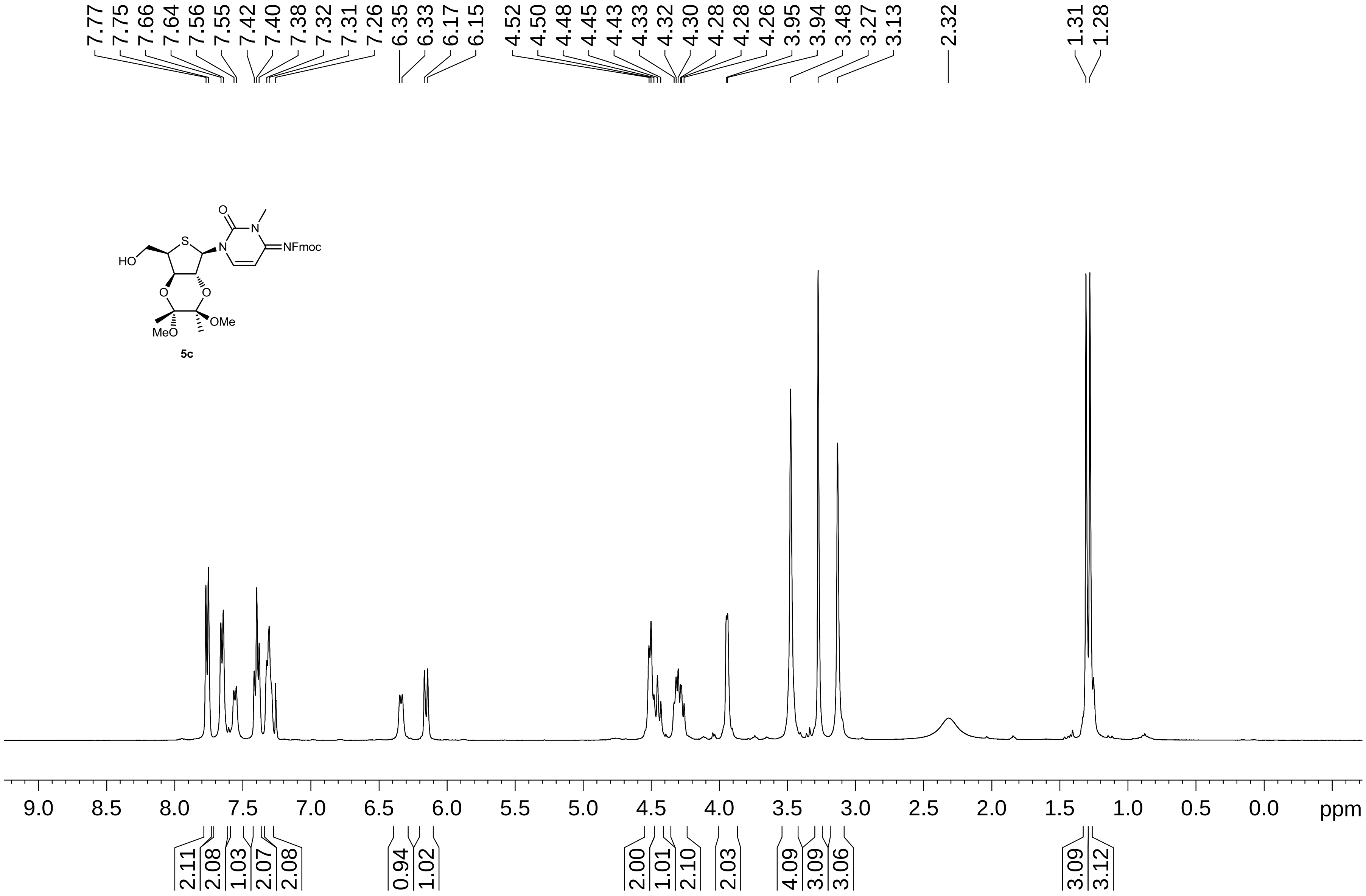

Supplementary Figure 19: ^{1}H NMR for compound **5c** ($CDCl_3$, 400 MHz).

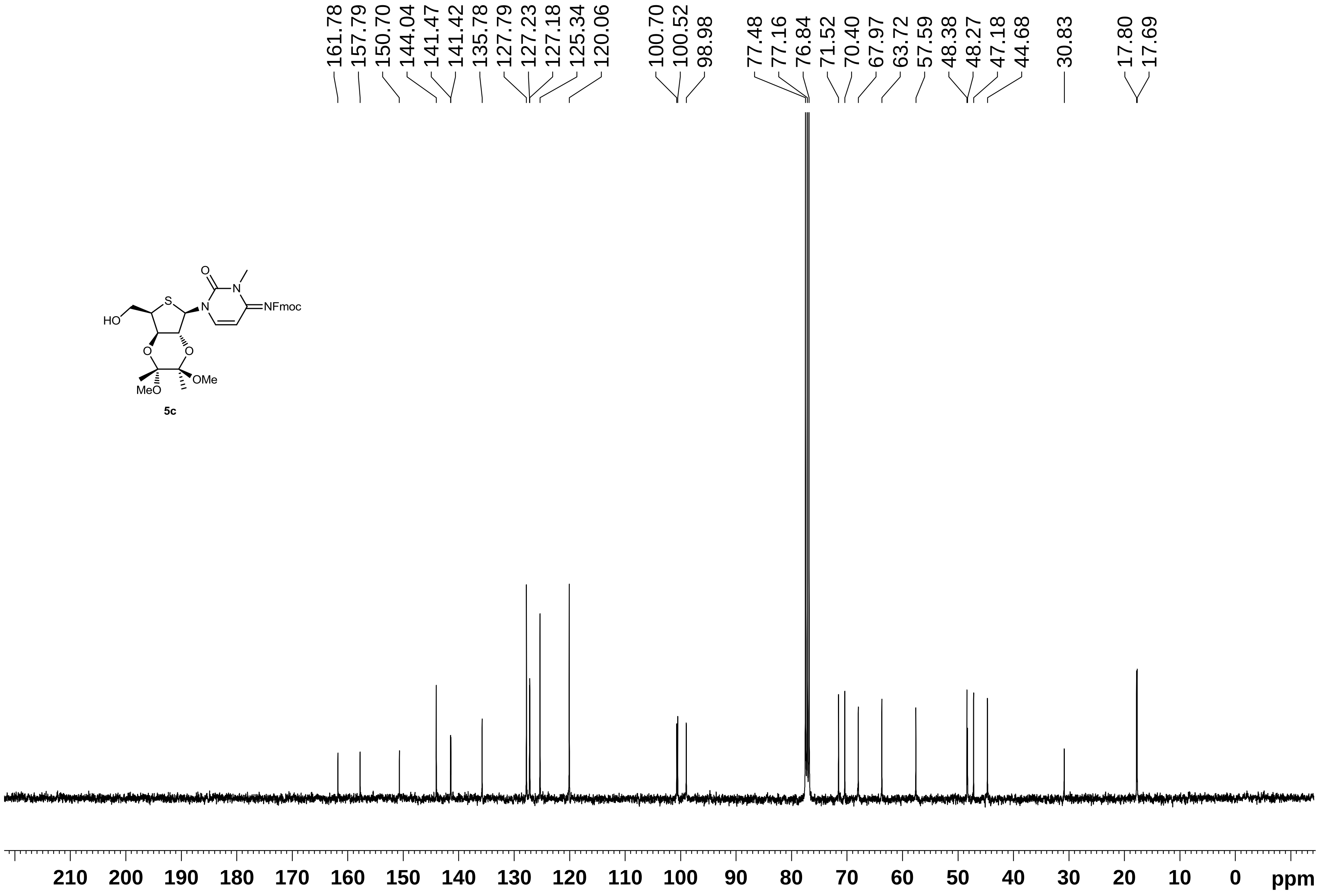

Supplementary Figure 20: ^{13}C NMR for compound **5c** ($CDCl_3$, 100 MHz).

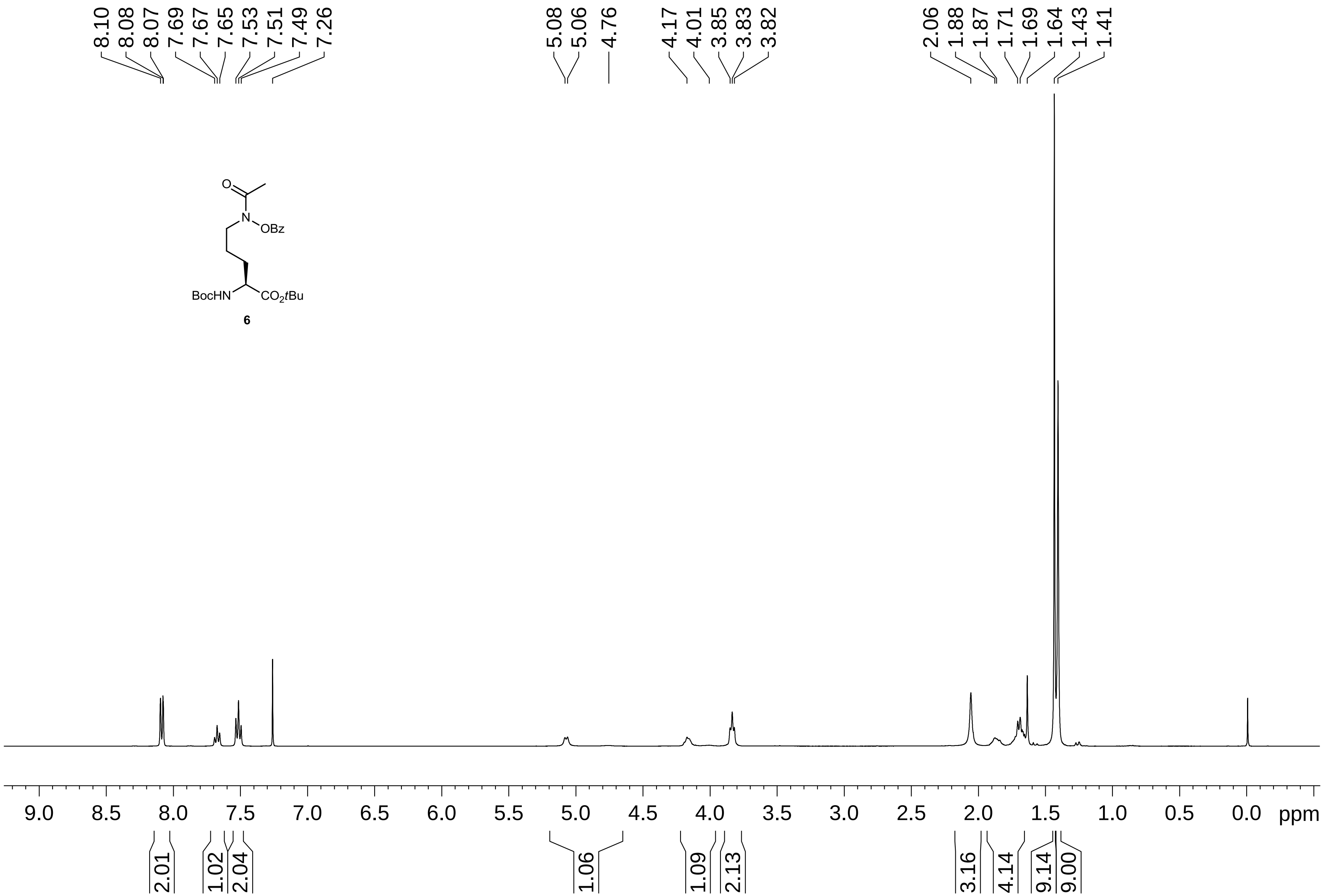

Supplementary Figure 21: ^{1}H NMR for compound **6** ($CDCl_3$, 400 MHz).

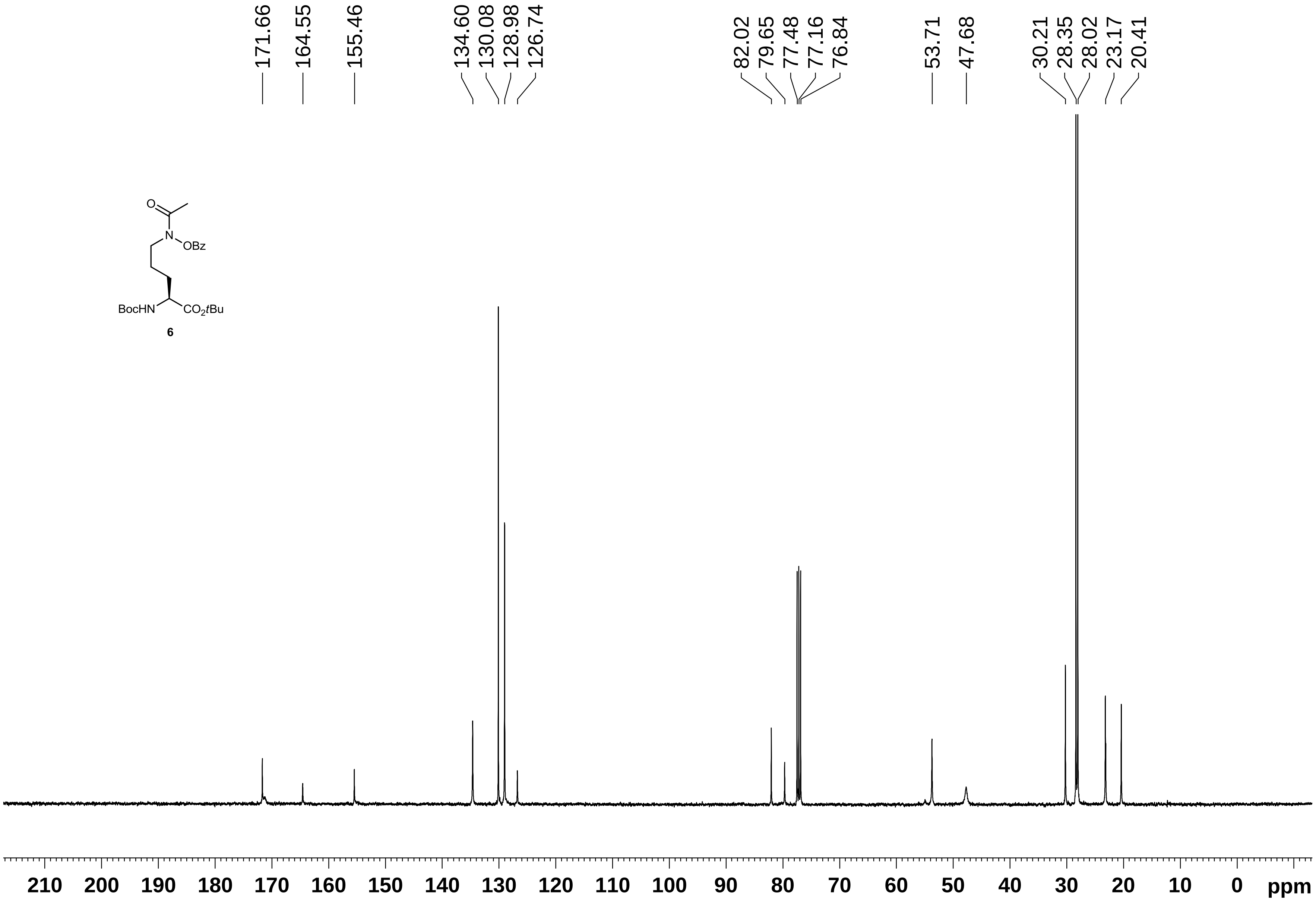

Supplementary Figure 22: ^{13}C NMR for compound **6** ($CDCl_3$, 100 MHz).

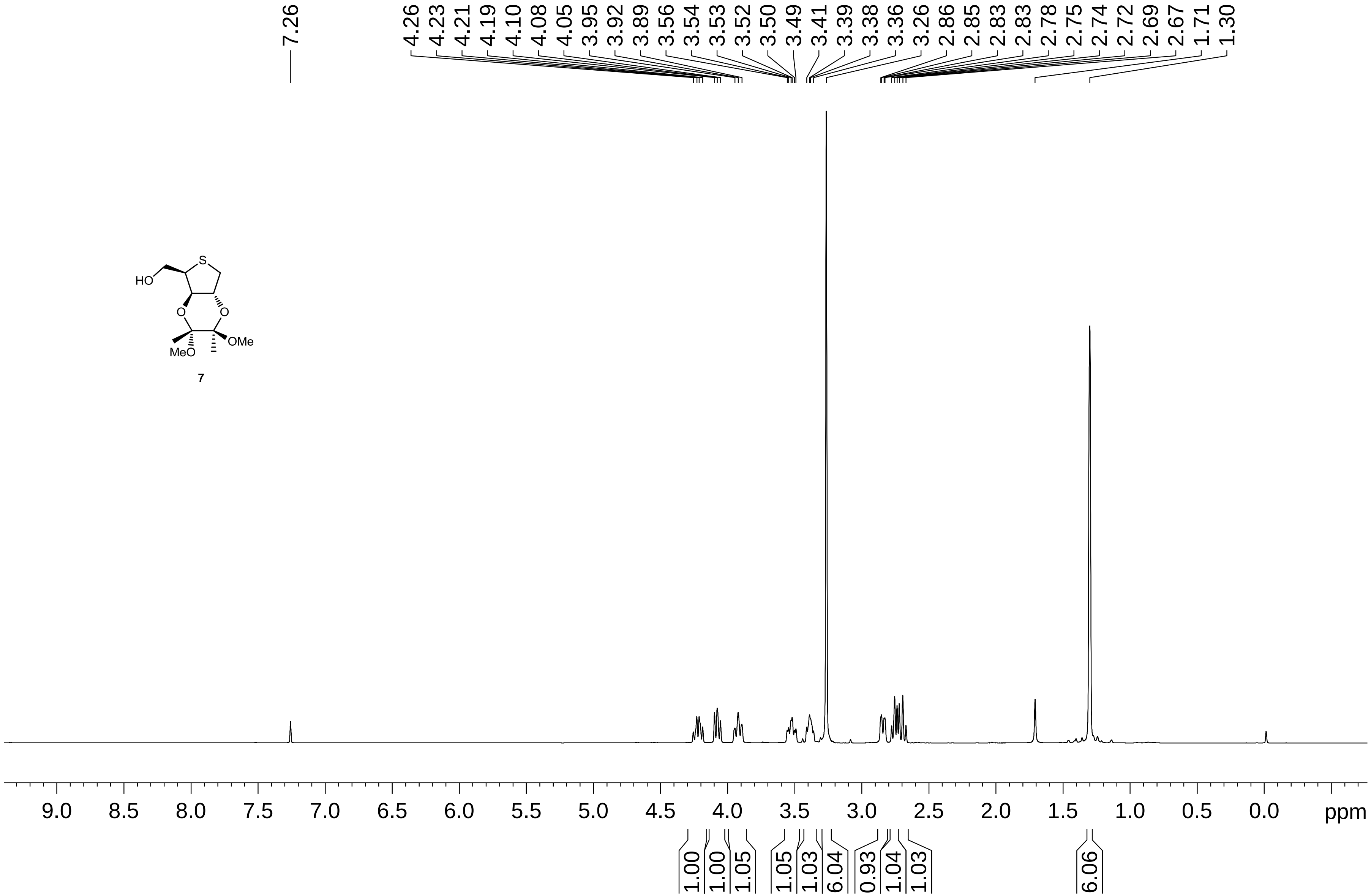

Supplementary Figure 23: ^{1}H NMR for compound **7** ($CDCl_3$, 400 MHz).

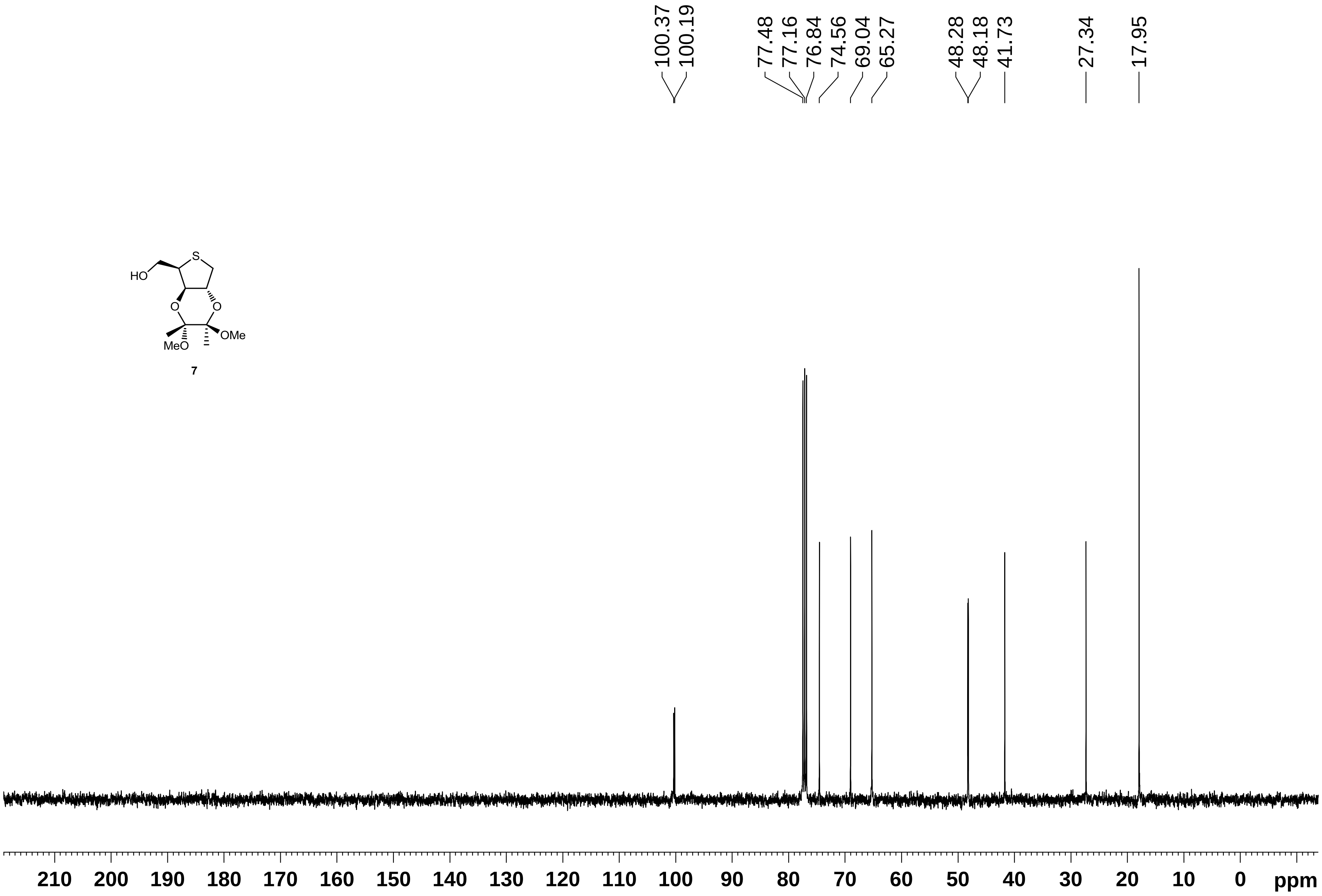

Supplementary Figure 24: ^{13}C NMR for compound **7** ($CDCl_3$, 100 MHz).

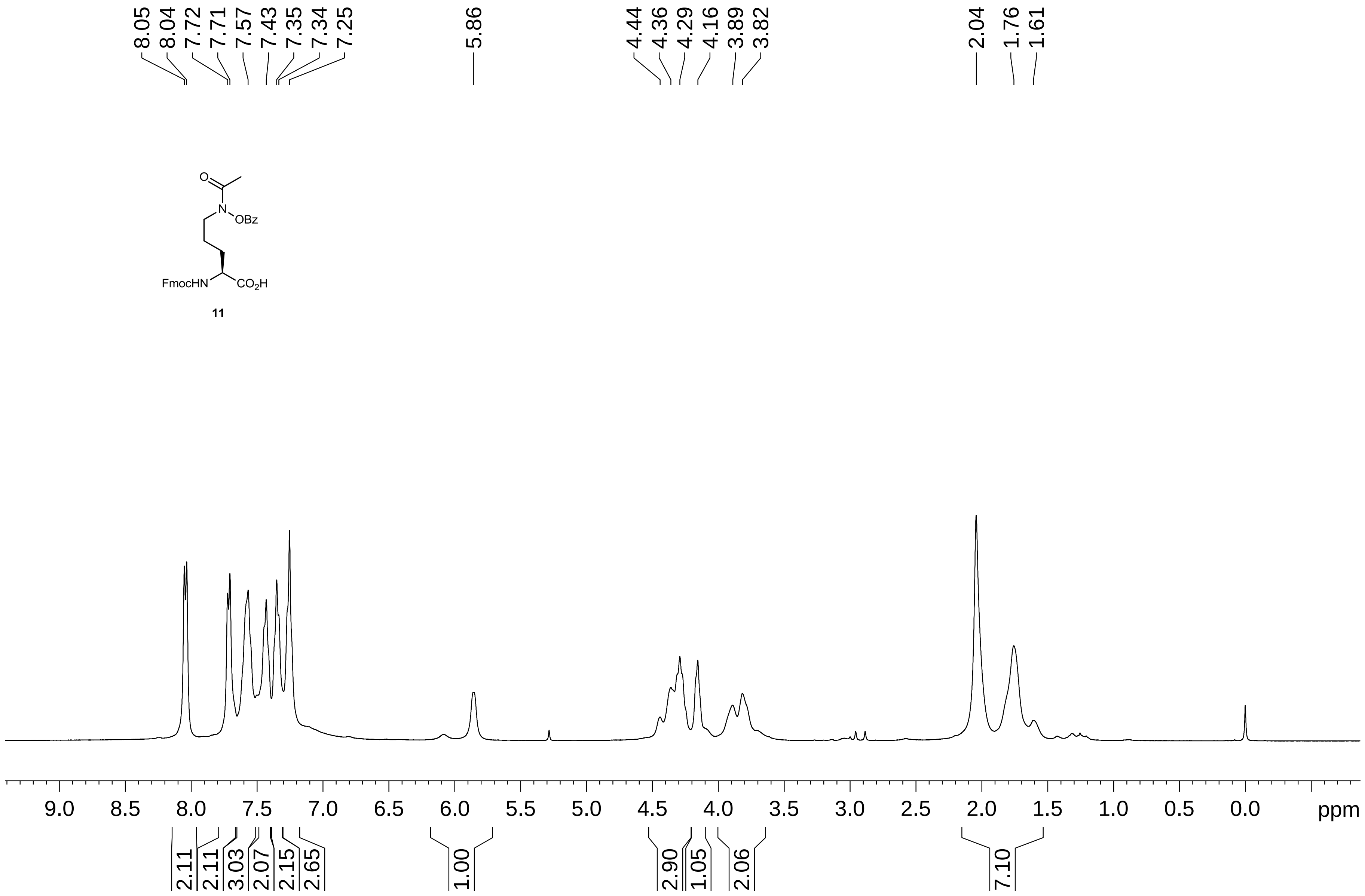

Supplementary Figure 25: ^{1}H NMR for compound **11** ($CDCl_3$, 400 MHz).

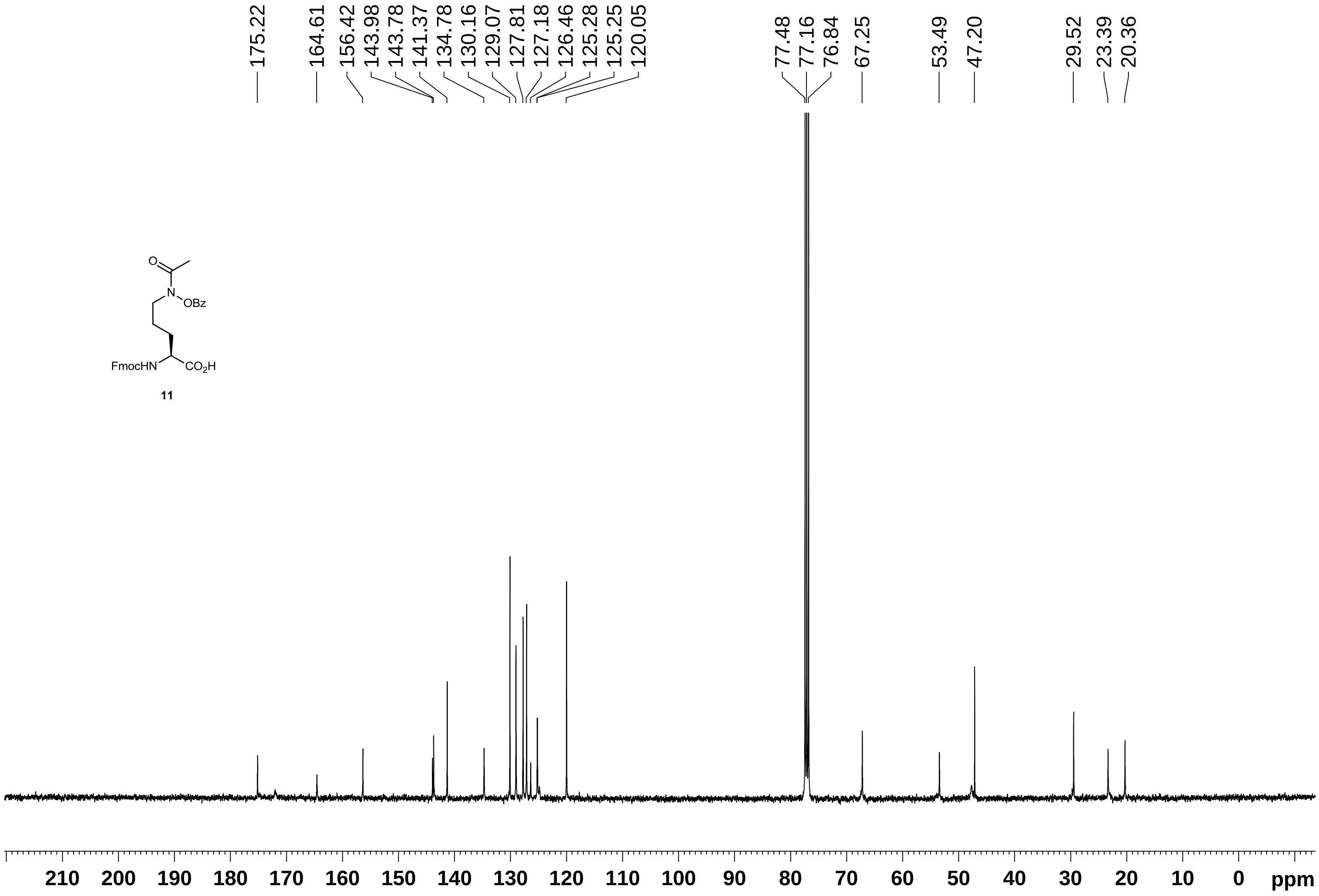

Supplementary Figure 26: ^{13}C NMR for compound **11** ($CDCl_3$, 100 MHz).

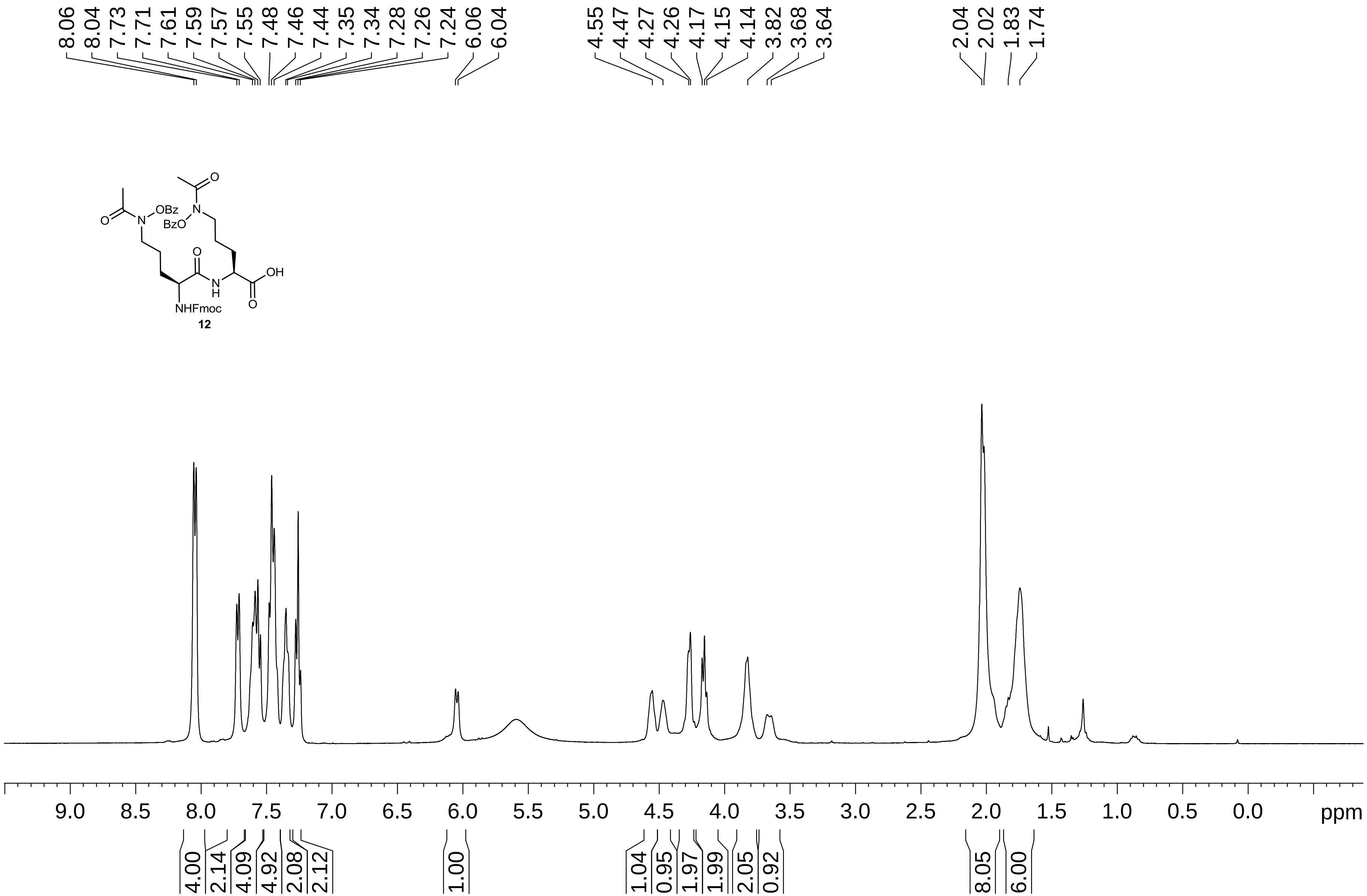

Supplementary Figure 27: ^{1}H NMR for compound **12** ($CDCl_3$, 400 MHz).

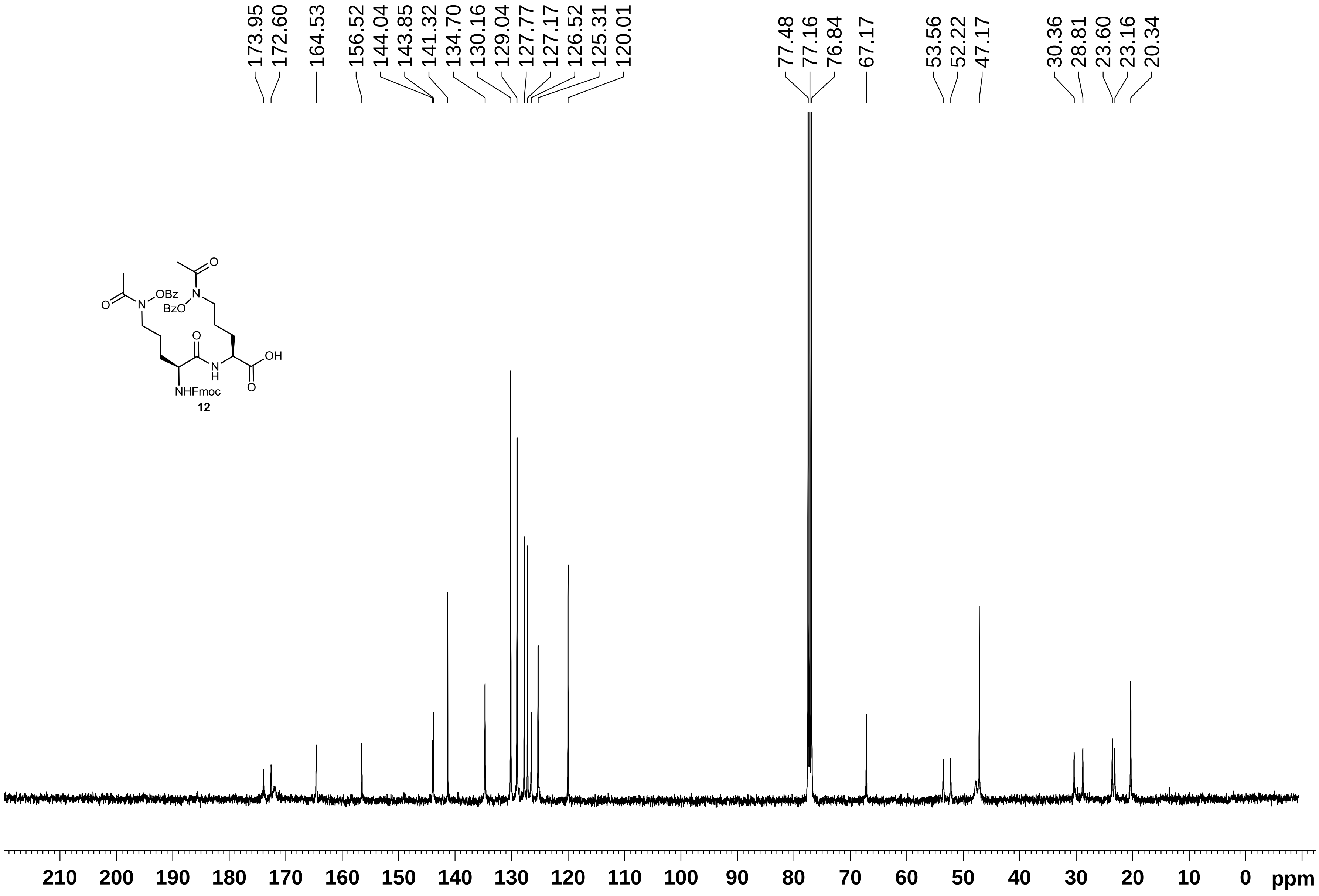

Supplementary Figure 28: ^{13}C NMR for compound **12** ($CDCl_3$, 100 MHz).

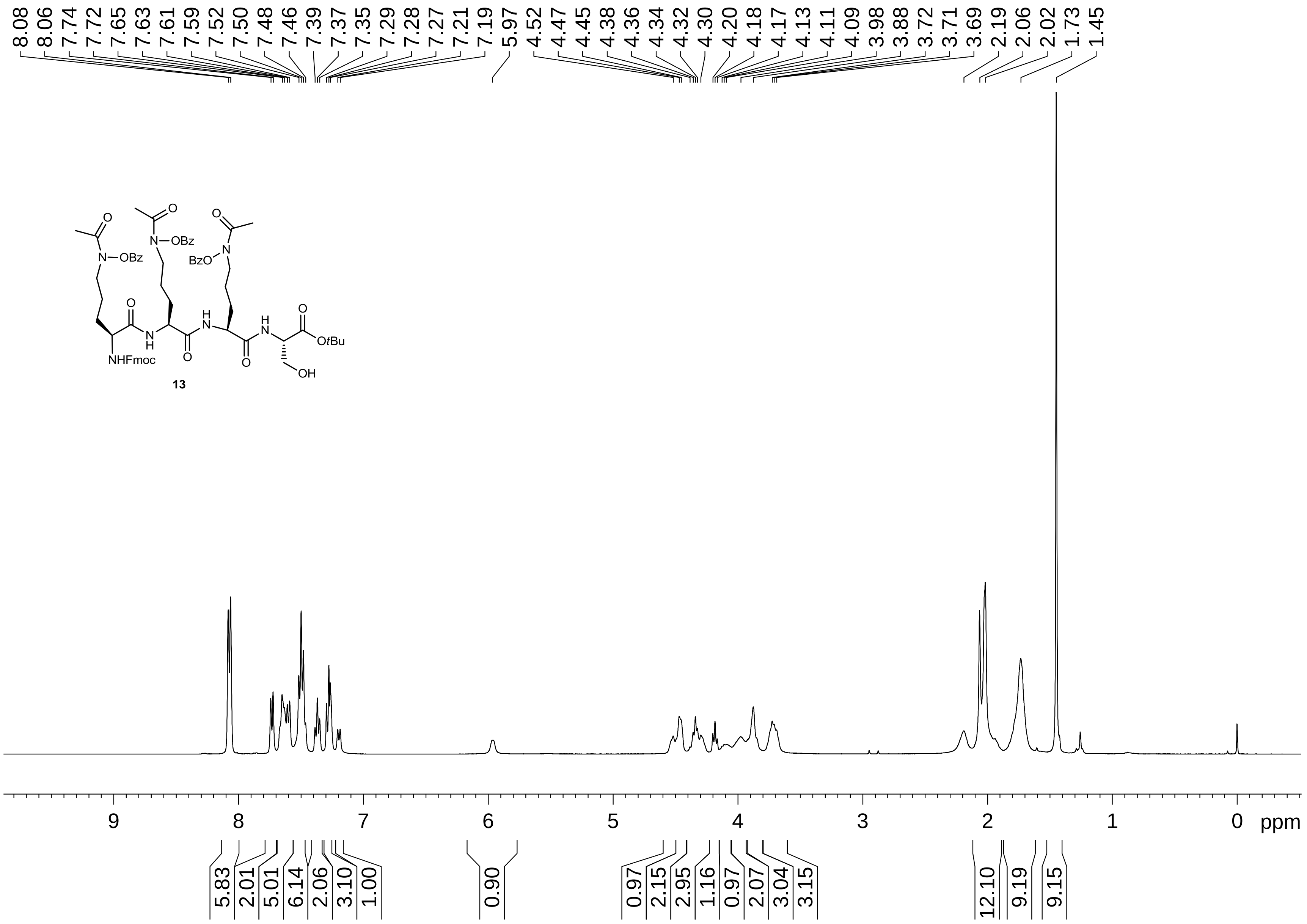

Supplementary Figure 29: ^{1}H NMR for compound **13** ($CDCl_3$, 400 MHz).

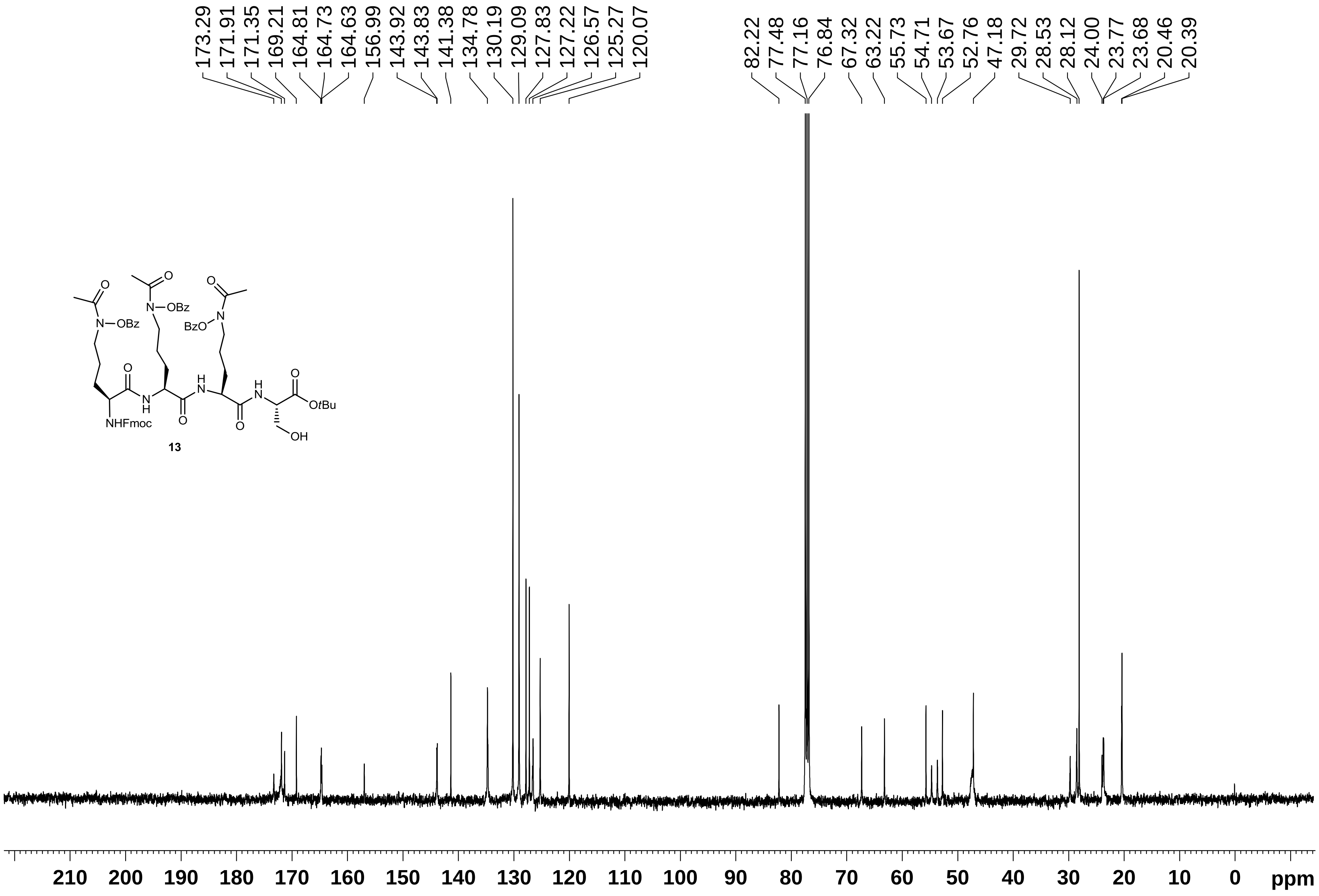

Supplementary Figure 30: ^{13}C NMR for compound **13** ($CDCl_3$, 100 MHz).

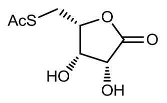

14

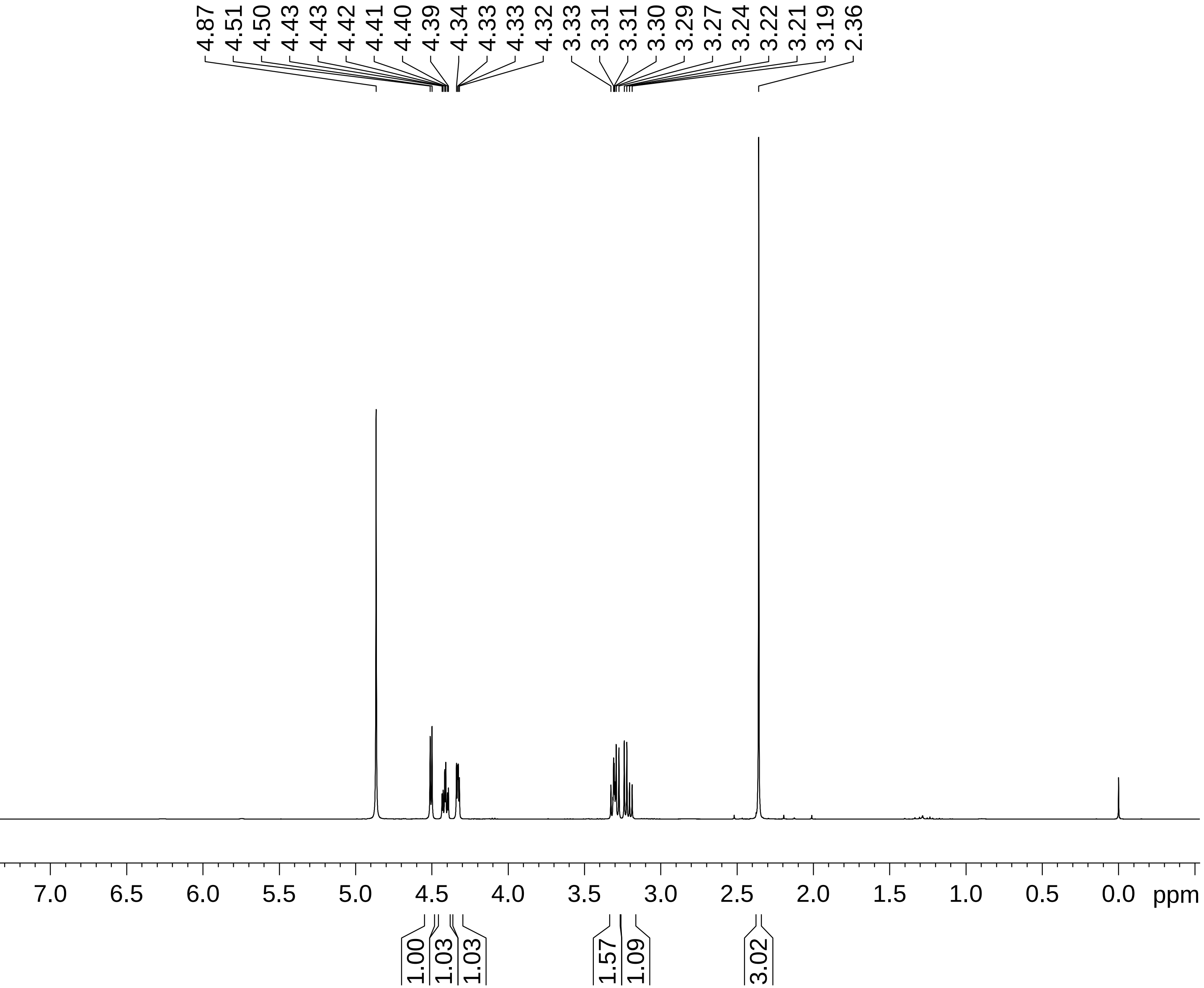

Supplementary Figure 31: ^{1}H NMR for compound **14** (CD_3OD, 400 MHz).

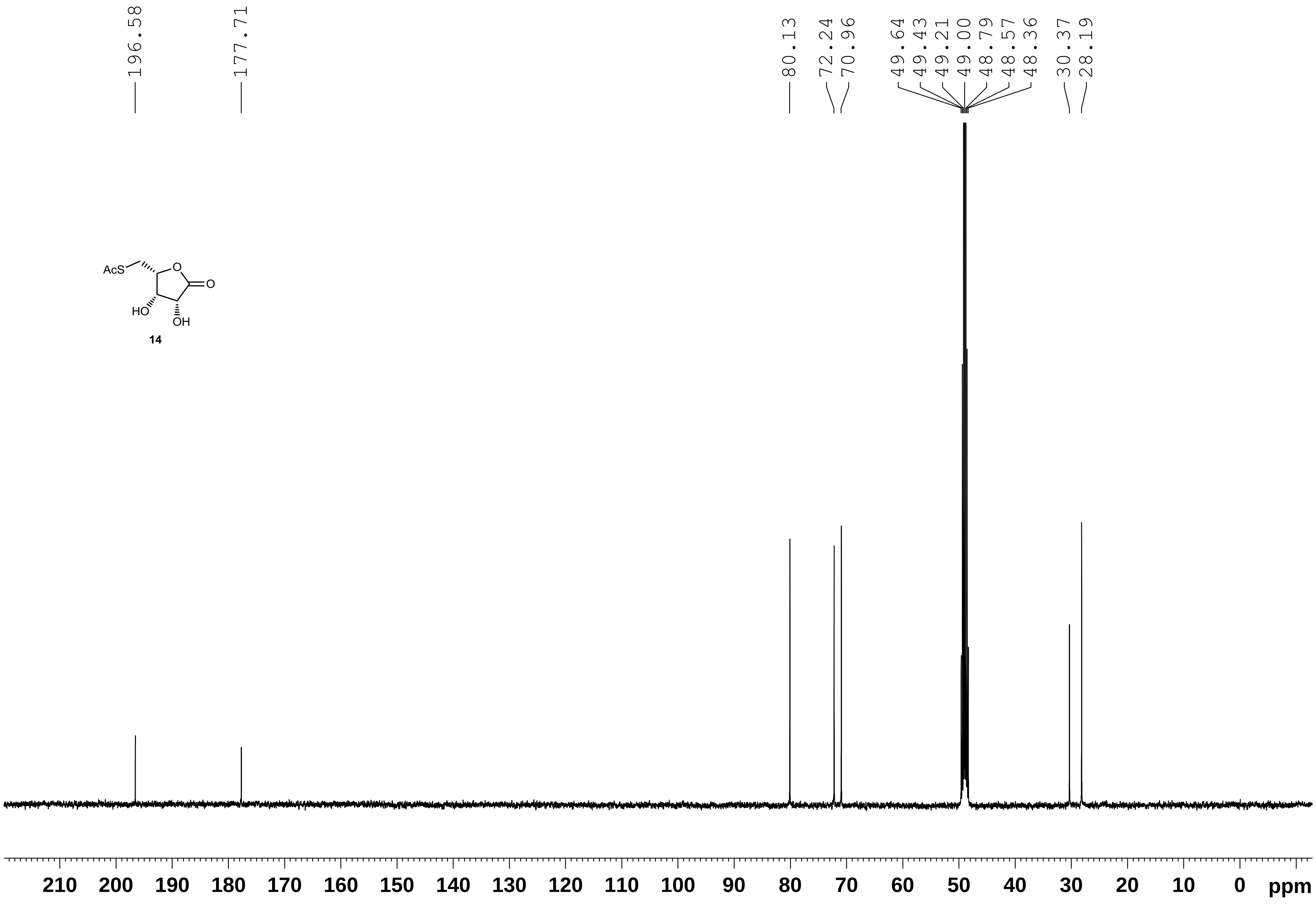

Supplementary Figure 32: ^{13}C NMR for compound **14** (CD_3OD, 100 MHz).

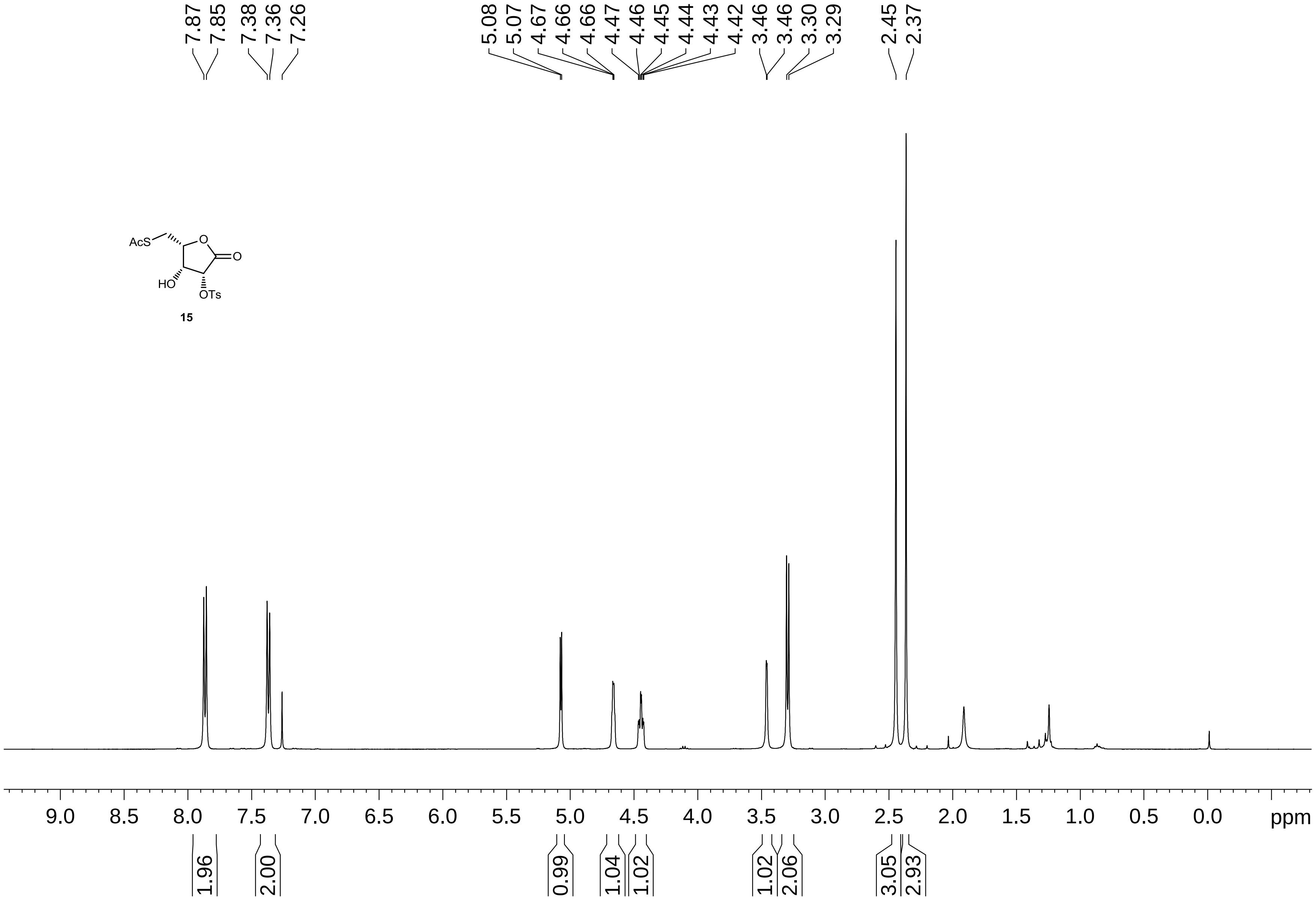

Supplementary Figure 33: ^{1}H NMR for compound **15** ($CDCl_3$, 400 MHz).

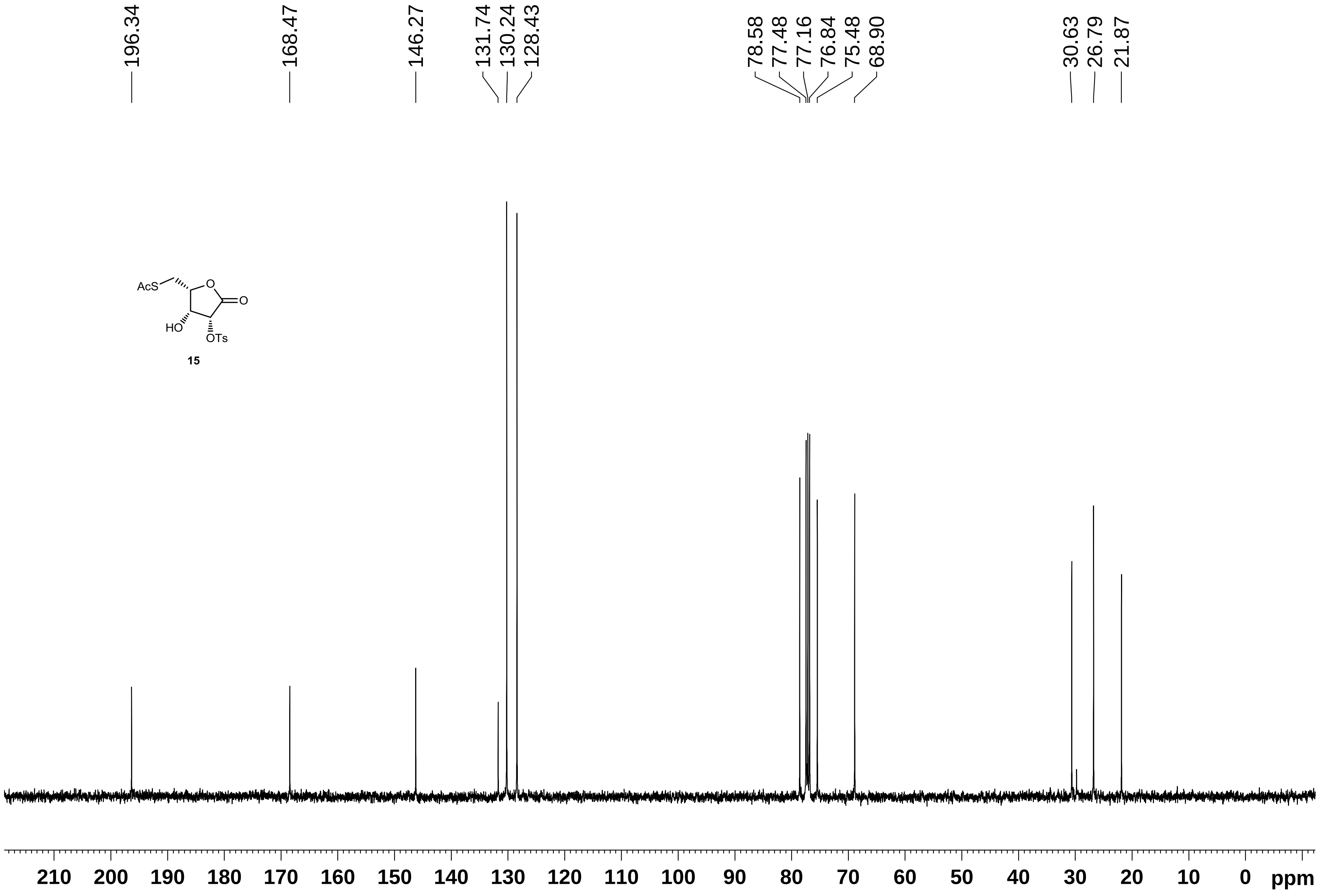

Supplementary Figure 34: ^{13}C NMR for compound **15** ($CDCl_3$, 100 MHz).

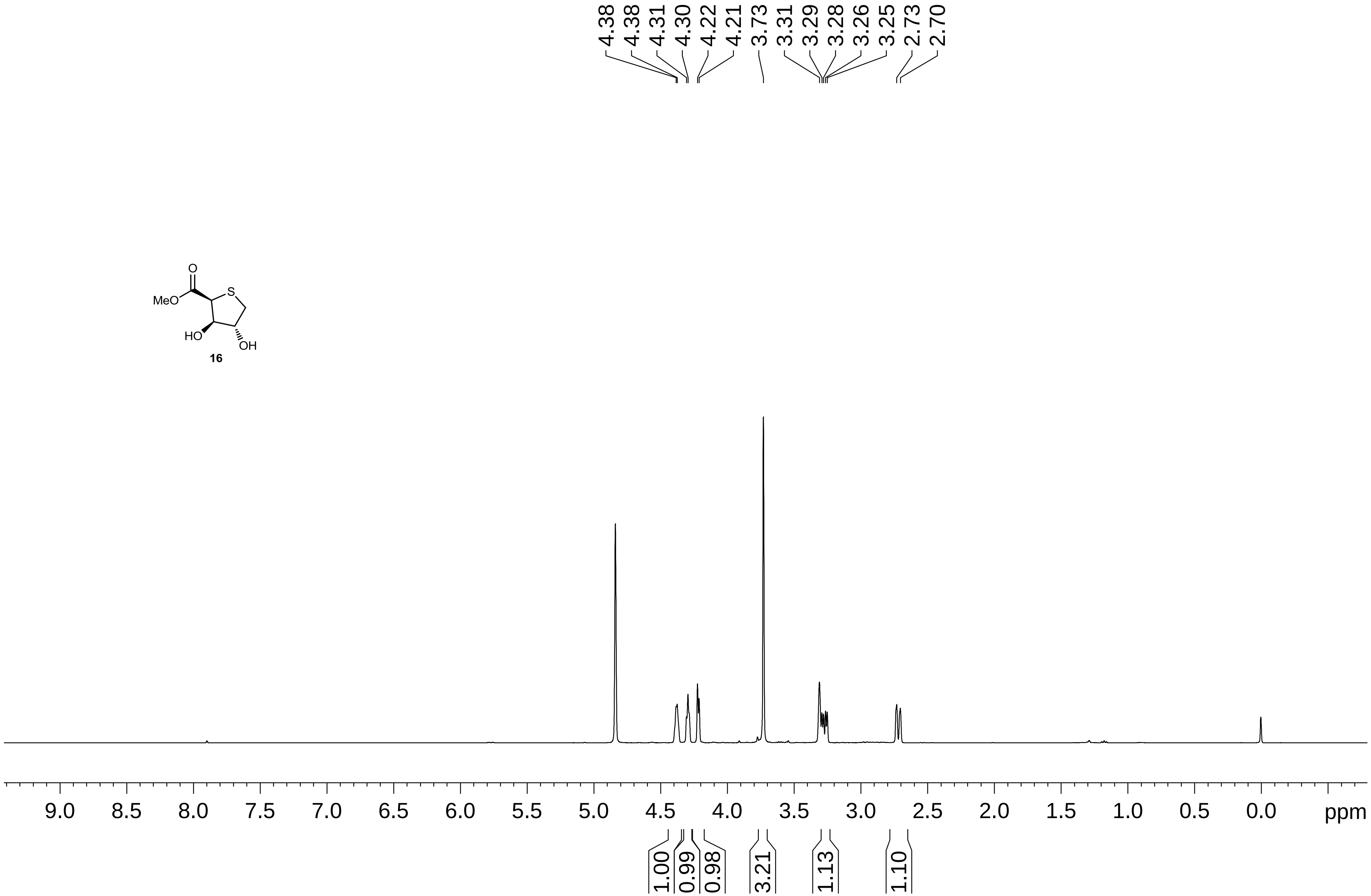

Supplementary Figure 35: ^{1}H NMR for compound **16** (CD_3OD, 400 MHz).

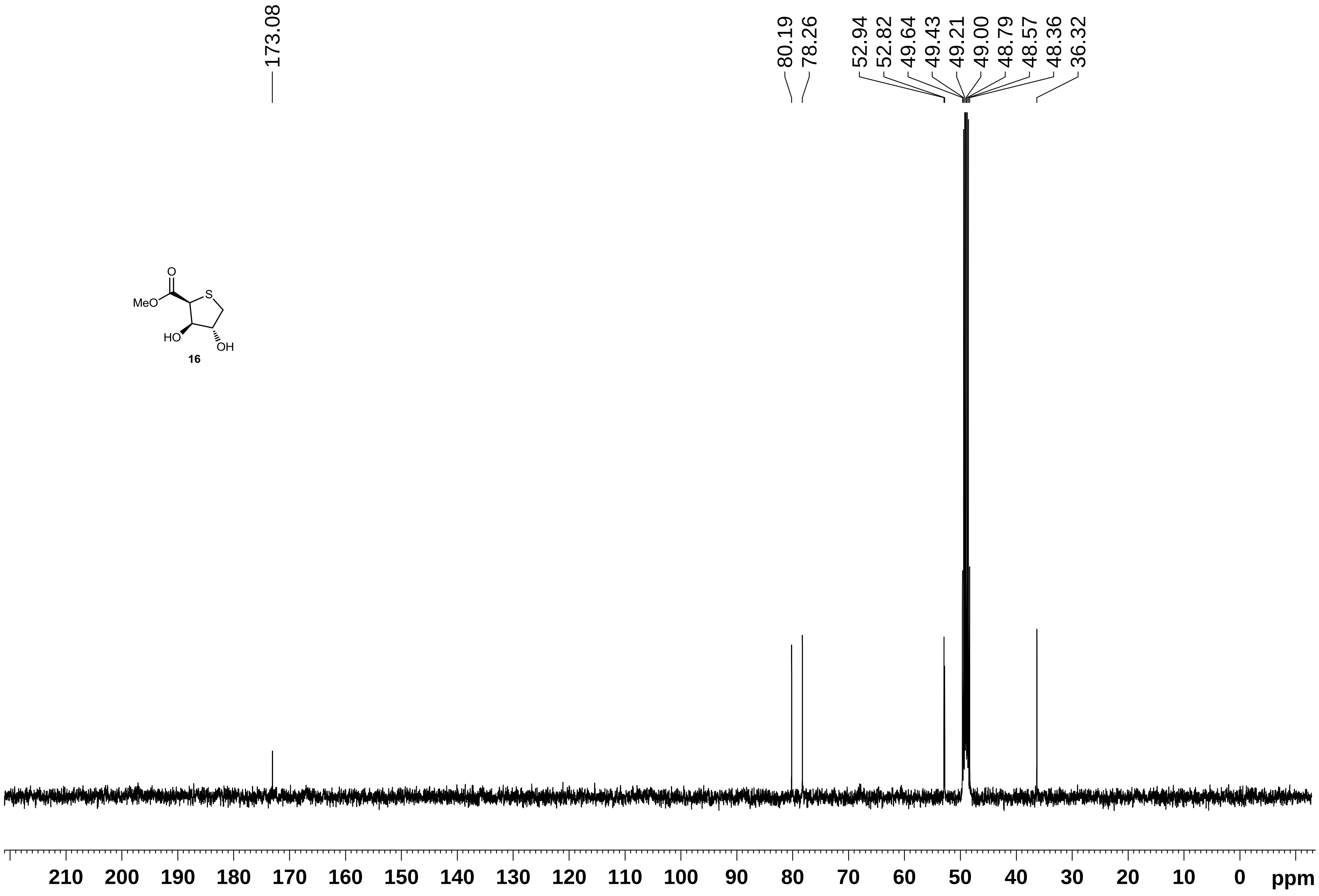

Supplementary Figure 36: ^{13}C NMR for compound **16** (CD_3OD, 100 MHz).

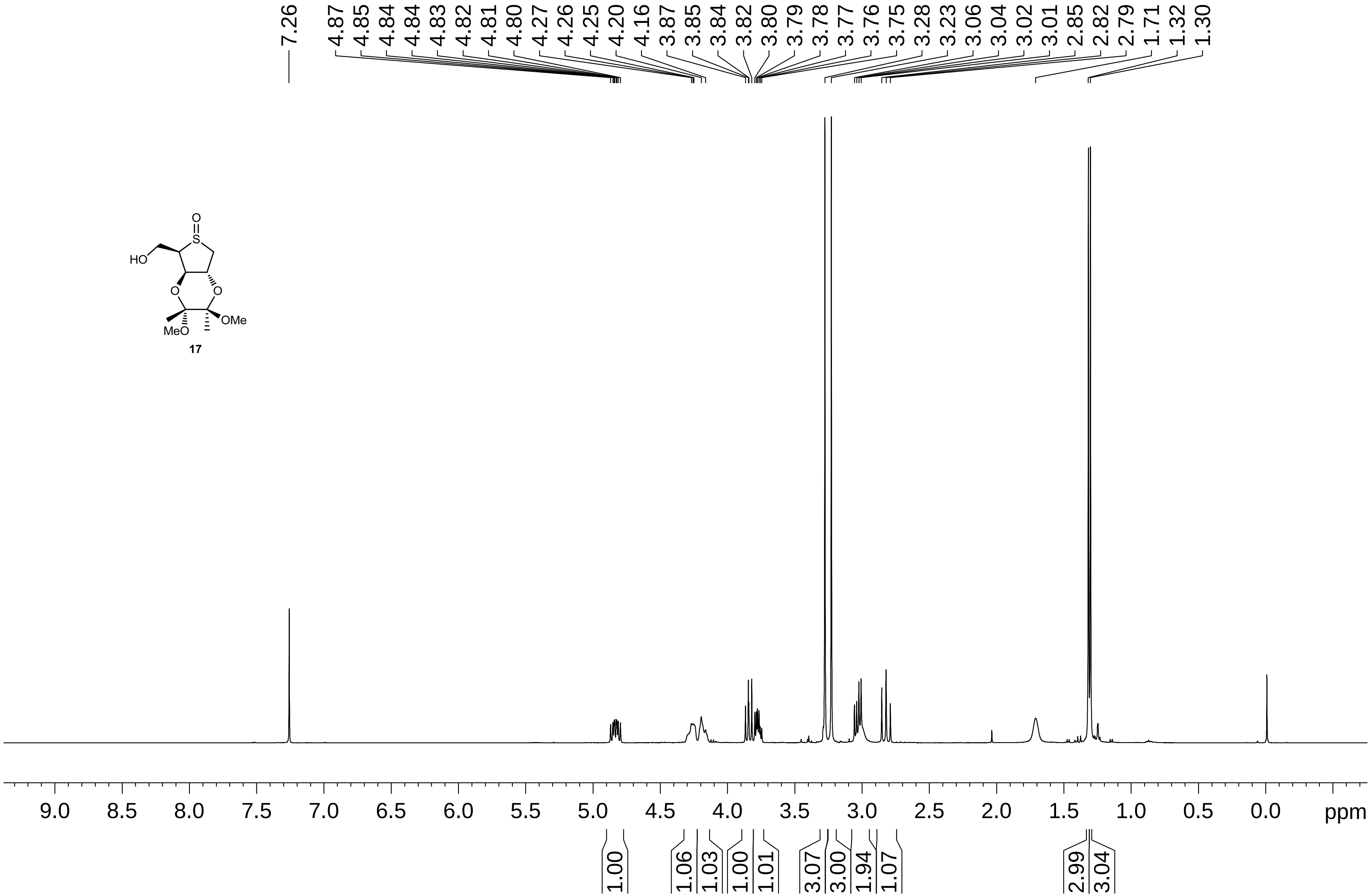

Supplementary Figure 37: ^{1}H NMR for compound **17** ($CDCl_3$, 400 MHz).

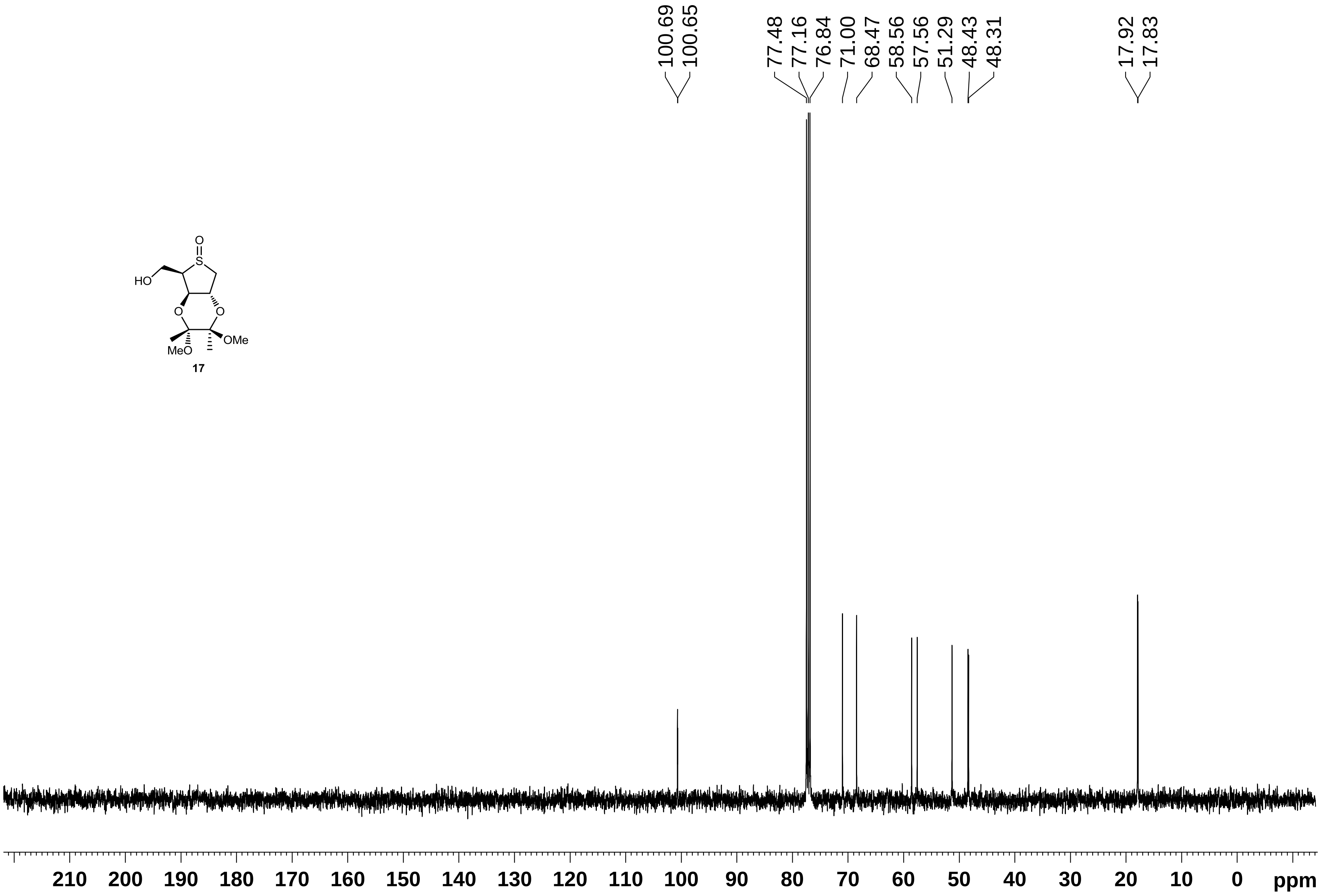

Supplementary Figure 38: ^{13}C NMR for compound **17** ($CDCl_3$, 100 MHz).

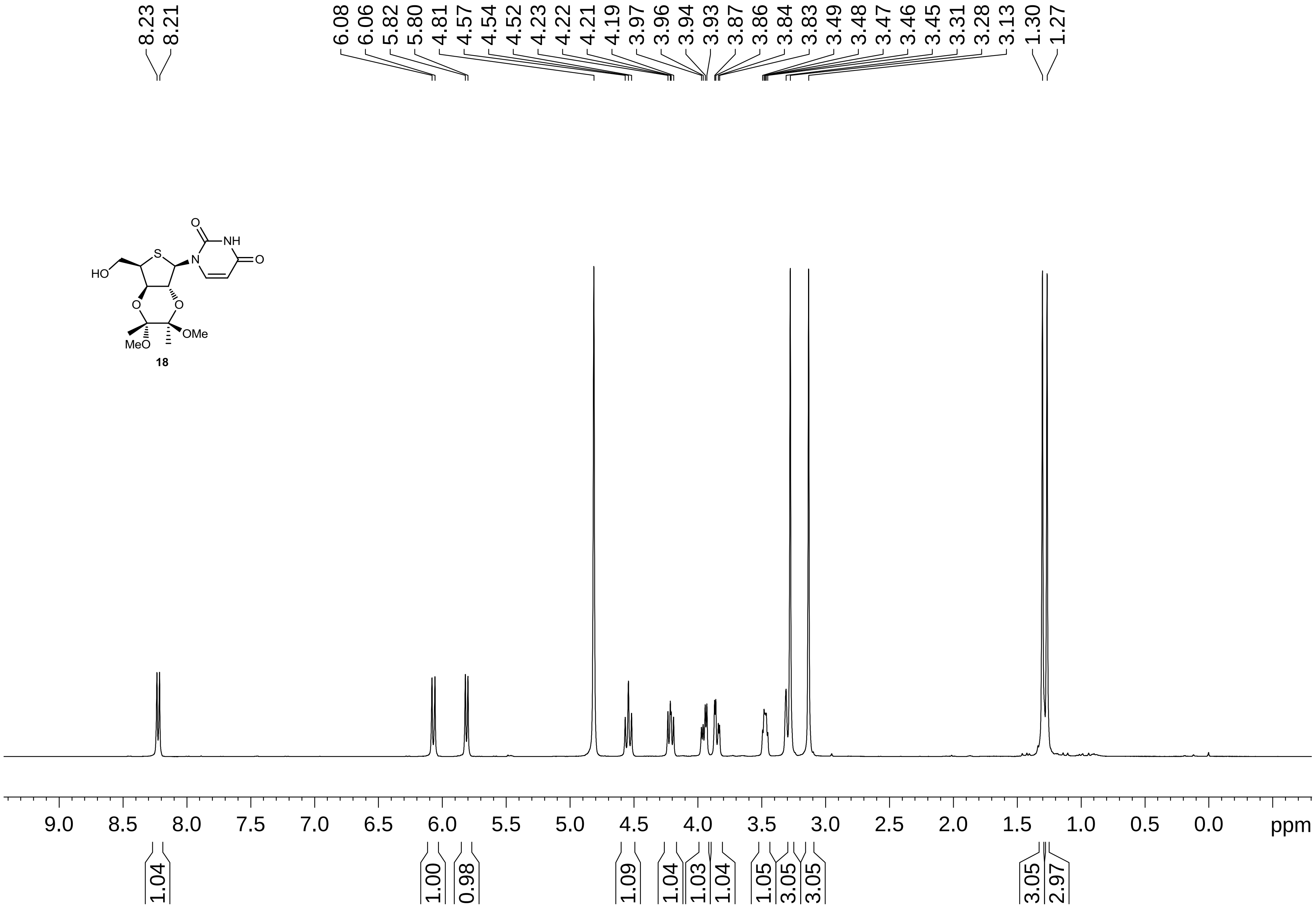

Supplementary Figure 39: ^{1}H NMR for compound **18** (CD_3OD, 400 MHz).

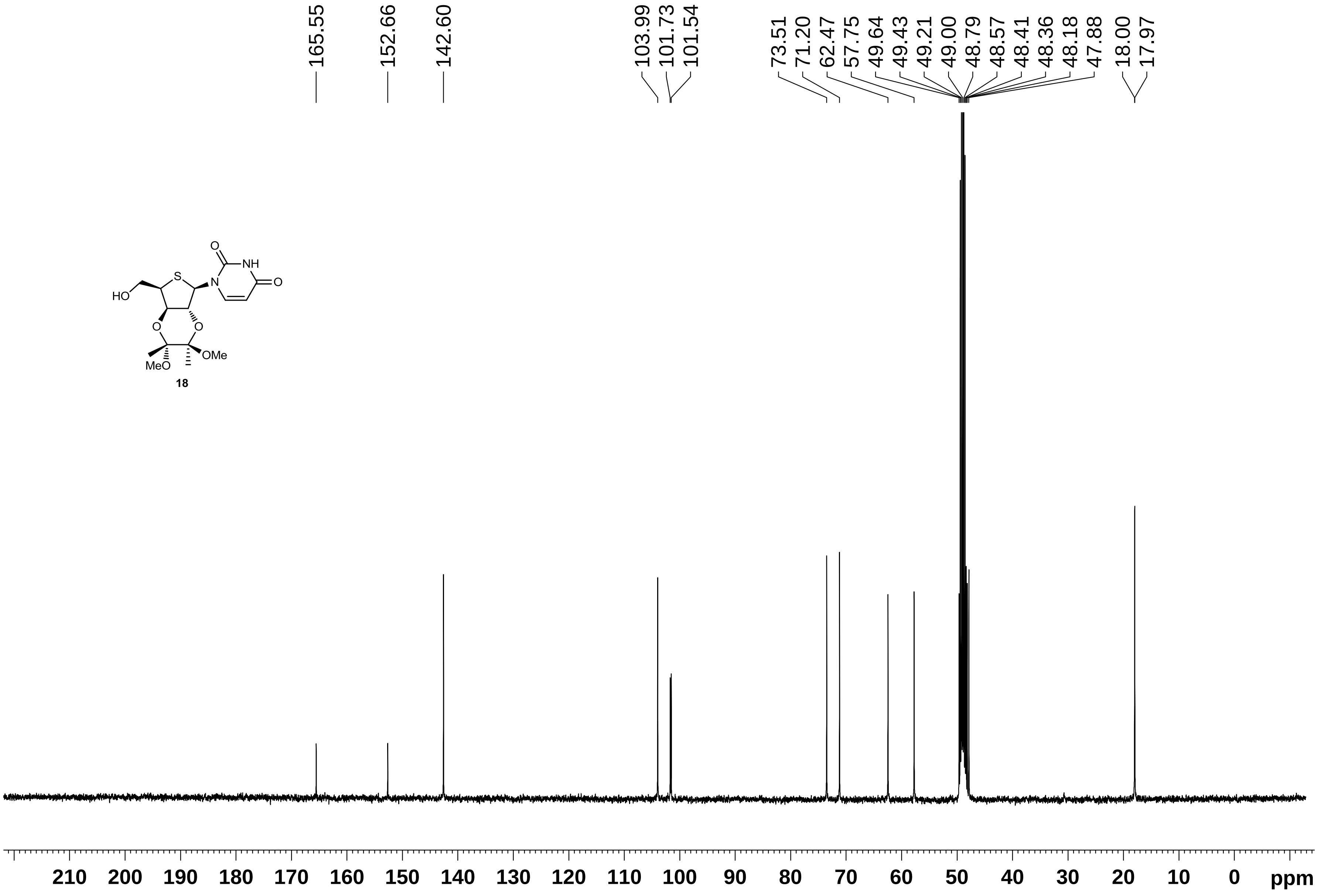

Supplementary Figure 40: ^{13}C NMR for compound **18** (CD_3OD, 100 MHz).

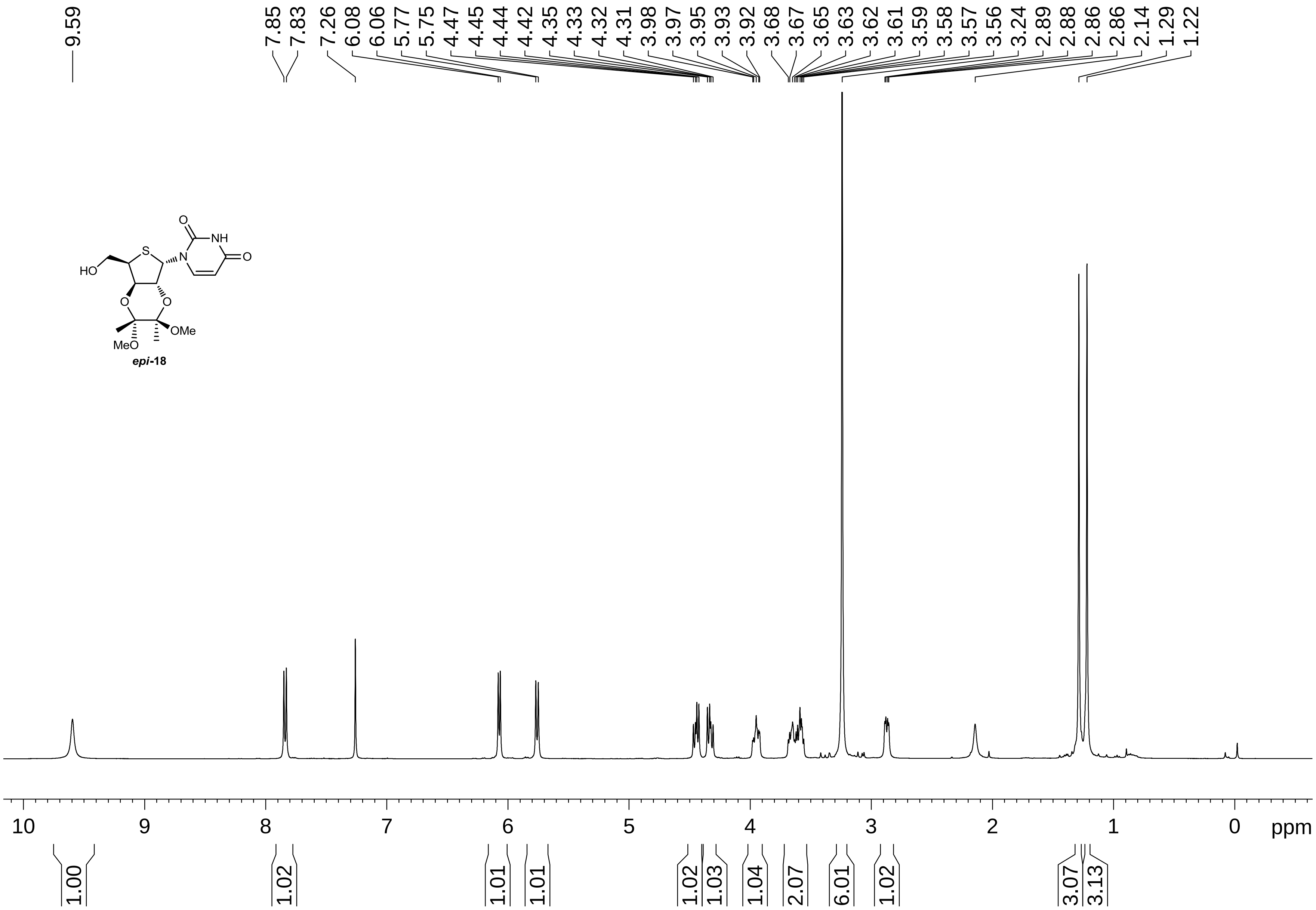

Supplementary Figure 41: ^{1}H NMR for compound ***epi*-18** ($CDCl_3$, 400 MHz).

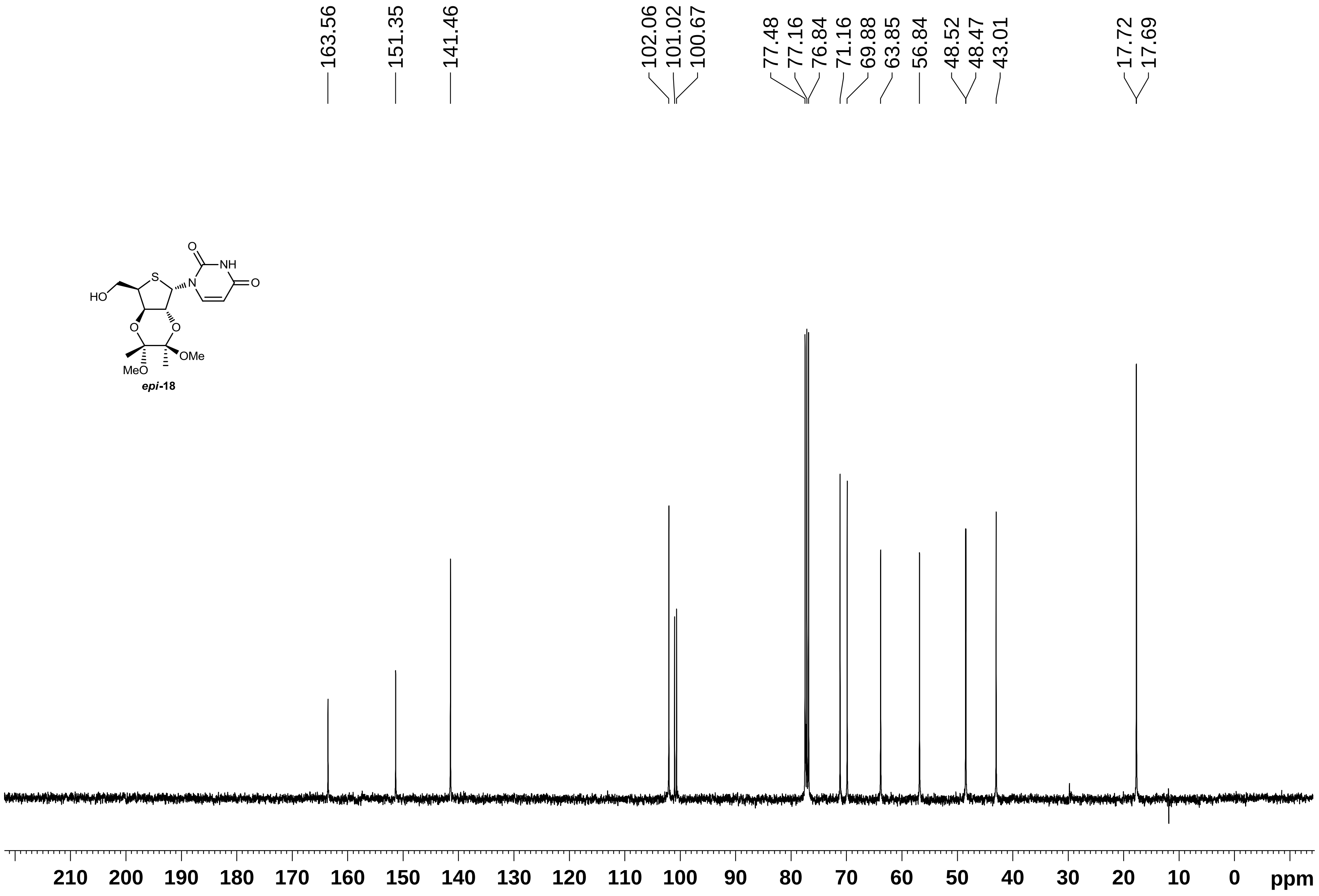

Supplementary Figure 42: ^{13}C NMR for compound ***epi*-18** ($CDCl_3$, 100 MHz).

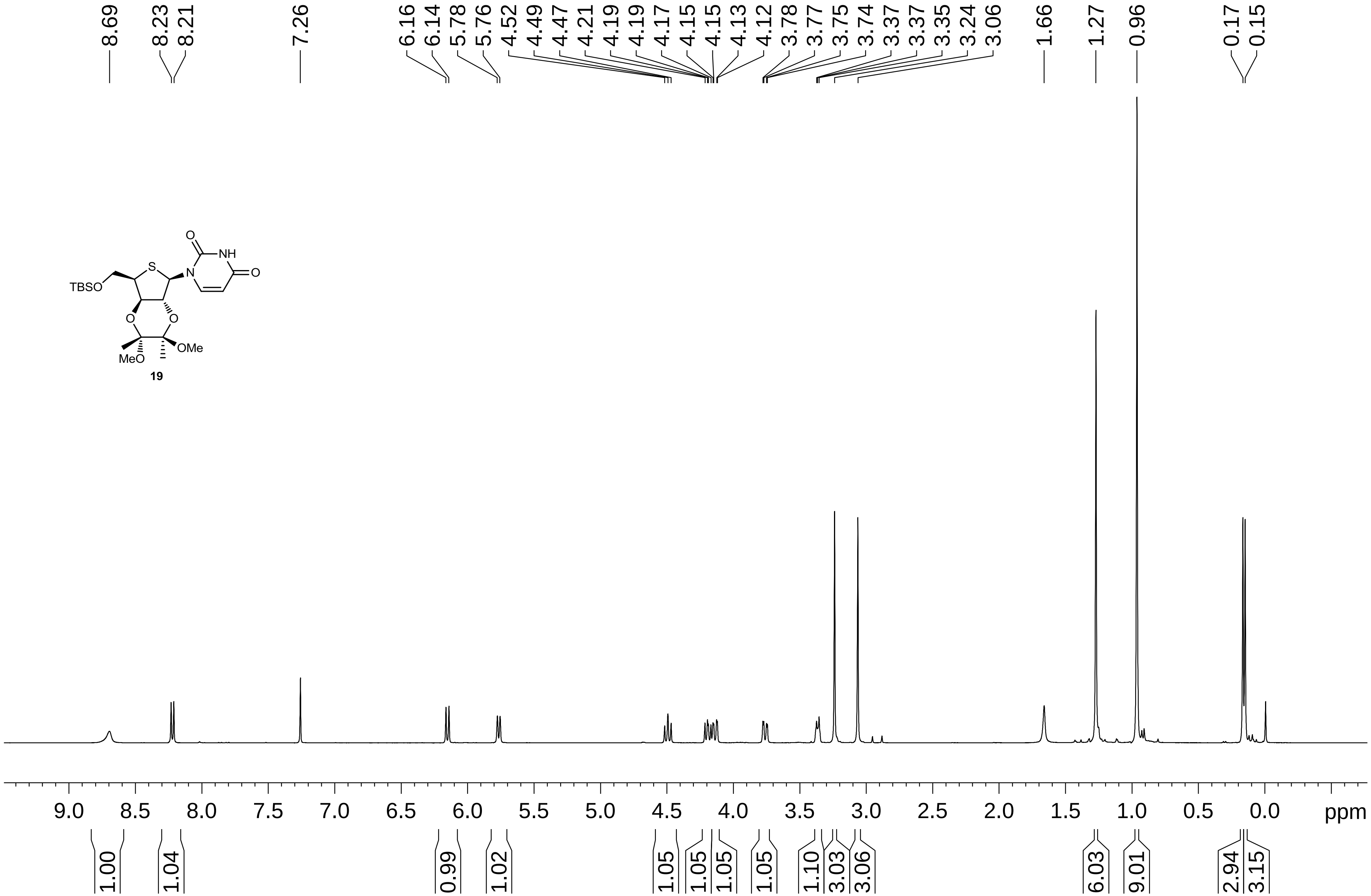

Supplementary Figure 43: ^{1}H NMR for compound **19** ($CDCl_3$, 400 MHz).

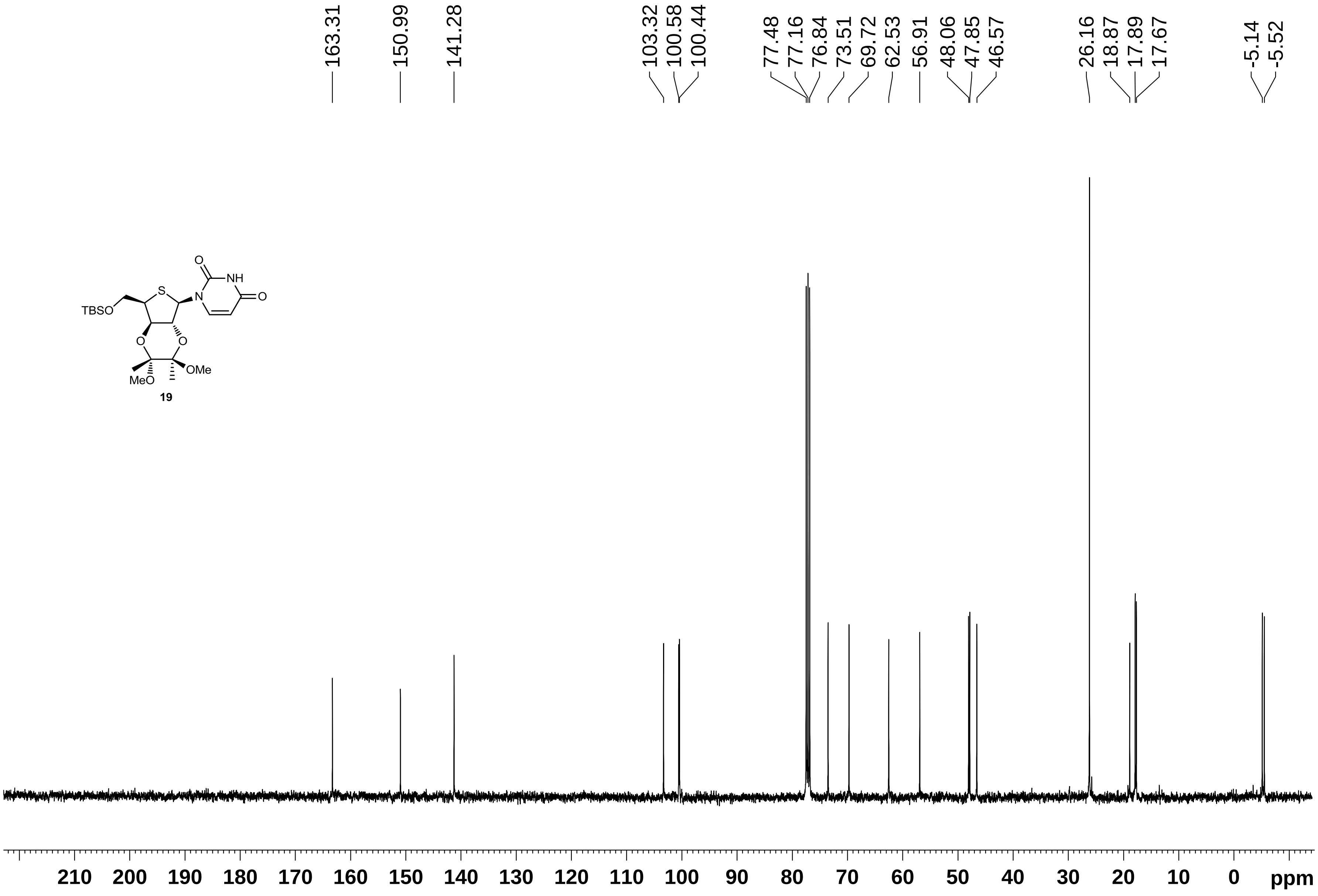

Supplementary Figure 44: ^{13}C NMR for compound **19** ($CDCl_3$, 100 MHz).

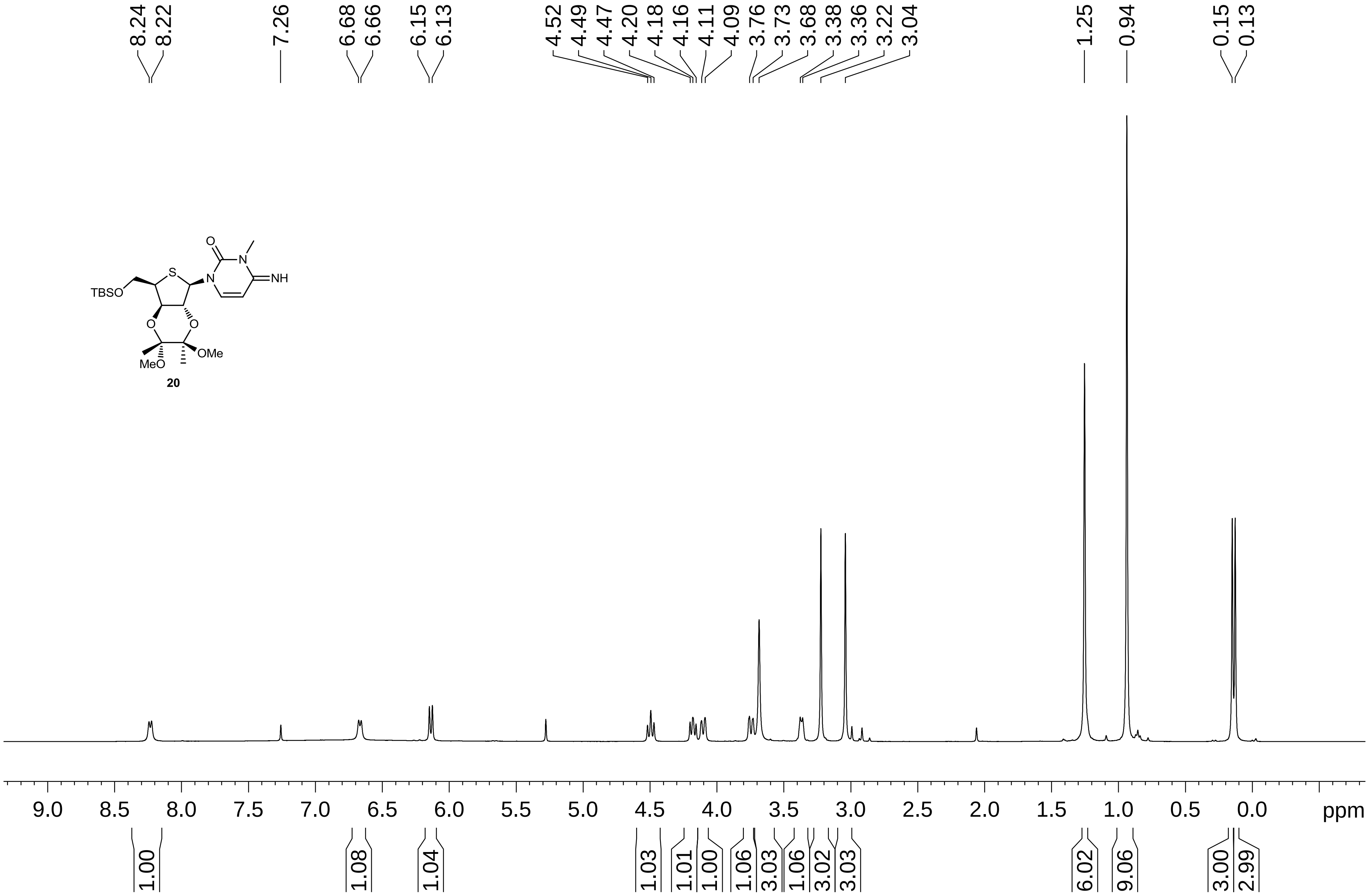

Supplementary Figure 45: ^{1}H NMR for compound **20** ($CDCl_3$, 400 MHz).

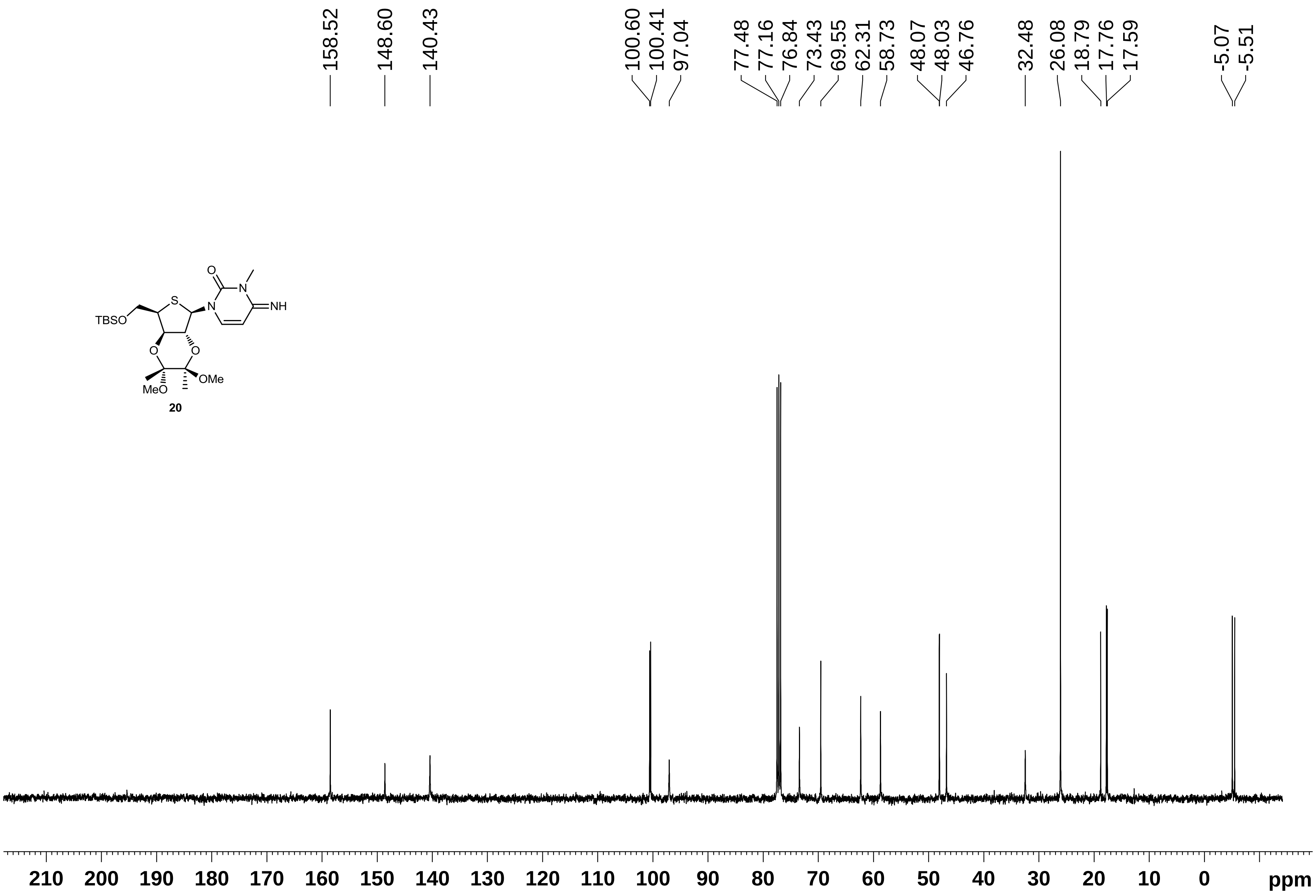

Supplementary Figure 46: ^{13}C NMR for compound **20** ($CDCl_3$, 100 MHz).

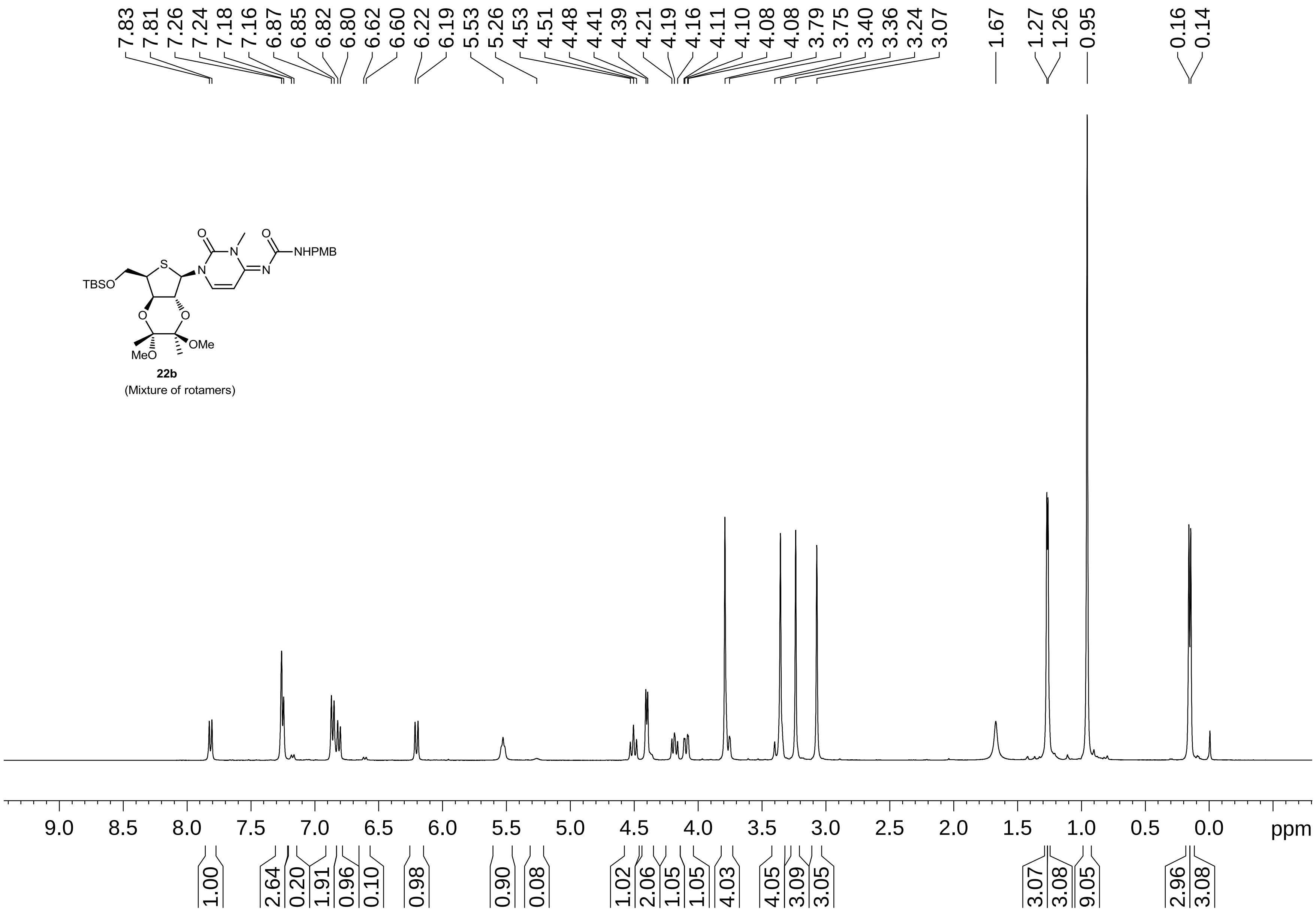

Supplementary Figure 47: ^{1}H NMR for compound **22b** ($CDCl_3$, 400 MHz).

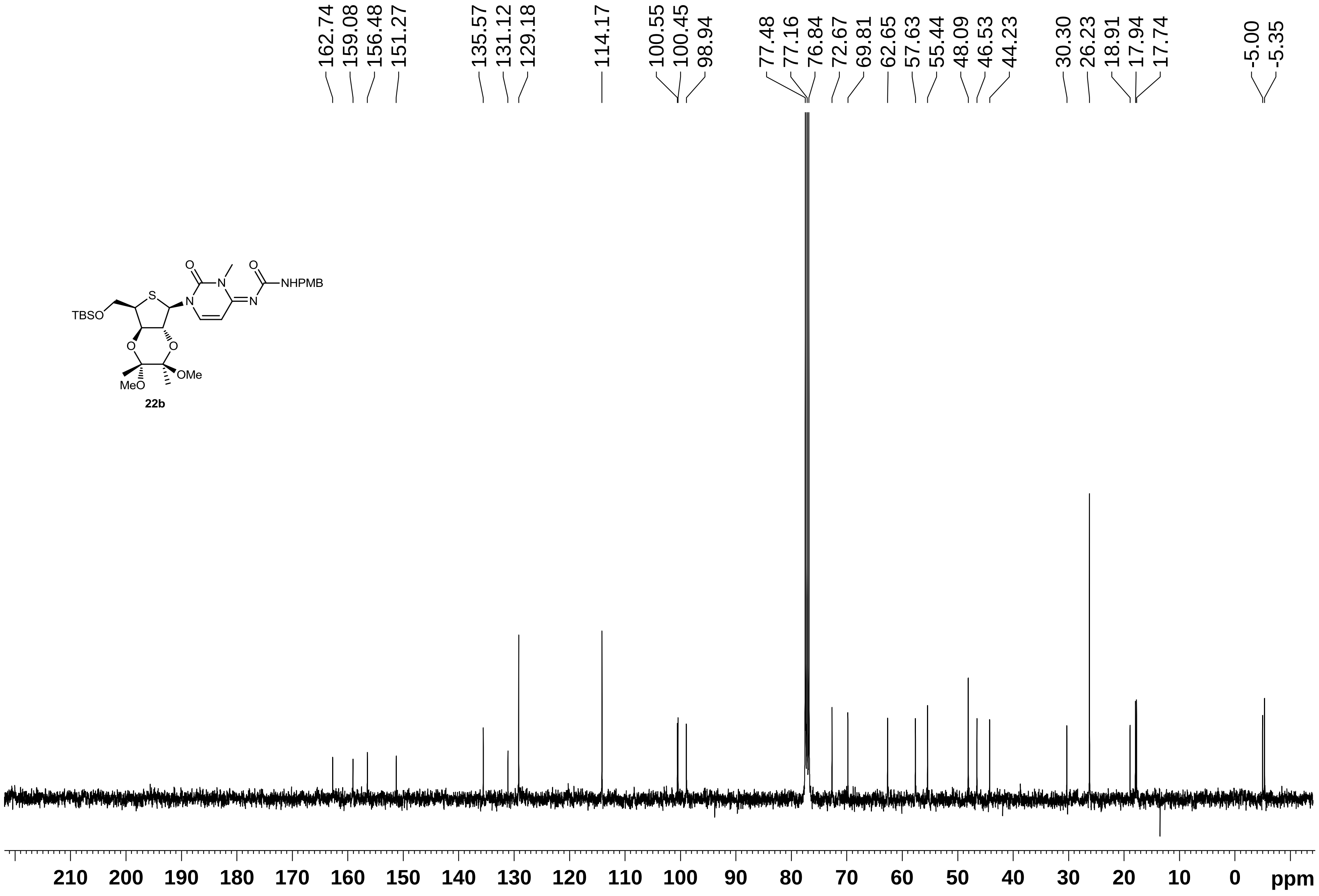

Supplementary Figure 48: ^{13}C NMR for compound **22b** ($CDCl_3$, 100 MHz).

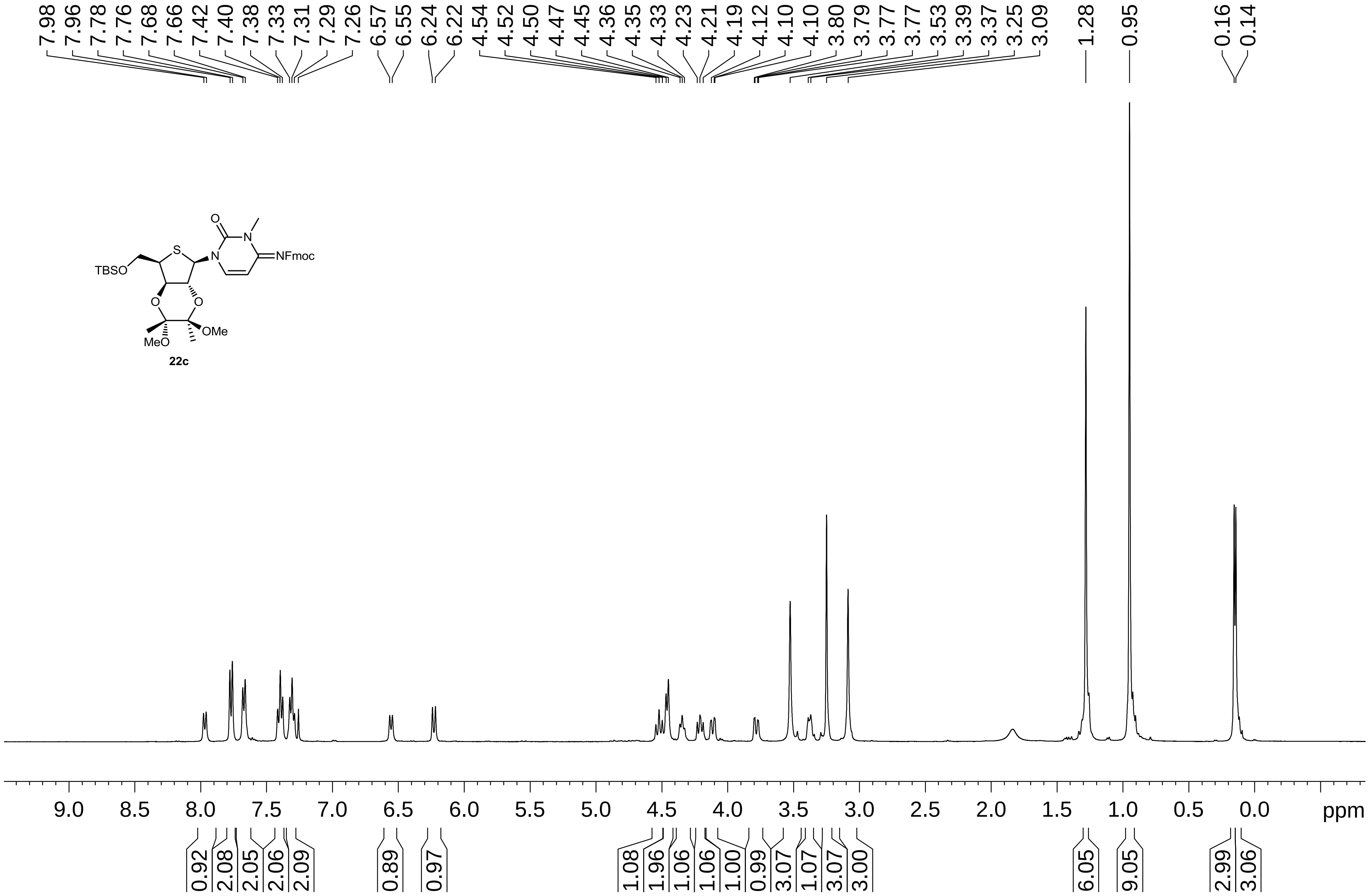

Supplementary Figure 49: ^{1}H NMR for compound **22c** ($CDCl_3$, 400 MHz).

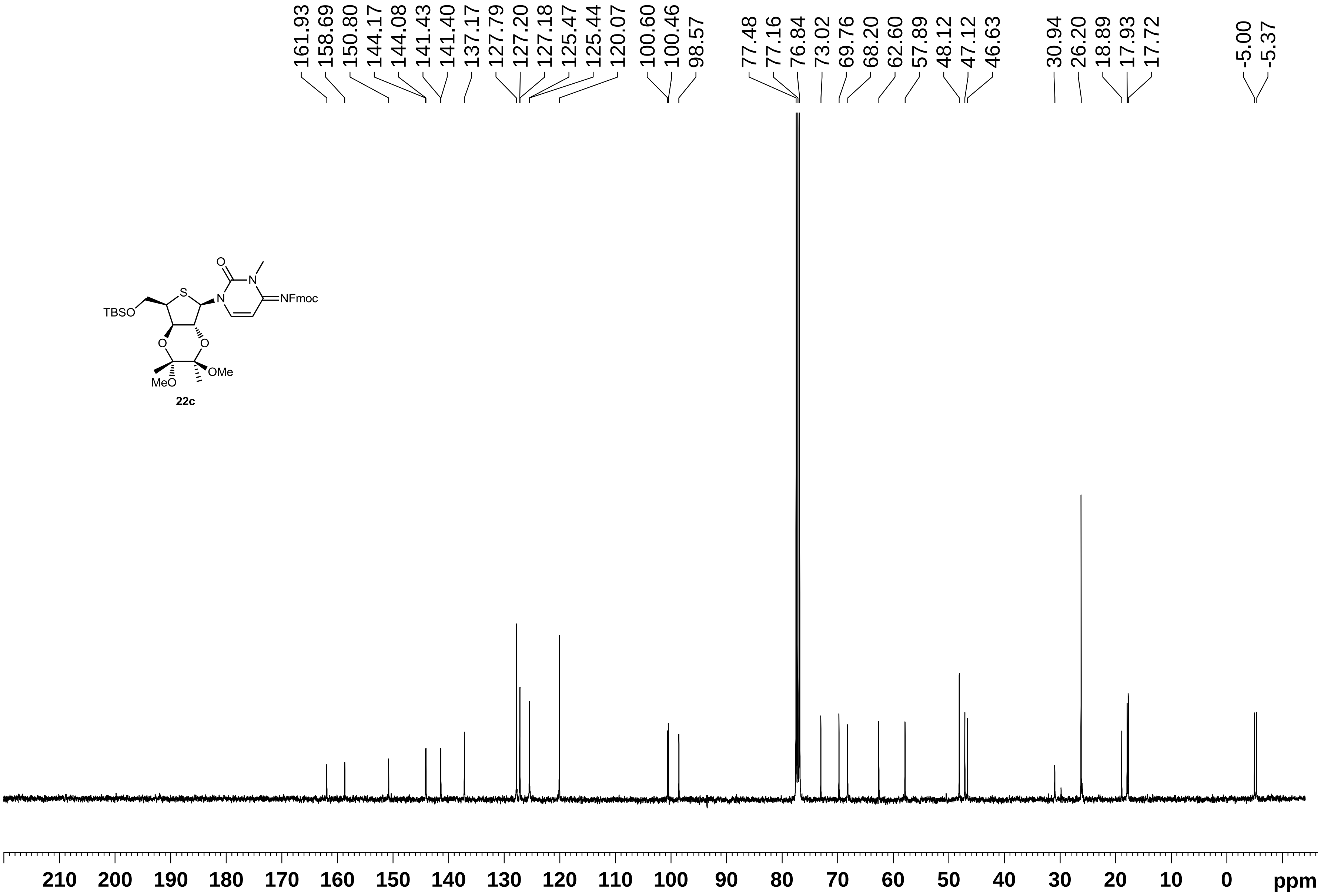

Supplementary Figure 50: ^{13}C NMR for compound **22c** ($CDCl_3$, 100 MHz).

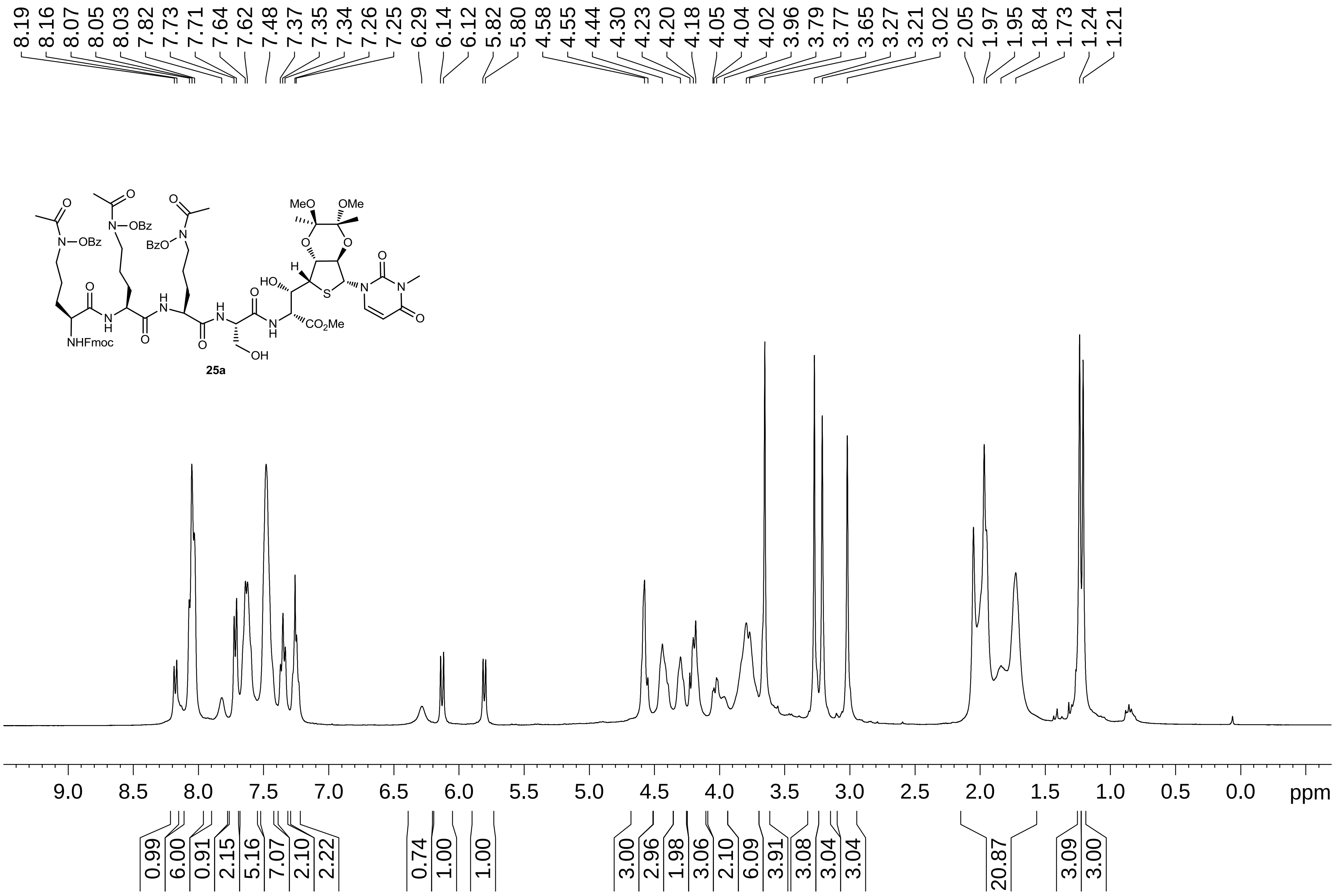

Supplementary Figure 51: ^{1}H NMR for compound **25a** ($CDCl_3$, 400 MHz).

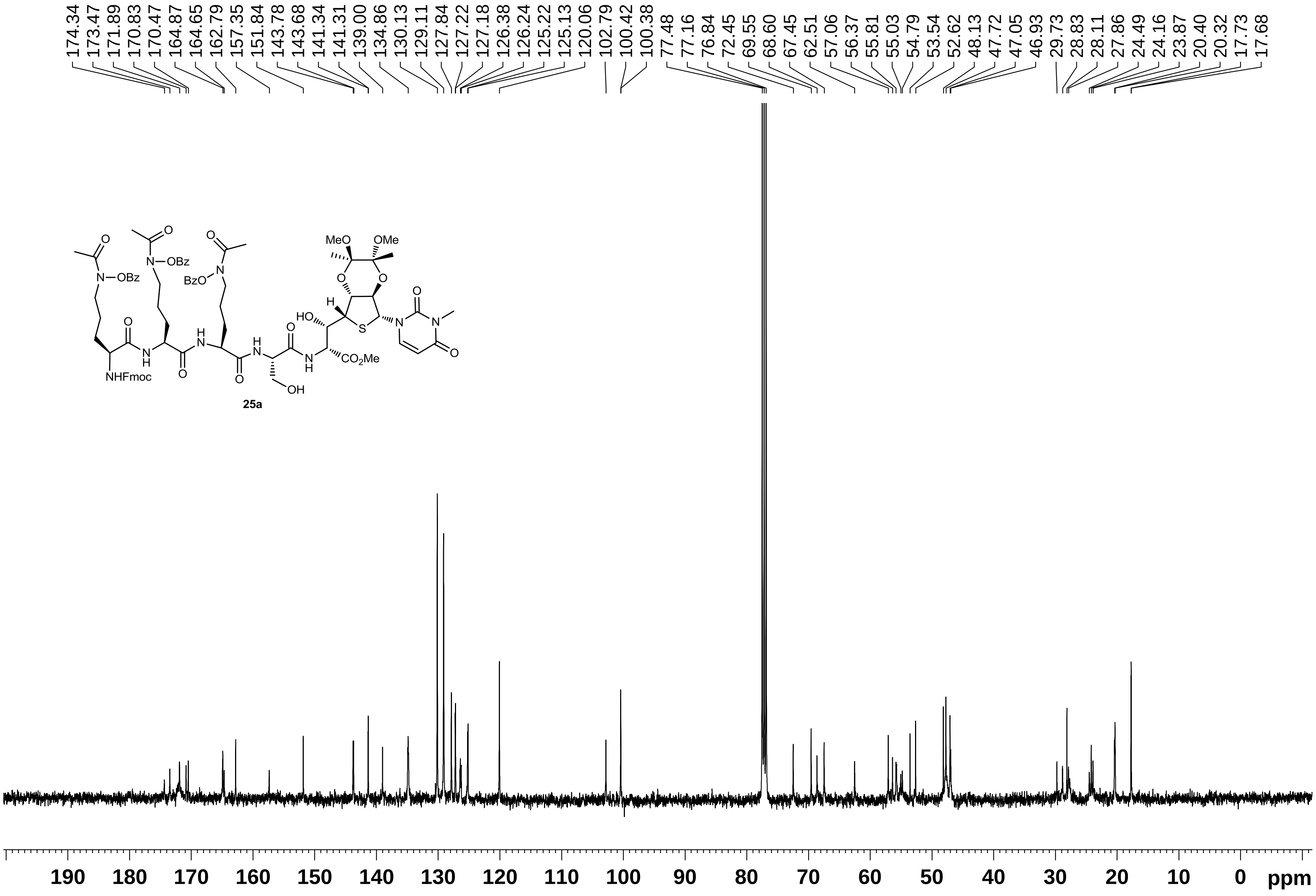

Supplementary Figure 52: ^{13}C NMR for compound **25a** ($CDCl_3$, 100 MHz).

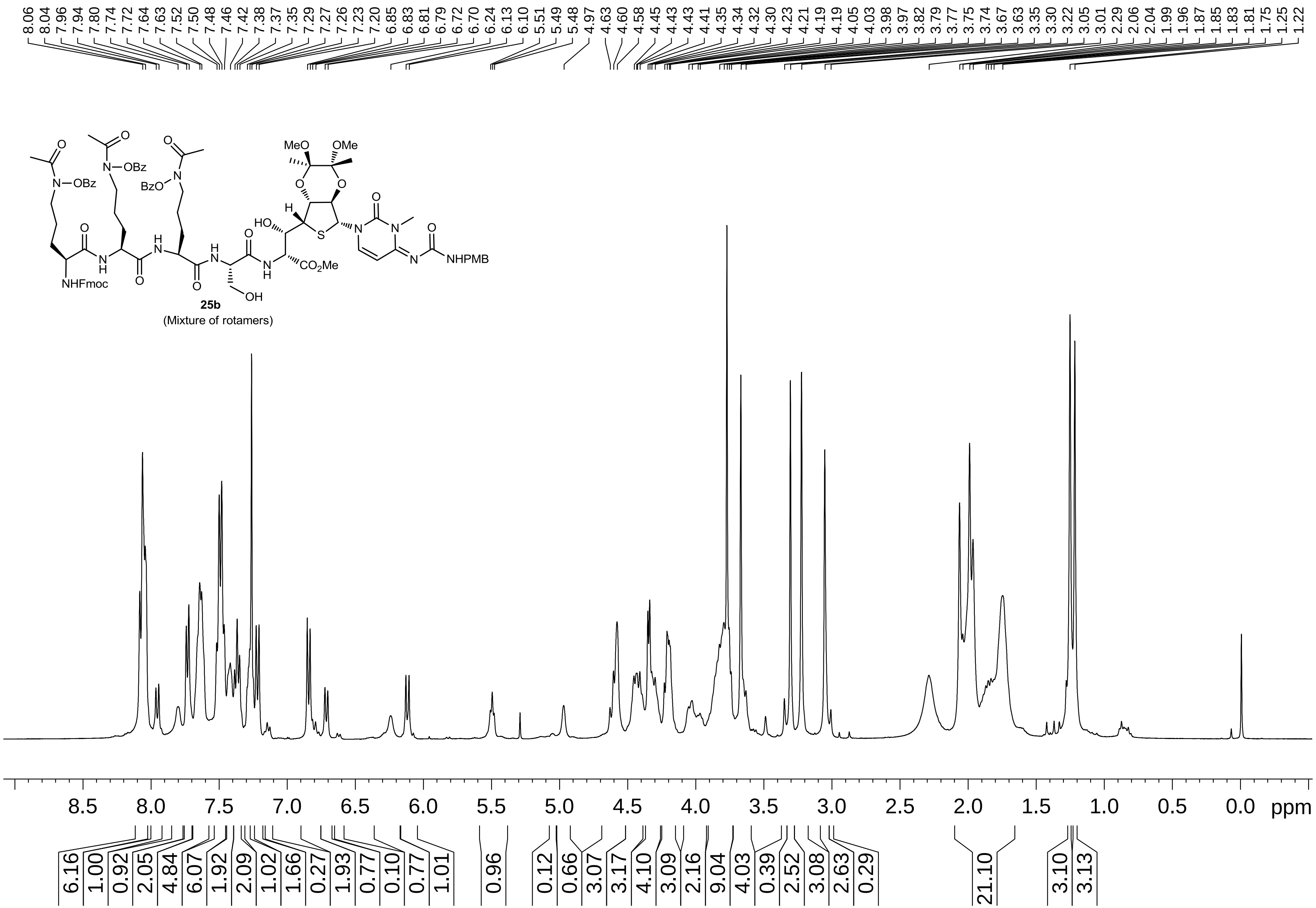

Supplementary Figure 53: ^{1}H NMR for compound **25b** ($CDCl_3$, 400 MHz).

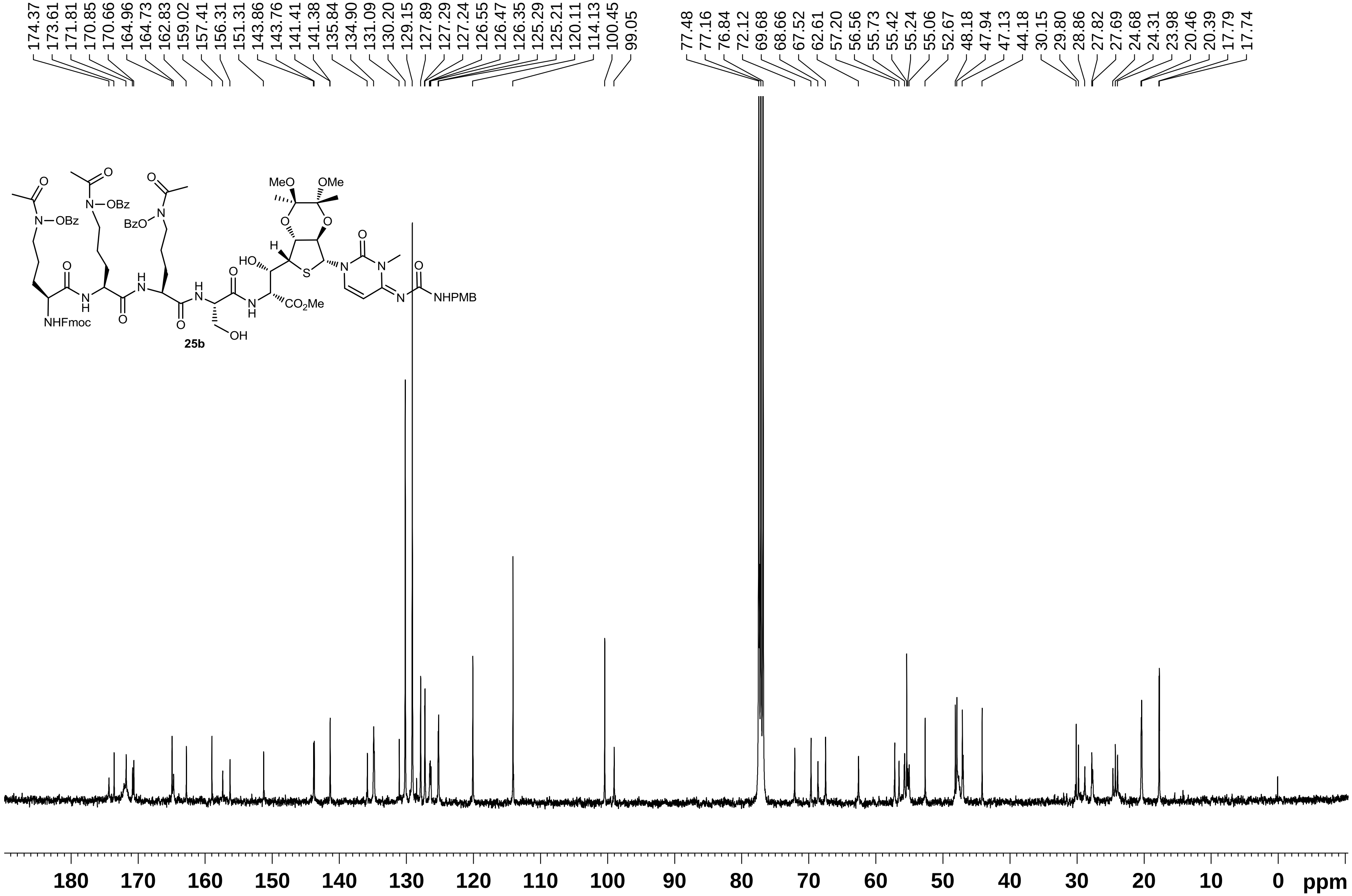

Supplementary Figure 54: ^{13}C NMR for compound **25b** ($CDCl_3$, 100 MHz).

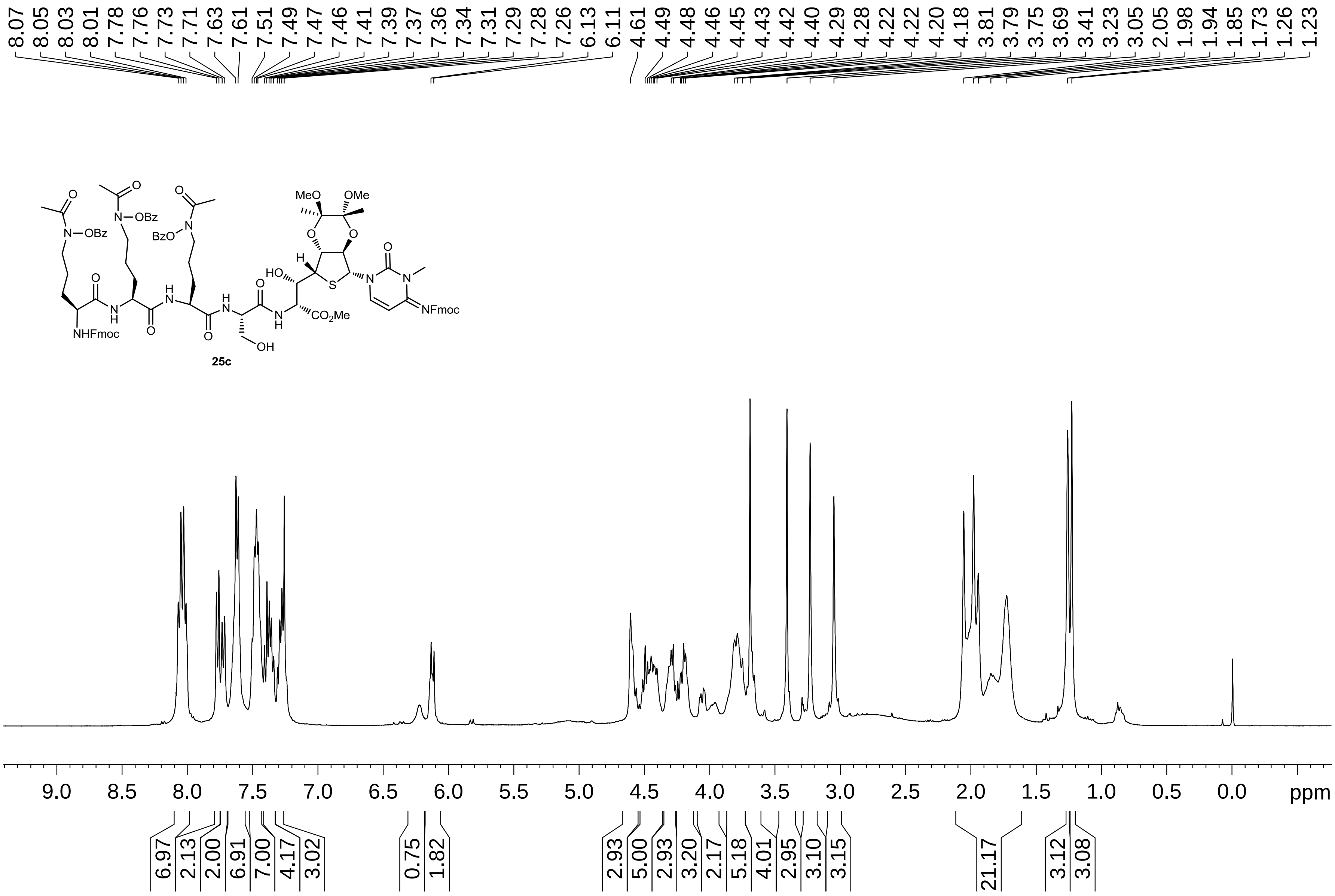

Supplementary Figure 55: ^{1}H NMR for compound **25c** ($CDCl_3$, 400 MHz).

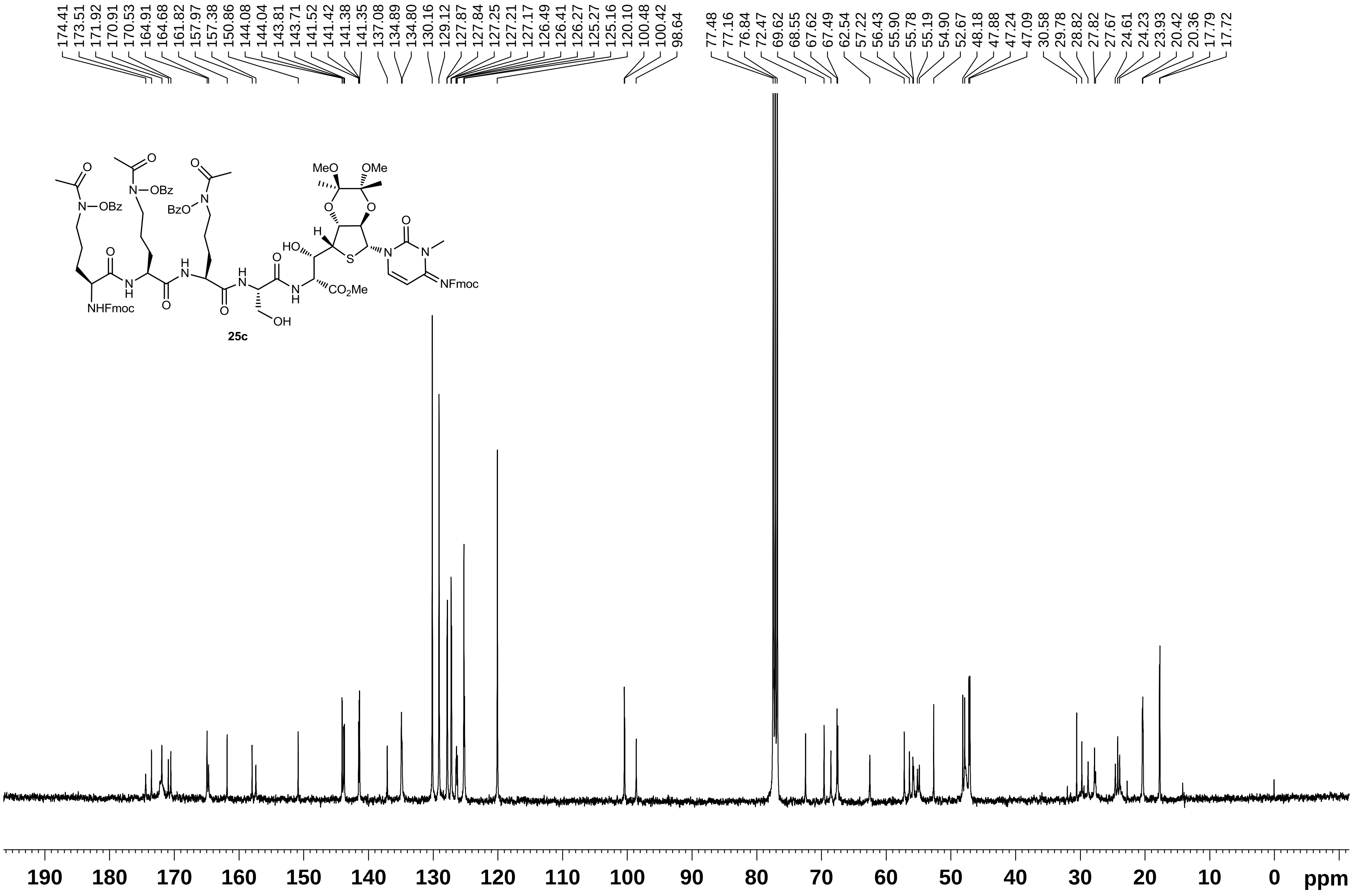

Supplementary Figure 56: ^{13}C NMR for compound **25c** ($CDCl_3$, 100 MHz).

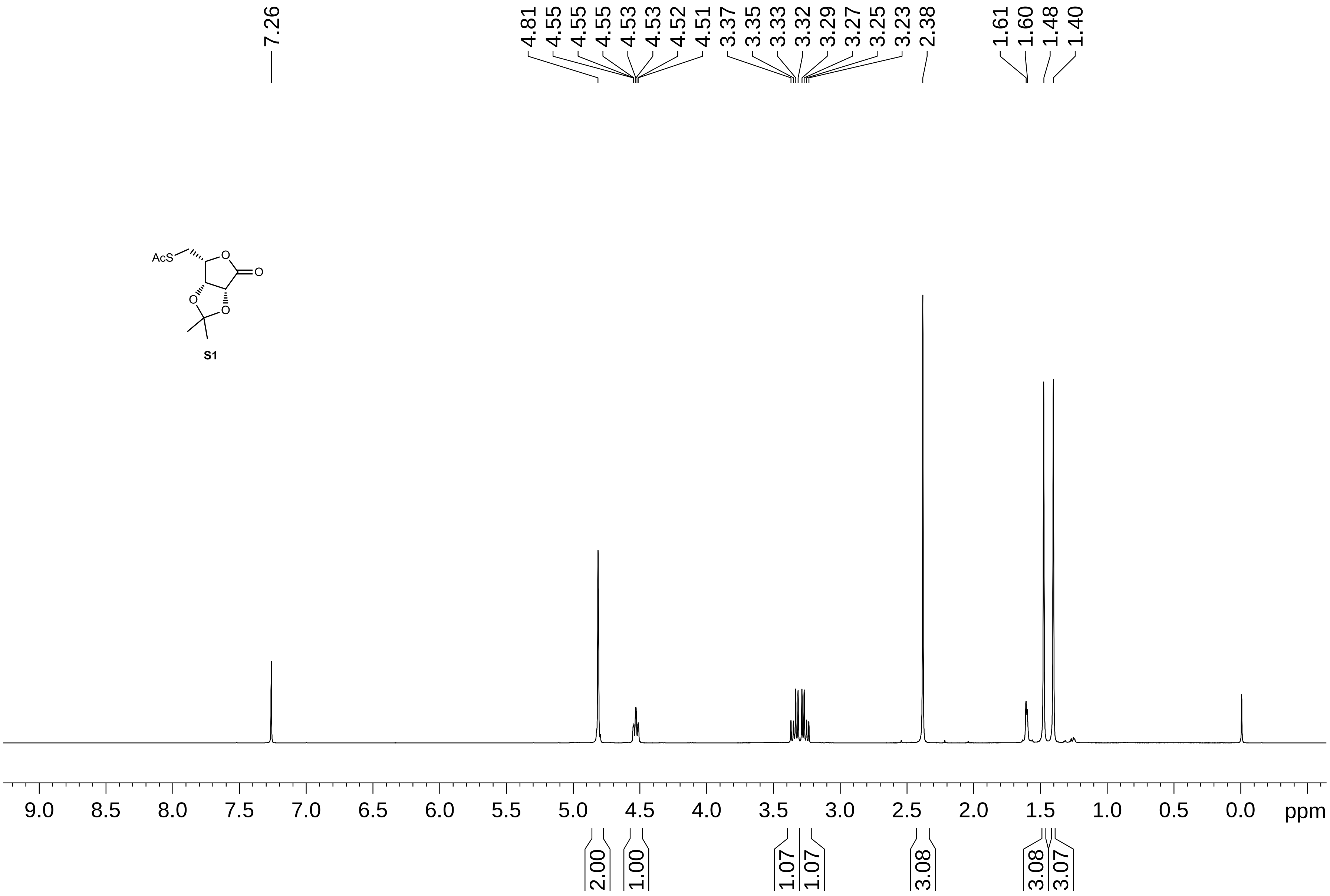

Supplementary Figure 57: ^{1}H NMR for compound **S1** ($CDCl_3$, 400 MHz).

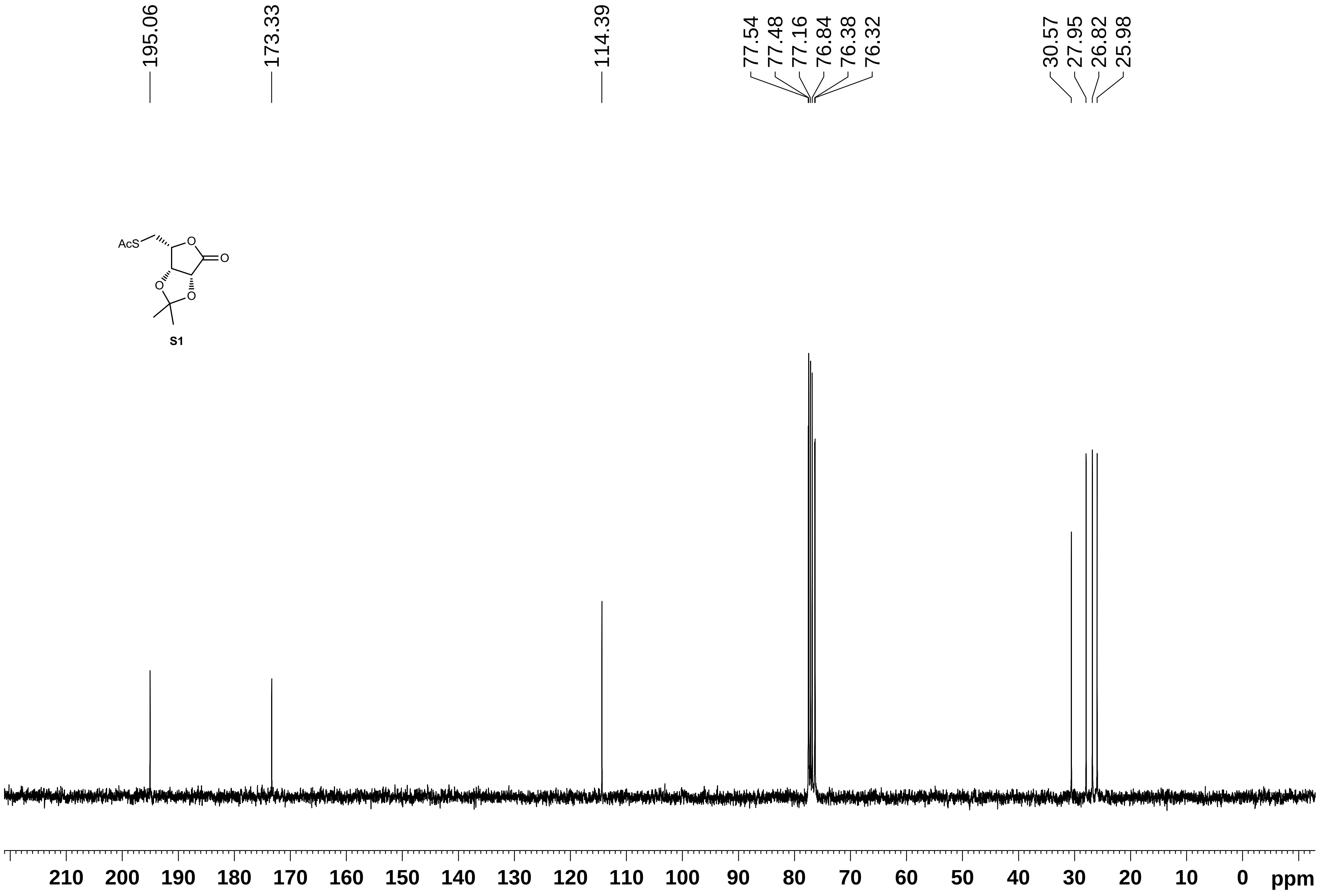

Supplementary Figure 58: ^{13}C NMR for compound **S1** ($CDCl_3$, 100 MHz).

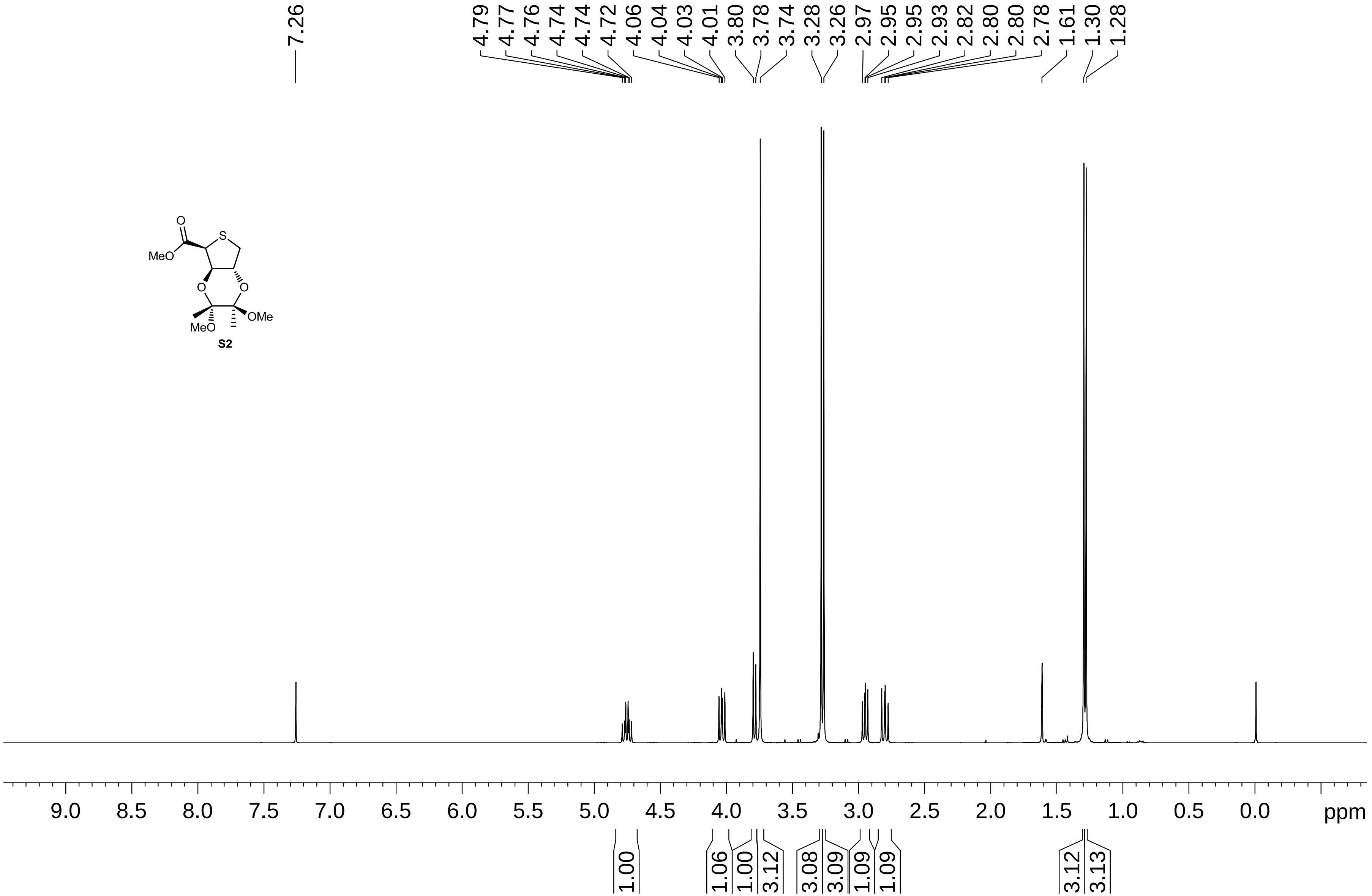

Supplementary Figure 59: ^{1}H NMR for compound **S2** ($CDCl_3$, 400 MHz).

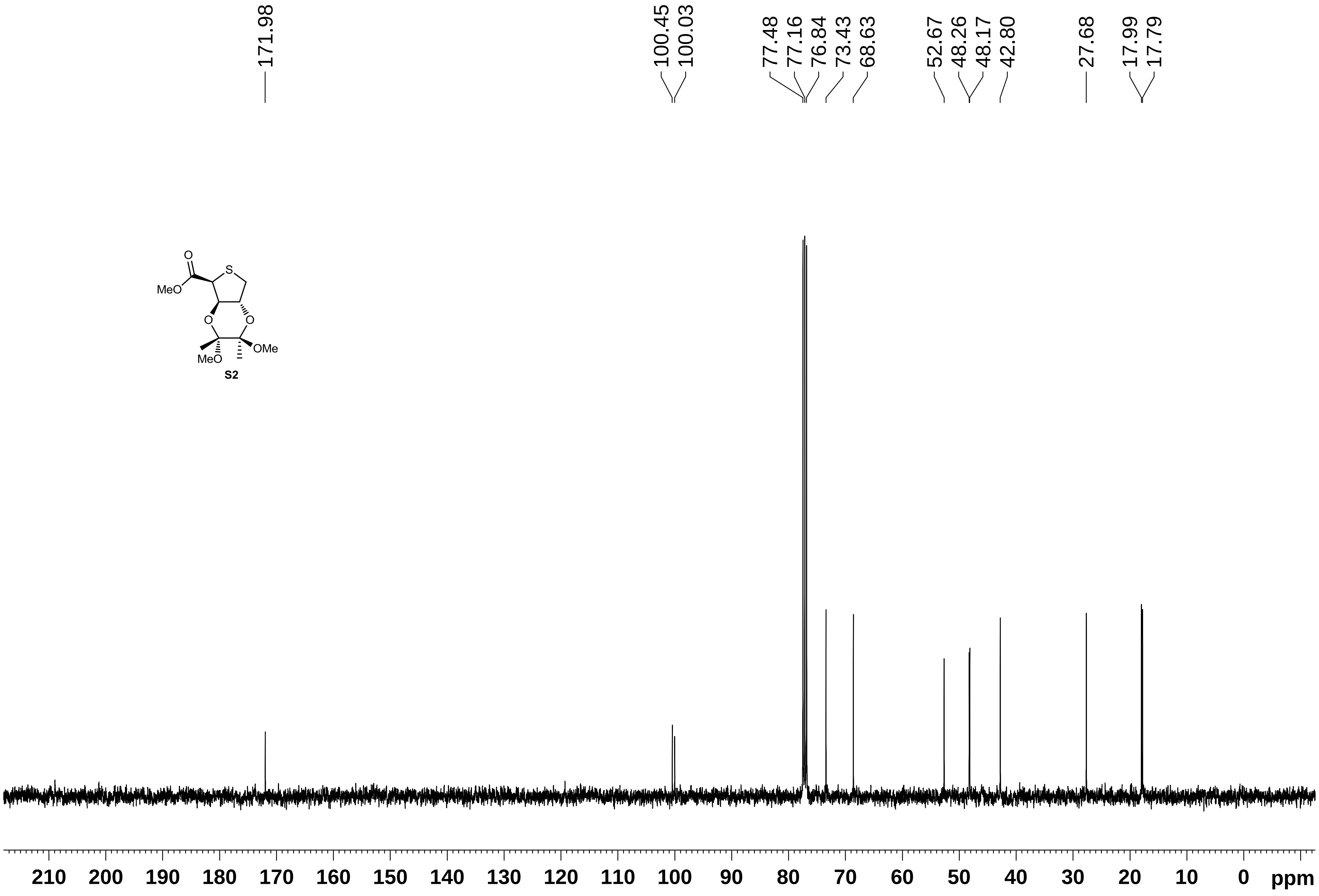

Supplementary Figure 60: ^{13}C NMR for compound **S2** ($CDCl_3$, 100 MHz).

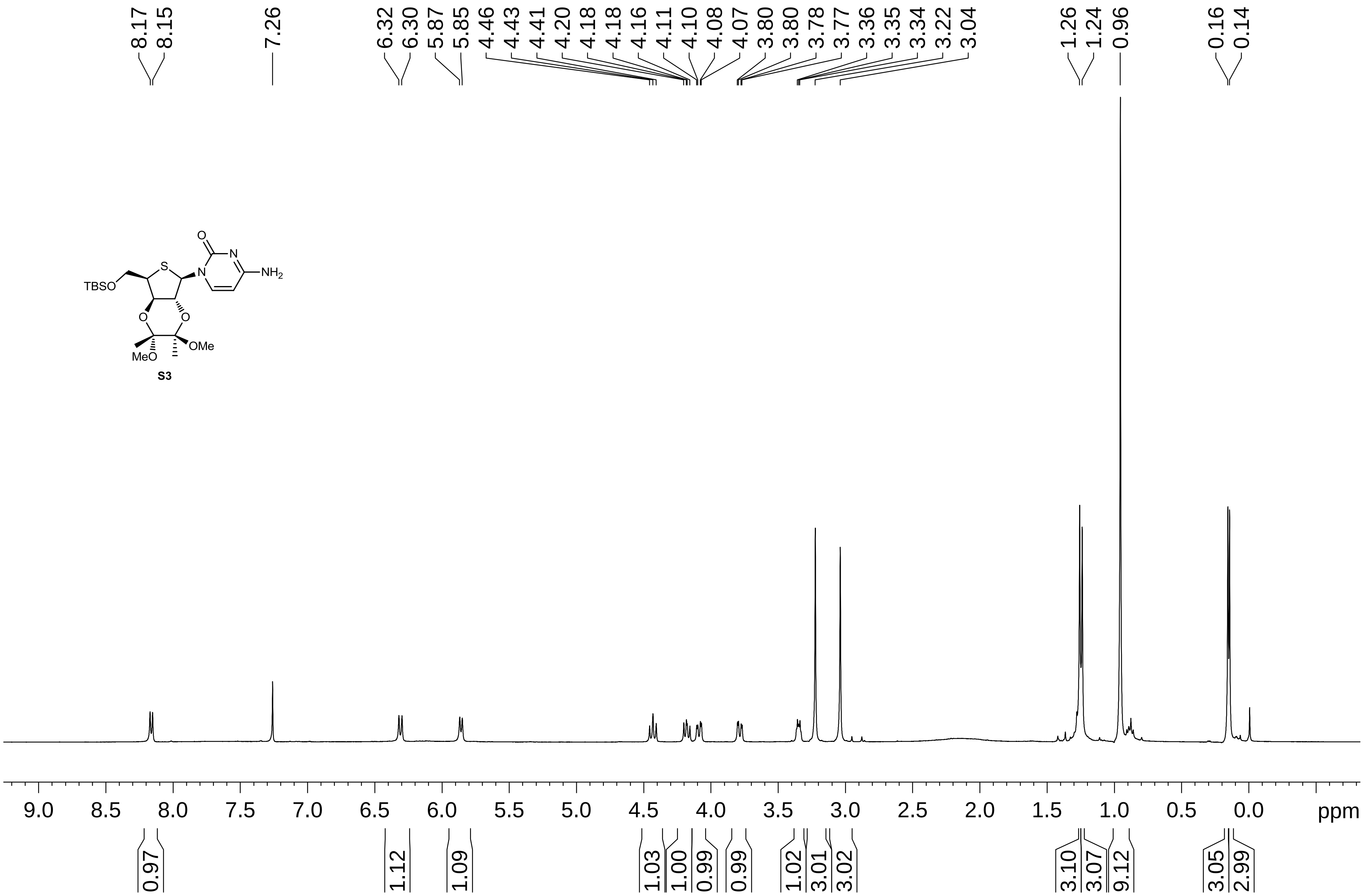

Supplementary Figure 61: ^{1}H NMR for compound **S3** ($CDCl_3$, 400 MHz).

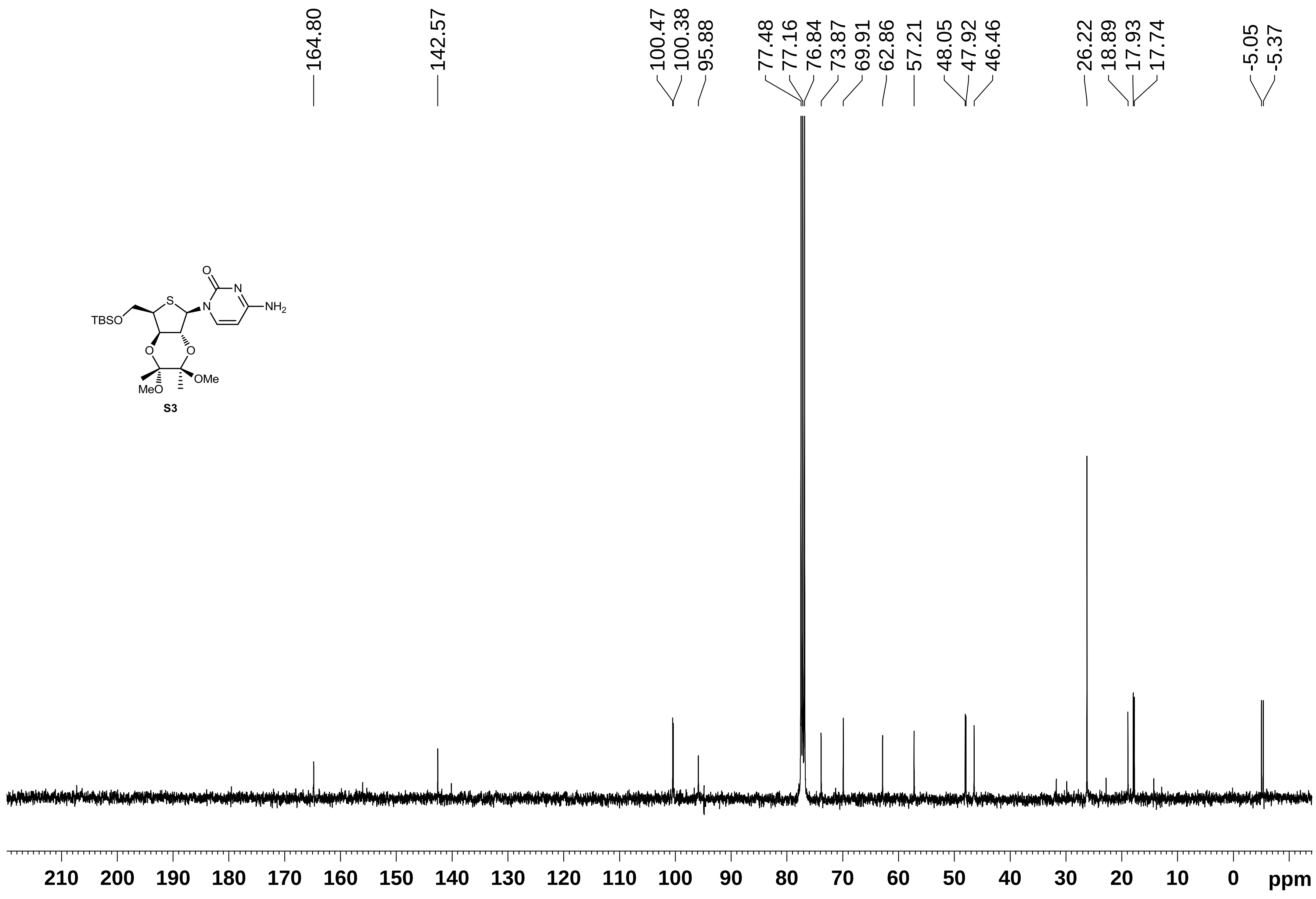

Supplementary Figure 62: ^{13}C NMR for compound **S3** ($CDCl_3$, 100 MHz).

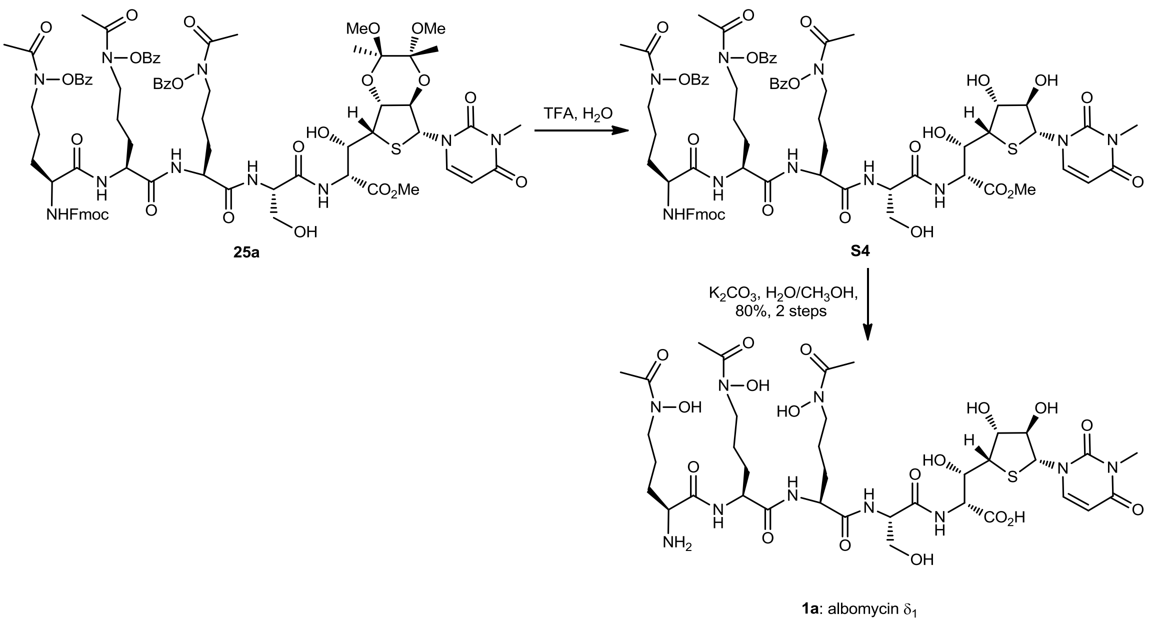

Supplementary Figure 63: Synthesis of compound **1a**.

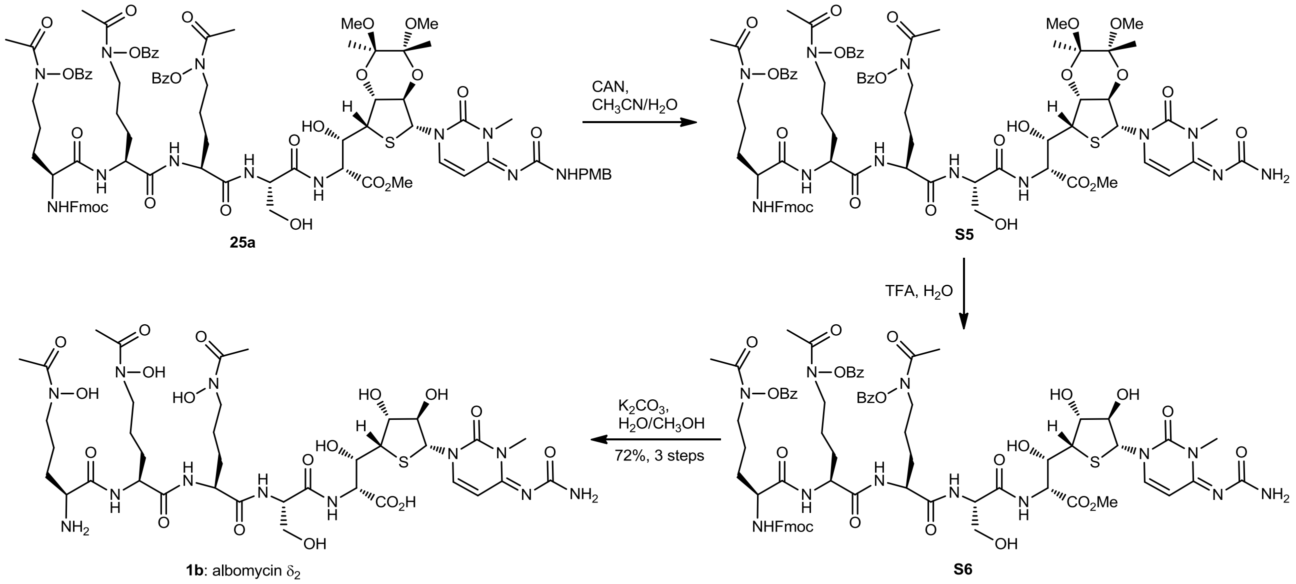

Supplementary Figure 64: Synthesis of compound **1b**.

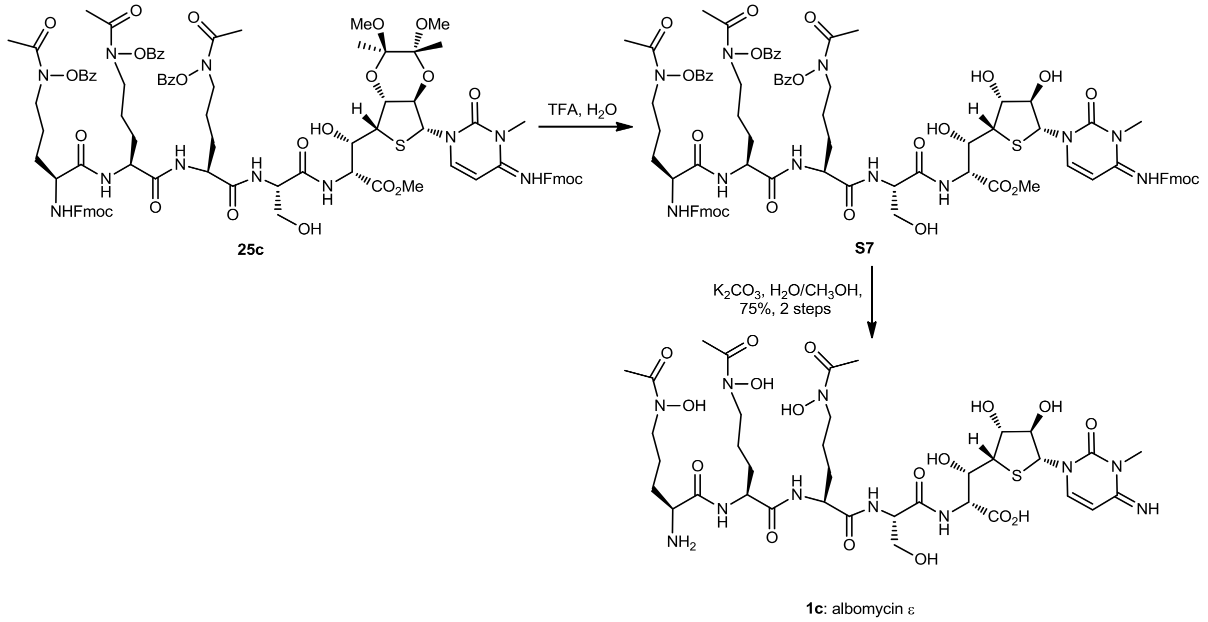

Supplementary Figure 65: Synthesis of compound **1c**.

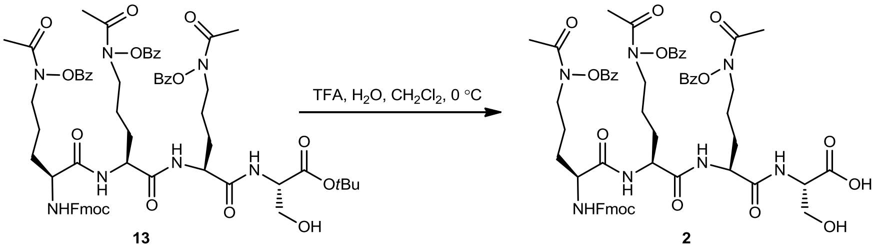

Supplementary Figure 66: Synthesis of compound **2**

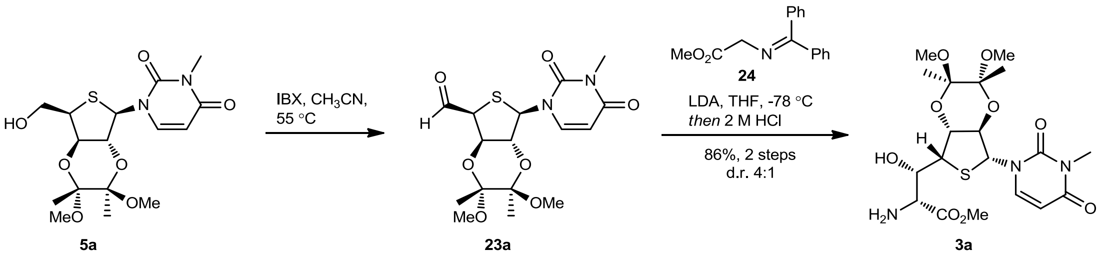

Supplementary Figure 67: Synthesis of compound **3a**.

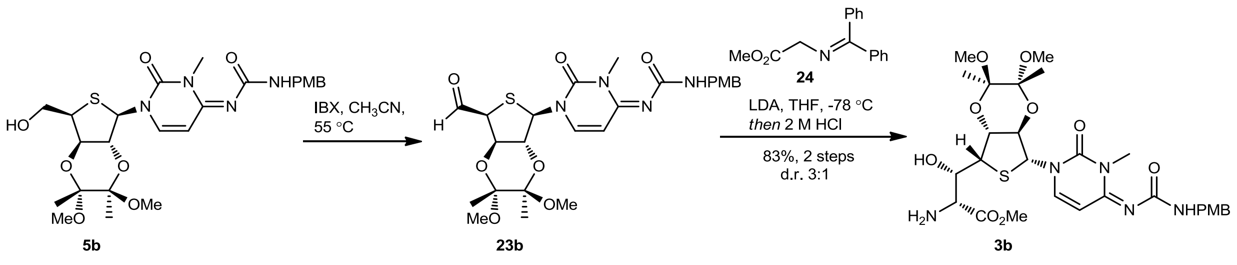

Supplementary Figure 68: Synthesis of compound **3b**.

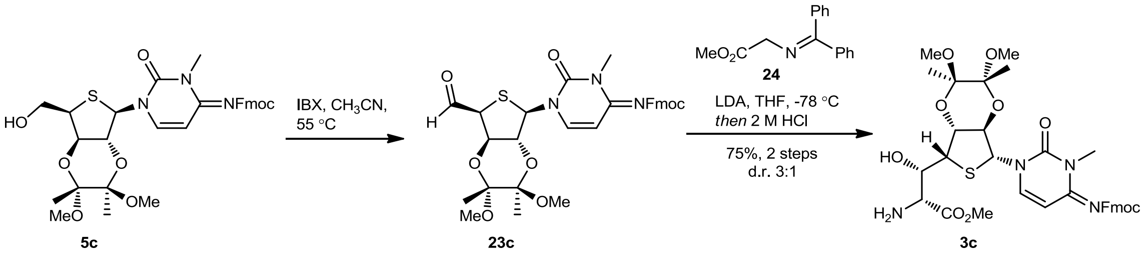

Supplementary Figure 69: Synthesis of compound **3c**.

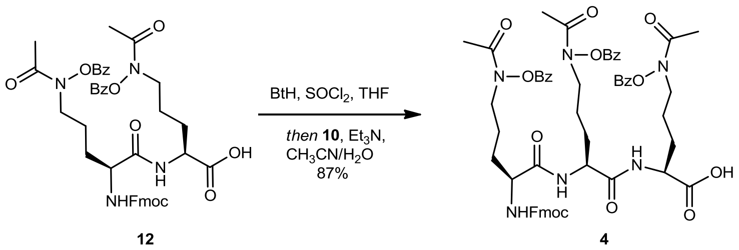

Supplementary Figure 70: Synthesis of compound **4**.

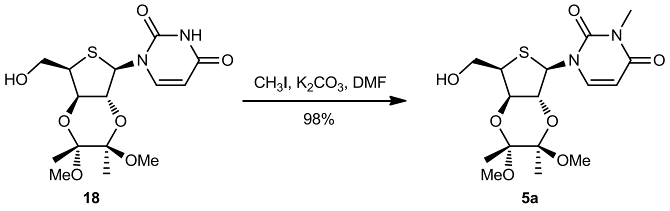

Supplementary Figure 71: Synthesis of compound **5a**.

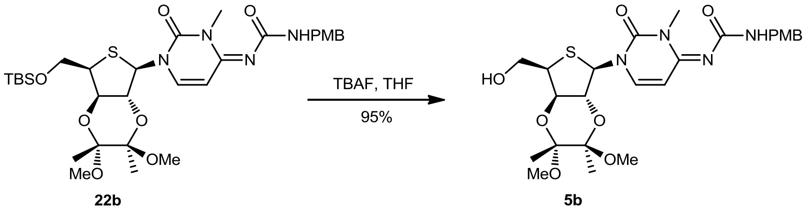

Supplementary Figure 72: Synthesis of compound **5b**.

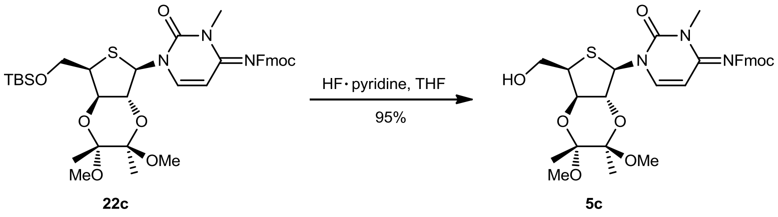

Supplementary Figure 73: Synthesis of compound **5c**.

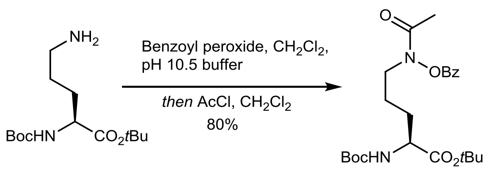

8 **6**

Supplementary Figure 74: Synthesis of compound **6**.

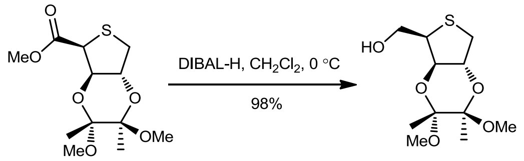

S2 **7**

Supplementary Figure 75: Synthesis of compound **7**.

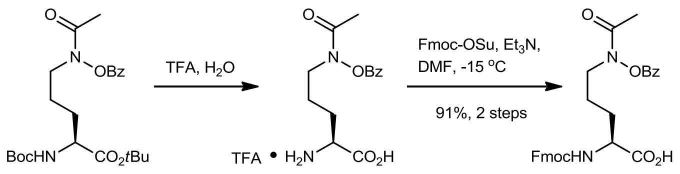

6 **10** **11**

Supplementary Figure 76: Synthesis of compound **11**.

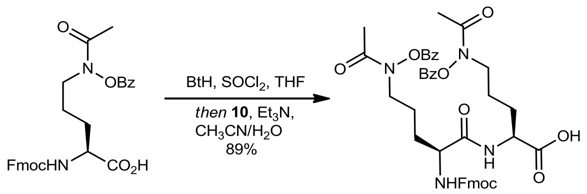

11 **12**

Supplementary Figure 77: Synthesis of compound **12**.

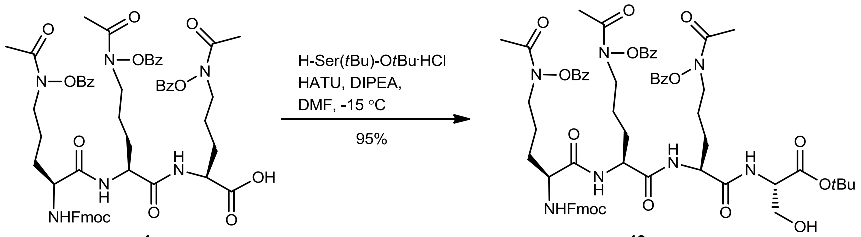

4 **13**

Supplementary Figure 78: Synthesis of compound **13**.

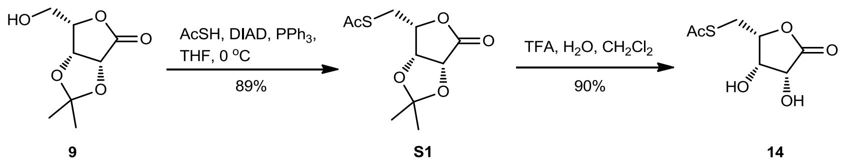

Supplementary Figure 79: Synthesis of compound **14**.

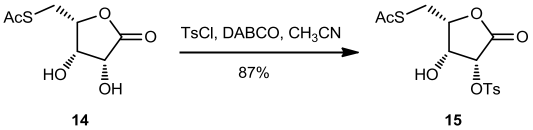

Supplementary Figure 80: Synthesis of compound **15**.

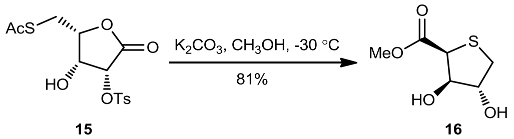

Supplementary Figure 81: Synthesis of compound **16**.

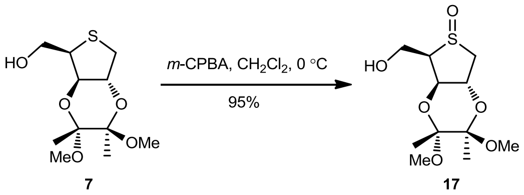

Supplementary Figure 82: Synthesis of compound **17**.

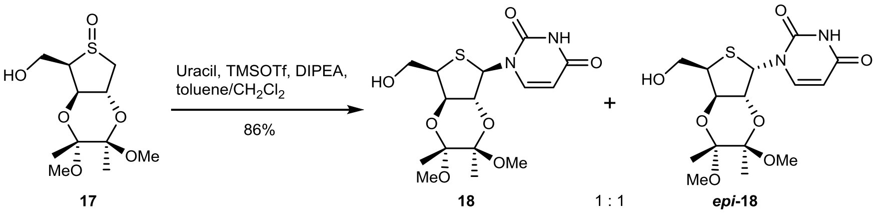

Supplementary Figure 83: Synthesis of compound **18**.

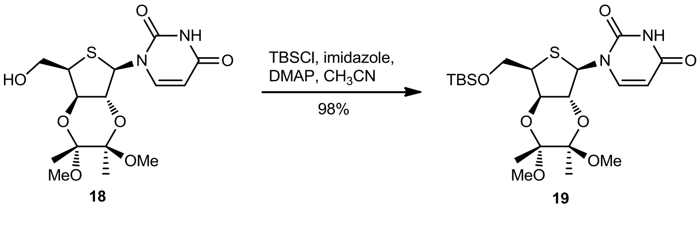

Supplementary Figure 84: Synthesis of compound **19**.

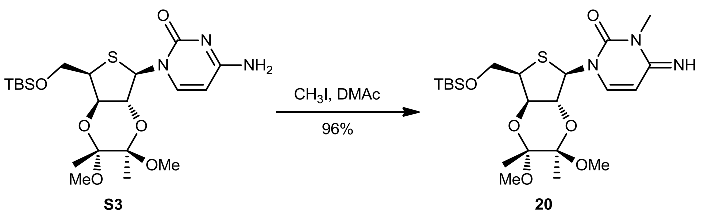

Supplementary Figure 85: Synthesis of compound **20**.

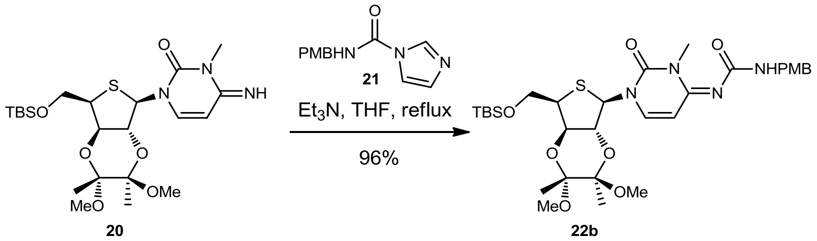

Supplementary Figure 86: Synthesis of compound **22b**.

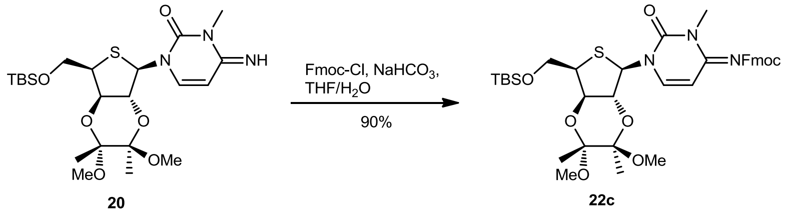

Supplementary Figure 87: Synthesis of compound **22c**.

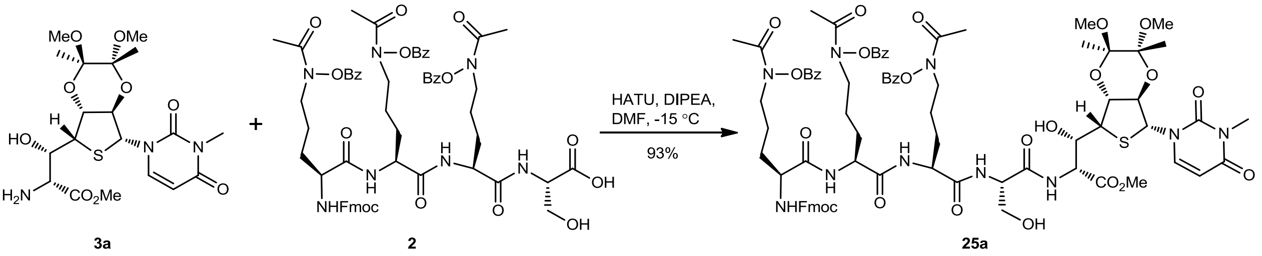

Supplementary Figure 88: Synthesis of compound **25a**.

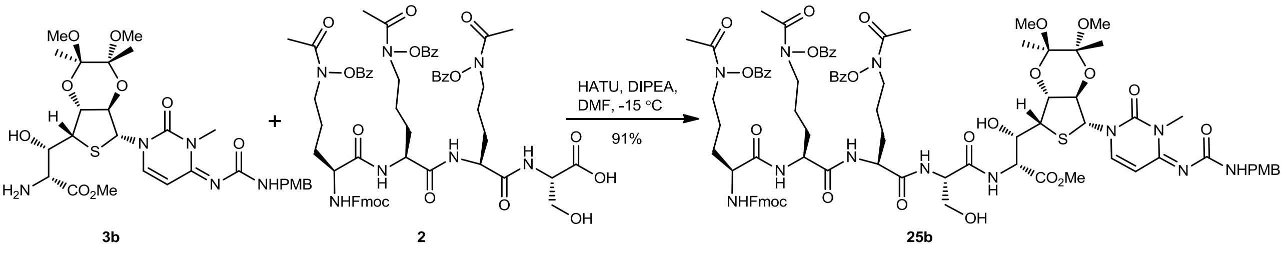

Supplementary Figure 89: Synthesis of compound **25b**.

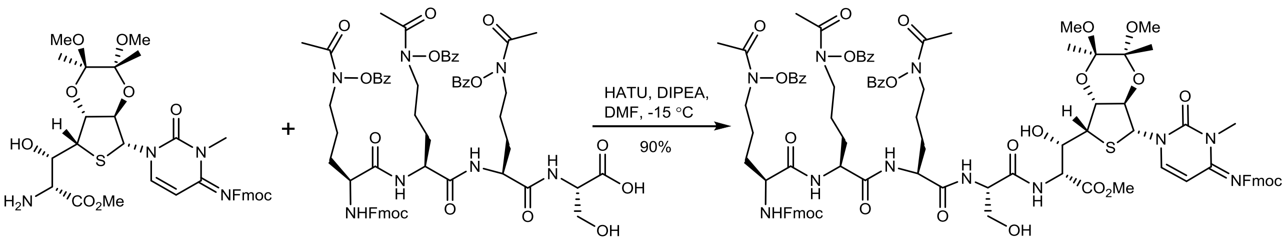

3c **2** **25c**

Supplementary Figure 90: Synthesis of compound **25c**.

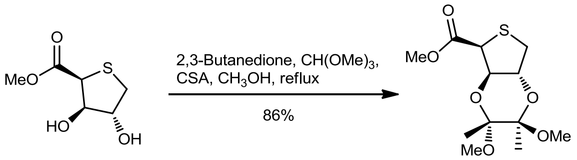

16 **S2**

Supplementary Figure 91: Synthesis of compound **S2**.

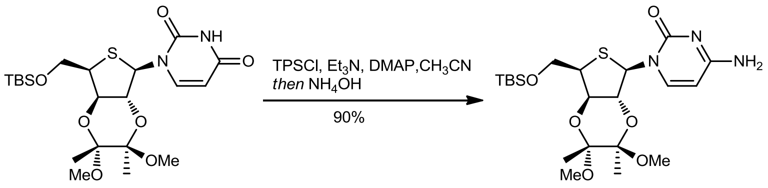

19 **S3**

Supplementary Figure 92: Synthesis of compound **S3**.

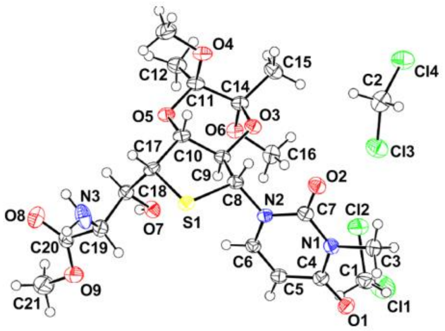

Supplementary Figure 93: X-ray crystal-structure of **3a**

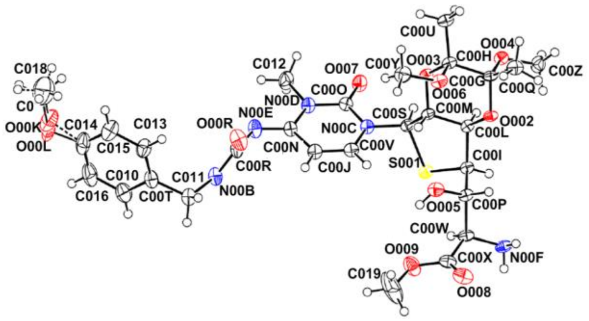

Supplementary Figure 94: X-ray crystal-structure of **3b**

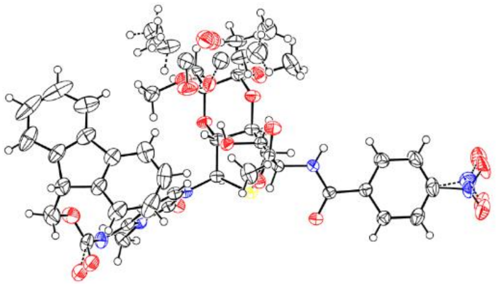

Supplementary Figure 95: X-ray crystal-structure of **3c'**

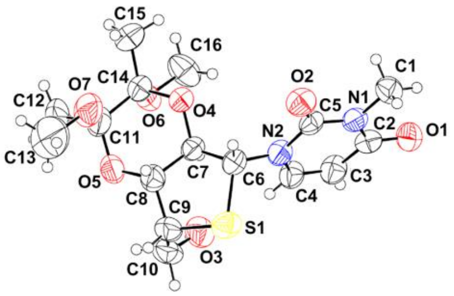

Supplementary Figure 96: X-ray crystal-structure of **5a**

Supplementary Tables

Supplementary Table 1: Tabulated comparison of ^{1}H NMR data of the synthetic albomycin δ_2 with that reported by Benz and coworkers[1].

Natural albomycin δ_2		Position	Synthetic albomycin δ_2	
δ_H (360 MHz)[a]	J (Hz)	H-6 Pyrimidin	δ_H (400 MHz)	J (Hz)
8.22 [1] d	8.5	H-5 Pyrimidin	8.21 [1] d	8.3
6.20 [1] d	8.5	H-1'	6.23 [1] d	8.2
5.93 [1] d	6.0	α CH Serine	5.95 [1] d	6.0
4.31–4.51 [7] m		α CH Ornithin	4.49 [2] t	6.4
			4.34–4.47 [5] m	
4.04 [1] t	6.0	N-C-H, O-C-H	3.77–3.96 [3] m	
3.90 [1] dd	12.0, 5.0	α-CH-Ornithin		
3.82 [1] dd	12.0, 5.0	β CH_2 Serine		
3.60–3.72 [7] m		δ CH_2 Ornithin, S-C-H	3.58–3.77 [7] m	
3.31 [3] s		N-CH_3 Pyrimidin	3.34 [3] s	
2.14 [9] s		N-$COCH_3$	2.16 [9] brs	
1.62–1.94 [12] m		β, γ CH_2 Ornithin	1.59–1.93 [12] m	

[a] ^{1}H and ^{13}C NMR chemical shifts of albomycin δ_2 were pH-dependent.

Supplementary Table 2: Tabulated comparison of ^{13}C NMR data of the synthetic albomycin δ_2 with that reported by Benz and coworkers[1].

Natural albomycin δ_2	position	Synthetic albomycin δ_2	Natural albomycin δ_2	position	Synthetic albomycin δ_2
δc (62.86 MHz)		δc (100MHz)	δc (62.86 MHz)		δc (100 MHz)
176.0		176.1	61.8	β-$\underline{C}H_2$ Serine	61.8
174.8		174.4	58.3		58.4
174.7		174.4	56.2	α $\underline{C}$H Serine	56.3
174.3	C=O	174.2	54.3		54.3
174.0			54.1	α $\underline{C}$H Ornithin	54.1
171.7		171.7	53.3		53.9
170.3			50.8		50.7 (51.7)
168.1		168.2	48.0	$\underline{C}H_2$ Ornithin	48.0
156.2	C-4 Pyrimidin	156.2	47.7	$\underline{C}H_2$ Ornithin	
153.6	C-2 Pyrimidin	153.2	30.6		30.7 (30.2)
138.8	C-6 Pyrimidin	138.8	28.7		28.8 (28.9)
98.0	C-5 Pyrimidin	98.0	23.2		23.3
80.0		79.9	23.0		23.1
75.7		75.6	22.1		22.5
69.7		69.7	20.0	N-CO$\underline{C}H_3$	20.1
63.8		63.7			

Supplementary Table 3: X-ray crystallographic data for **3a (**CCDC 1839506).

Identification code	ov_lzh1102
Empirical formula	$C_{21}H_{33}Cl_4N_3O_9S$
Formula weight	645.36
Temperature/K	293(2)
Crystal system	orthorhombic
Space group	$P2_12_12_1$
a/Å	9.69310(10)
b/Å	14.41014(13)
c/Å	20.58327(18)
α/°	90
β/°	90
γ/°	90
Volume/Å^3	2875.05(5)
Z	4
ρ_{calc}g/cm^3	1.491
μ/mm^{-1}	4.883
F(000)	1344.0
Crystal size/mm^3	0.2 × 0.1 × 0.05
Radiation	CuKα (λ = 1.54184)
2Θ range for data collection/°	7.488 to 146.064
Index ranges	-11 ≤ h ≤ 8, -17 ≤ k ≤ 17, -25 ≤ l ≤ 25
Reflections collected	15346
Independent reflections	5501 [R_{int} = 0.0285, R_{sigma} = 0.0308]
Data/restraints/parameters	5501/0/356
Goodness-of-fit on F^2	1.003
Final R indexes [I>=2σ (I)]	R_1 = 0.0363, wR_2 = 0.0919
Final R indexes [all data]	R_1 = 0.0375, wR_2 = 0.0932
Largest diff. peak/hole / e Å^{-3}	0.28/-0.26
Flack parameter	-0.001(7)

Supplementary Table 4: X-ray crystallographic data for **3b (**CCDC 1839508).

Identification code	lzh1109
Empirical formula	$C_{28}H_{39}N_5O_{10}S$
Formula weight	637.70
Temperature/K	149.99(10)
Crystal system	monoclinic
Space group	$P2_1$
a/Å	13.05214(16)
b/Å	8.71770(8)
c/Å	19.5385(2)
α/°	90
β/°	108.4323(13)
γ/°	90
Volume/Å^3	2109.13(4)
Z	2
ρ_{calc}g/cm^3	1.004
μ/mm^{-1}	1.083
F(000)	676.0
Crystal size/mm^3	0.2 × 0.05 × 0.05
Radiation	CuKα (λ = 1.54184)
2Θ range for data collection/°	7.224 to 145.336
Index ranges	$-16 \le h \le 16$, $-7 \le k \le 10$, $-23 \le l \le 24$
Reflections collected	30712
Independent reflections	6620 [R_{int} = 0.0380, R_{sigma} = 0.0300]
Data/restraints/parameters	6620/19/431
Goodness-of-fit on F^2	0.987
Final R indexes [I>=2σ (I)]	R_1 = 0.0345, wR_2 = 0.0949
Final R indexes [all data]	R_1 = 0.0356, wR_2 = 0.0963
Largest diff. peak/hole / e Å^{-3}	0.29/-0.26
Flack parameter	0.022(12)

Supplementary Table 5: X-ray crystallographic data for **3c' (**CCDC 1839510).

Identification code	zs1120
Empirical formula	$C_{45}H_{51}N_5O_{15}S$
Formula weight	933.96
Temperature/K	149.99(10)
Crystal system	monoclinic
Space group	I2
a/Å	13.50750(10)
b/Å	17.2933(2)
c/Å	19.4909(2)
α/°	90
β/°	91.3210(10)
γ/°	90
Volume/Å^3	4551.65(8)
Z	4
ρ_{calc}g/cm^3	1.363
μ/mm^{-1}	1.272
F(000)	1968.0
Crystal size/mm^3	0.2 × 0.2 × 0.15
Radiation	CuKα (λ = 1.54184)
2Θ range for data collection/°	6.834 to 134.128
Index ranges	-15 ≤ h ≤ 16, -16 ≤ k ≤ 20, -23 ≤ l ≤ 20
Reflections collected	16986
Independent reflections	6895 [R_{int} = 0.0196, R_{sigma} = 0.0201]
Data/restraints/parameters	6895/25/667
Goodness-of-fit on F^2	1.062
Final R indexes [I>=2σ (I)]	R_1 = 0.0321, wR_2 = 0.0841
Final R indexes [all data]	R_1 = 0.0324, wR_2 = 0.0845
Largest diff. peak/hole / e Å^{-3}	0.29/-0.22
Flack parameter	0.006(8)

Supplementary Table 6: X-ray crystallographic data for **5a (**CCDC 1839504).

Identification code	lzh07106_sq
Empirical formula	$C_{16}H_{24}N_2O_7S$
Formula weight	388.43
Temperature/K	293(2)
Crystal system	monoclinic
Space group	$P2_1$
a/Å	7.7283(5)
b/Å	11.0154(5)
c/Å	13.8873(8)
α/°	90
β/°	102.347(6)
γ/°	90
Volume/Å^3	1154.89(12)
Z	2
ρ_{calc}g/cm^3	1.117
μ/mm^{-1}	0.173
F(000)	412.0
Crystal size/mm^3	? × ? × ?
Radiation	MoKα (λ = 0.71073)
2Θ range for data collection/°	6.7 to 52.736
Index ranges	$-9 \le h \le 9$, $-13 \le k \le 13$, $-17 \le l \le 17$
Reflections collected	8564
Independent reflections	4718 [R_{int} = 0.0234, R_{sigma} = 0.0456]
Data/restraints/parameters	4718/1/241
Goodness-of-fit on F^2	1.006
Final R indexes [I>=2σ (I)]	R_1 = 0.0495, wR_2 = 0.1117
Final R indexes [all data]	R_1 = 0.0694, wR_2 = 0.1267
Largest diff. peak/hole / e Å^{-3}	0.27/-0.17
Flack parameter	0.14(5)

Supplementary Methods

General Experimental

Unless otherwise specified, all reactions were carried out under N_2 atmosphere in oven-dried glassware. Tetrahydrofuran (THF) was distilled over sodium and benzophenone, dichloromethane (CH_2Cl_2), *N*, *N*-dimethylformamide (DMF), triethylamine (Et_3N) and *N*, *N*-diisopropylamine (DIPEA) over calcium hydride. All other solvents as well as starting materials and reagents were obtained from commercial sources and used without further purification. Reactions were monitored by analytical thin-layer chromatography (TLC) on Merck silica gel 60 F_{254} plates (0.25 mm), visualized by ultraviolet light and/or by staining with phosphomolybdic acid in EtOH. Retention factor (R_f) values reported were measured using a 5 × 2 cm TLC plate in a developing chamber containing the solvent system described.

^{1}H NMR spectra were obtained on an Agilent 400MR or 600MR DD2 spectrometer at ambient temperature. Chemical shifts were reported in parts per million (ppm), relative to either a tetramethylsilane (TMS) internal standard or the signals due to the solvent. ^{13}C NMR spectra were obtained with proton decoupling on an Agilent 400MR or 600MR DD2 (100 MHz or 150 MHz) spectrometer and were reported in ppm with residual solvent for internal standard. Multiplicity is defined as: s = singlet; d = doublet; t = triplet; q = quartet; m = multiplet, br = broad or combinations of the above. Coupling constants (J) are reported in Hertz. High resolution mass spectra were obtained on a Bruker SolariX 7.0T spectrometer. Melting point was determined by WRS-2A Digital Melting Point Apparatus. Optical rotations were measured with a Rudolph polarimeter. Crystallographic data were obtained from a single-crystal X-ray diffractometer.

Synthesis of compound 1a.

25a (50 mg, 0.031 mmol) was dissolved in TFA (2 mL) and H_2O (10 μL), the reaction mixture was stirred at room temperature for 1 h, concentrated *in vacuo* to afford **S4**, which was used directly in the next step without further purification.

To a solution of **S4** in CH_3OH (3 mL) was added a solution of K_2CO_3 (8.6 mg, 0.062 mmol, 2.0 equiv) in H_2O (57 μL). The resulting solution was stirred for 1 h, before same amount of K_2CO_3 solution was added. The resulting reaction mixture was stirred until completely converted to **1a.** The pH of the reaction was adjusted to 7 with 0.5 M aqueous HCl. The mixture was concentrated *in vacuo*. The residue was washed with CH_2Cl_2 and then purified by Sephadex™ G-15 to afford **1a** (24 mg, 80%, 2 steps) as a yellowish amorphous solid. $[\alpha]_D^{20}$ = -12.4 (*c* 0.1, H_2O); **^{1}H NMR** (400 MHz, D_2O) δ 8.50 (d, *J* = 8.1 Hz, 1H), 5.99 (d, *J* = 8.2 Hz, 1H), 5.96 (d, *J* = 5.5 Hz, 1H), 4.54 – 4.33 (m, 7H), 3.95 – 3.82 (m, 2H), 3.74 – 3.22 (m, 8H), 3.47 (brs, 1H), 3.30 (s, 3H), 2.09 (s, 9H), 1.87 – 1.59 (m, 12H). **^{13}C NMR** (100 MHz, D_2O) δ 177.2, 175.4, 173.9, 173.8, 170.9, 170.2, 170.1, 169.8, 165.4, 152.9, 141.7, 101.4, 79.6, 74.9, 69.0, 63.4, 61.0, 57.3, 55.6, 53.8, 53.6, 53.4, 50.3, 47.9, 47.7,

47.6, 31.2, 28.0, 27.9, 22.7, 22.5, 22.2, 18.8. **HRMS** (ESI): calcd. for $C_{36}H_{58}KN_{10}O_{18}S^+$ $[M+K]^+$ 989.3283, found 989.3279.

Synthesis of compound 1b.

To a solution of compound **25b** (50 mg, 0.028 mmol, 1.0 equiv) in CH_3CN (3 mL), was added CAN (155 mg, 0.28 mmol, 10.0 equiv) in H_2O (1.5 mL) dropwise. The reaction was stirred at room temperature for 0.5 h, then diluted with EtOAc, washed with H_2O, saturated aqueous $NaHCO_3$ and brine, and dried over Na_2SO_4, concentrated *in vacuo* to afford crude **S5**, which was used in the next step without further purification.

S5 was dissolved in TFA (2 mL) and H_2O (10 μL), the reaction mixture was stirred at room temperature for 1 h, concentrated *in vacuo* to afford **S6**, which was used directly in the next step without further purification.

To a solution of **S6** in CH_3OH (3 mL) was added a solution of K_2CO_3 (7.7 mg, 0.056 mmol, 2.0 equiv) in H_2O (51 μL). The resulting solution was stirred for 1 h, before same amount of K_2CO_3 solution was added. The resulting reaction mixture was stirred until completely converted to **1b.** The pH of the reaction was adjusted to 7 with 0.5 M aqueous HCl. The mixture was concentrated *in vacuo*. The residue was washed with CH_2Cl_2 and then purified by Sephadex™ G-15 to afford **1b** (20 mg, 72%, 3 steps) as a yellowish amorphous solid. $[\alpha]_D^{20}$ = 23.0 (*c* 0.1, H_2O); **^{1}H NMR** (400 MHz, D_2O) δ 8.21 (d, *J* = 8.3 Hz, 1H), 6.23 (d, *J* = 8.2 Hz, 1H), 5.95 (d, *J* = 6.0 Hz, 1H), 4.49 (t, *J* = 6.4 Hz, 2H), 4.47 – 4.34 (m, 5H), 3.96 – 3.77 (m, 3H), 3.58 – 3.77 (m, 7H), 3.34 (s, 3H), 2.16 (brs, 9H), 1.93 – 1.59 (m, 12H). **^{13}C NMR** (100 MHz, D_2O) δ 176.1, 174.4, 174.4, 174.2, 171.7, 168.2, 156.2, 153.2, 138.8, 98.0, 79.9, 75.6, 69.7, 63.7, 61.8, 58.4, 56.3, 54.3, 54.1, 53.9, 51.7, 50.7, 48.0, 30.7, 30.2, 28.9, 28.8, 23.3, 23.1, 22.5, 20.1. **HRMS** (ESI): calcd. for $C_{37}H_{60}N_{12}NaO_{18}S^+$ $[M+Na]^+$ 1015.3761, found 1015.3758.

Synthesis of compound 1c.

1c was synthesized according to the procedures for the synthesis of **1a** from **25c**.

Analytical data for **1c**: (Yield = 75%, 2 steps), yellowish amorphous solid. **^{1}H NMR** (400 MHz, D_2O) δ 7.94 (d, *J* = 8.1 Hz, 1H), 6.00 (d, *J* = 8.1 Hz, 1H), 5.91 (d, *J* = 6.2 Hz, 1H), 4.55 – 4.23 (m, 7H), 3.98 – 3.81 (m, 2H), 3.59 (brs, 7H), 3.44 (brs, 1H), 3.32 (s, 3H), 2.06 (s, 9H), 1.88 – 1.55 (m, 12H). **^{13}C NMR** (100 MHz, D_2O) δ 177.7, 175.6, 174.1, 174.0, 173.9, 171.0, 167.8, 167.6, 164.7, 159.7, 152.6, 134.7, 101.8, 78.6, 74.6, 68.7, 62.1, 61.1, 57.2, 55.7, 54.0, 53.8, 53.5, 49.4, 48.3, 48.1, 31.4, 29.2, 28.1, 27.8, 22.8, 22.7, 22.5, 18.6. **HRMS** (ESI): calcd. for $C_{36}H_{59}N_{11}NaO_{17}S^+$ $[M+Na]^+$ 972.3703, found 972.3666.

Synthesis of compound 2.

To a solution of **13** (1.15 g, 0.95 mmol, 1.0 equiv) in CH_2Cl_2 (10 mL), TFA (5 mL) and H_2O (86 μL, 4.75 mmol, 5.0 equiv) were added at 0 ℃. After stirring for 18 h, the reaction mixture was diluted with toluene,

and concentrated *in vacuo* to give compound **2**, which was used directly in the next step without further purification.

Synthesis of compound 3a.

5a (78 mg, 0.20 mmol, 1.0 equiv) was dissolved in CH_3CN (3 mL), and IBX (112 mg, 0.40 mmol, 2.0 equiv) was added. The resulting suspension was heated to 55 ℃ and stirred vigorously. After 5 h (TLC monitoring), the reaction was cooled to room temperature and filtered through a medium glass frit. The filter cake was washed with EtOAc, and the combined filtrates were concentrated to yield crude aldehyde **23a** (77 mg), which was used directly in the next step without further purification.

To a solution of 24 (56 mg, 0.22 mmol, 1.1 equiv) in THF (2 mL) was added a 2 M hexane solution of LDA (110 μL, 0.22 mmol, 1.1 equiv) at -78 ℃. After stirring for 30 min, a solution of above crude 23a in THF (1 mL) was added, and the mixture was stirred for 2 h at the same temperature. The reaction was quenched by addition of saturated aqueous NH_4Cl, and 2 M aqueous HCl, extracted with ethyl acetate. The organic extracts were washed with brine and dried over Na_2SO_4. After evaporation of solvents, the residue was purified by silica gel flash chromatography to give 3a (82 mg, 86%, d.r. 4:1, 2 steps) as a white solid. R_f = 0.4 (silica gel, CH_2Cl_2 / CH_3OH = 15:1); m.p. 77–79 °C; $[\alpha]_D^{20}$ = -150.6 (*c* 0.3, $CHCl_3$); ^{1}H NMR (400 MHz, $CDCl_3$) δ 8.01 (d, *J* = 8.1 Hz, 1H), 6.16 (d, *J* = 9.3 Hz, 1H), 5.86 (d, *J* = 8.1 Hz, 1H), 4.68 (t, *J* = 9.9 Hz, 1H), 4.35 – 4.25 (m, 2H), 3.87 (d, *J* = 7.7 Hz, 1H), 3.78 (s, 3H), 3.50 (d, *J* = 8.2 Hz, 1H), 3.33 (s, 3H), 3.26 (s, 3H), 3.08 (s, 3H), 1.29 (s, 3H), 1.26 (s, 3H). ^{13}C NMR (100 MHz, $CDCl_3$) δ 175.2, 162.7, 151.9, 138.5, 103.1, 100.7, 100.5, 72.3, 70.1, 69.7, 57.6, 57.1, 52.6, 48.3, 48.0, 46.4, 28.2, 17.9, 17.7. HRMS (ESI): calcd. for $C_{19}H_{30}N_3O_9S^+$ $[M+H]^+$ 476.1697, found 476.1700.

Synthesis of compound 3b.

3b was synthesized according to the procedures for the synthesis of **3a** from **5b** by a two-step sequence.
Analytical data for **3b**: (83%, d.r. 3:1, 2 steps), white solid. R_f = 0.4 (silica gel, CH_2Cl_2 / CH_3OH = 15:1); **m.p.** 115–116 ℃; **$[\alpha]_D^{20}$** = -166.5 (*c* 0.2, $CHCl_3$); **Mixture of rotamers**. **^{1}H NMR** (400 MHz, $CDCl_3$) δ 7.76 (d, *J* = 8.3 Hz, 1H), 7.25 and 7.17 (d, *J* = 6.2 Hz, 2H), 6.90 – 6.82 (m, 2H), 6.80 and 6.65 (d, *J* = 8.3 Hz, 1H), 6.13 (d, *J* = 9.3 Hz, 1H), 5.50 and 5.23 (t, *J* = 5.5 Hz, 1H), 4.71 (t, *J* = 9.9 Hz, 1H), 4.43 – 4.35 (m, 2H), 4.31 – 4.25 (m, 2H), 3.85 (dd, *J* = 7.7 Hz, 1.1 Hz, 1H), 3.80 (s, 3H), 3.78 (s, 3H), 3.48 (d, *J* = 8.2 Hz, 1H), 3.40 and 3.35 (s, 3H), 3.26 (s, 3H), 3.10 (s, 3H), 1.30 (s, 3H), 1.26 (s, 3H). **^{13}C NMR** (100 MHz, $CDCl_3$) δ 175.2, 162.8, 159.1, 156.3, 151.3, 135.3, 131.1, 129.2, 114.2, 100.6, 100.5, 99.4, 71.8, 70.1, 69.8, 57.6, 57.2, 55.4, 52.6, 48.3, 48.1, 46.4, 44.2, 30.2, 17.9, 17.7. **HRMS** (ESI): calcd. for $C_{28}H_{40}N_5O_{10}S^+$ $[M+H]^+$ 638.2490, found 638.2497.

Synthesis of compound 3c.

3c was synthesized according to the procedures for the synthesis of **3a** from **5c** by a two-step sequence. Analytical data for **3c**: (75%, d.r. 3:1, 2 steps), colorless oil. R_f = 0.4 (silica gel, CH_2Cl_2 / CH_3OH = 15:1). **$[\alpha]_D^{20}$** = -82.5 (*c* 0.1, $CHCl_3$); **^{1}H NMR** (400 MHz, $CDCl_3$) δ 7.83 (d, *J* = 8.2 Hz, 1H), 7.77 (d, *J* = 7.5 Hz, 2H), 7.65 (d, *J* = 7.4 Hz, 2H), 7.40 (t, *J* = 7.3 Hz, 2H), 7.31 (dd, *J* = 13.6, 7.1 Hz, 2H), 6.16 (t, *J* = 9.6 Hz, 2H), 4.67 (t, *J* = 9.9 Hz, 1H), 4.61 – 4.46 (m, 2H), 4.37 – 4.27 (m, 3H), 3.87 (d, *J* = 7.5 Hz, 1H), 3.79 (s, 3H), 3.57 (d, *J* = 7.7 Hz, 1H), 3.45 (s, 3H), 3.26 (s, 3H), 3.10 (s, 3H), 1.30 (s, 3H), 1.27 (s, 3H). **^{13}C NMR** (100 MHz, $CDCl_3$) δ 174.8, 161.9, 157.8, 150.9, 144.1, 141.6, 141.5, 136.5, 127.8, 127.3, 127.2, 125.3, 120.1, 100.7, 100.5, 98.9, 72.2, 70.0, 69.7, 67.8, 57.6, 57.3, 52.6, 48.3, 48.1, 47.3, 46.5, 30.7, 17.9, 17.7. **HRMS** (ESI): calcd. for $C_{34}H_{41}N_4O_{10}S^+$ $[M+H]^+$ 697.2538, found 697.2535.

Synthesis of compound 4.

Compound **4** was synthesized according to the procedures for the synthesis of **12** from **12**.

Analytical data for **4**: (Yield = 87%), white foam. R_f = 0.4 (silica gel, CH_2Cl_2 / CH_3OH = 15:1); **$[\alpha]_D^{20}$** = 12.1 (*c* 0.1, $CHCl_3$); **^{1}H NMR** (400 MHz, $CDCl_3$) δ 8.04 (t, *J* = 8.4 Hz, 6H), 7.70 (d, *J* = 7.2 Hz, 2H), 7.64 – 7.51 (m, 6H), 7.51 – 7.37 (m, 6H), 7.34 (t, *J* = 6.7 Hz, 2H), 7.24 (t, *J* = 7.2 Hz, 1H), 6.11 (d, *J* = 7.0 Hz, 1H), 4.61 (brs, 1H), 4.40 (brs, 2H), 4.24 (d, *J* = 6.7 Hz, 2H), 4.13 (t, *J* = 7.0 Hz, 1H), 4.04 (brs, 2H), 3.86 (brs, 2H), 3.66 (brs, 2H), 2.21 – 1.85(m, 12H), 1.85 – 1.56 (m, 9H). **^{13}C NMR** (100 MHz, $CDCl_3$) δ 172.5, 172.1, 164.6, 156.5, 144.0, 143.8, 141.2, 134.6, 130.1, 129.0, 127.7, 126.5, 125.3, 119.9, 67.1, 54.0, 52.6, 47.1, 30.2, 29.5, 28.6, 23.6, 23.3, 20.3. **HRMS** (ESI): calcd. for $C_{57}H_{60}N_6NaO_{15}^+$ $[M+Na]^+$ 1091.4009, found 1091.4003.

Synthesis of compound 5a.

To a solution of **18** (105 mg, 0.28 mmol, 1.0 equiv) in DMF (3 mL) was added K_2CO_3 (58 mg, 0.42 mmol, 1.5 equiv) and methyl iodide (21 μL, 0.336 mmol, 1.2 equiv), and the solution was stirred for 2 h at room temperature. DMF was removed under reduced pressure. The residue was purified by silica gel flash chromatography to give **5a** (107 mg, 98%) as a white solid. R_f = 0.5 (silica gel, EtOAc / petroleum ether = 2:1); **m.p.** 194–195 ℃; **$[\alpha]_D^{20}$** = -206.7 (*c* 0.2, $CHCl_3$); **^{1}H NMR** (400 MHz, $CDCl_3$) δ 7.73 (d, *J* = 8.2 Hz, 1H), 6.18 (d, *J* = 9.5 Hz, 1H), 5.88 (d, *J* = 8.1 Hz, 1H), 4.44 (t, *J* = 10.0 Hz, 1H), 4.29 (dd, *J* = 10.4, 7.3 Hz, 1H), 3.94 (dd, *J* = 7.3, 4.5 Hz, 2H), 3.56 – 3.43 (m, 1H), 3.34 (s, 3H), 3.28 (s, 3H), 3.12 (s, 3H), 2.74 – 2.64 (m, 1H), 1.31 (s, 3H), 1.28 (s, 3H). **^{13}C NMR** (100 MHz, $CDCl_3$) δ 162.5, 151.7, 137.7, 103.2, 100.7, 100.5, 71.6, 70.4, 63.8, 57.4, 48.4, 48.1, 44.6, 28.3, 17.8, 17.7. **HRMS** (ESI): calcd. for $C_{16}H_{24}N_2NaO_7S^+$ $[M+Na]^+$ 411.1196, found 411.1196.

Synthesis of compound 5b.

To a solution of **22b** (166 mg, 0.25 mmol, 1.0 equiv) in THF (3 mL) was added TBAF·3H_2O (102 mg, 0.325 mmol, 1.3 equiv), and the mixture was stirred at room temperature for 2 h. The reaction mixture was

partitioned between EtOAc and H_2O, and the separated organic layer was washed with brine, dried over Na_2SO_4 and concentrated *in vacuo*. The residue was purified by silica gel flash chromatography to give **5b** (131 mg, 95%) as a colorless oil. R_f = 0.4 (silica gel, CH_2Cl_2 / CH_3OH = 30:1). $[\alpha]_D^{20}$ = -165.4 (*c* 0.2, $CHCl_3$); **Mixture of rotamers**. **^{1}H NMR** (400 MHz, $CDCl_3$) δ 7.52 – 7.41 (m, 1H), 7.24 and 7.15 (d, *J* = 8.4 Hz, 2H), 6.85 (d, *J* = 8.4 Hz, 2H), 6.80 and 6.63 (d, *J* = 8.2 Hz, 1H), 6.13 (d, *J* = 9.5 Hz, 1H), 5.59 and 5.38 (t, *J* = 5.1 Hz, 1H), 4.44 (t, *J* = 10.0 Hz, 1H), 4.38 (d, *J* = 5.7 Hz, 2H), 4.26 (dd, *J* = 10.1, 7.5 Hz, 1H), 3.99 – 3.86 (m, 2H), 3.78 (s, 3H), 3.45 (dd, *J* = 10.6, 6.5 Hz, 1H), 3.39 and 3.34 (s, 3H), 3.26 (s, 3H), 3.12 (s, 3H), 3.01 – 2.75 (m, 1H), 1.29 (s, 3H), 1.26 (s, 3H). **^{13}C NMR** (100 MHz, $CDCl_3$) δ 162.7, 159.0, 156.1, 151.1, 134.4, 131.0, 129.1, 114.1, 100.6, 100.5, 99.4, 71.1, 70.5, 63.8, 57.4, 55.4, 48.4, 48.2, 44.5, 44.2, 30.2, 17.8, 17.7. **HRMS** (ESI): calcd. for $C_{25}H_{34}N_4NaO_8S^+$ $[M+Na]^+$ 573.1990, found 573.1994.

Synthesis of compound 5c.

To a solution of **22c** (181 mg, 0.25 mmol, 1.0 equiv) in THF (4 mL) was added HF·pyridine (190 mg, 65%, 1.25 mmol, 5.0 equiv). The reaction mixture was stirred at room temperature for 36 h. When TLC showing starting material disappeared, quenched with saturated aqueous NH_4Cl (20 mL), extracted with EtOAc (3 × 20 mL), the combined organic phases were washed with brine and dried over Na_2SO_4, concentrated *in vacuo*. The residue was purified by silica gel flash chromatography to give **5c** (145 mg, 95%) as a colorless oil. R_f = 0.4 (silica gel, EtOAc / petroleum ether = 2:1). $[\alpha]_D^{20}$ = -132.3 (*c* 0.1, $CHCl_3$); **^{1}H NMR** (400 MHz, $CDCl_3$) δ 7.76 (d, *J* = 7.4 Hz, 2H), 7.65 (d, *J* = 7.1 Hz, 2H), 7.56 (d, *J* = 7.2 Hz, 1H), 7.40 (t, *J* = 7.3 Hz, 2H), 7.35 – 7.27 (m, 1H), 6.34 (d, *J* = 7.1 Hz, 1H), 6.16 (d, *J* = 9.3 Hz, 1H), 4.51 (d, *J* = 6.1 Hz, 2H), 4.45 (t, *J* =10.4 Hz, 1H), 4.35 – 4.25 (m, 2H), 3.94 (d, *J* = 3.5 Hz, 2H), 3.48 (s, 4H), 3.27 (s, 1H), 3.13 (s, 1H), 1.31 (s, 3H), 1.28 (s, 3H). **^{13}C NMR** (100 MHz, $CDCl_3$) δ 161.8, 157.8, 150.7, 144.0, 141.5, 141.4, 135.8, 127.8, 127.2, 127.2, 125.3, 120.1, 100.7, 100.5, 99.0, 71.5, 70.4, 68.0, 63.7, 57.6, 48.4, 48.3, 47.2, 44.7, 30.8, 17.8, 17.7. **HRMS** (ESI): calcd. for $C_{31}H_{35}N_3NaO_8S^+$ $[M+Na]^+$ 632.2037, found 632.2033.

Synthesis of compound 6.

A solution of BPO (2.90 g, 12.0 mmol, 1.2 equiv) in CH_2Cl_2 (100 mL) was added quickly to a solution of the amine **8** (2.88 g, 10.0 mmol, 1.0 equiv) in pH 10.5 buffer (100 mL) at room temperature. The resulting biphasic mixture was stirred for 3 h at room temperature. Then, a solution of acetyl chloride (0.72 mL, 10.0 mmol, 1.0 equiv) in CH_2Cl_2 (12 mL) was added to the reaction mixture. After the acylation reaction was complete, the water layer was extracted with CH_2Cl_2 (50 mL × 3). The combined organic layers were washed with brine, dried over Na_2SO_4, and concentrated *in vacuo*. The resulting residue was purified with silica gel flash chromatography to afford **6** (3.6 g, 80%) as a colorless oil. R_f = 0.5 (silica gel, petroleum ether / EtOAc = 3:1); $[\alpha]_D^{20}$ = 4.3 (*c* 0.6, $CHCl_3$); **Mixture of rotamers**. **^{1}H NMR** (400 MHz, $CDCl_3$) δ 8.11 – 8.06 (m, 2H), 7.67 (t, *J*=7.4, 1H), 7.51 (t, *J*=7.7, 2H), 5.20 – 4.65 (m, 1H), 4.22 – 3.96 (m, 1H), 3.83 (t, *J*=6.3, 2H), 2.06 (s,

3H), 1.95 – 1.59 (m, 4H), 1.43 (s, 9H), 1.41 (s, 9H). ^{13}**C NMR** (100 MHz, $CDCl_3$) δ 171.7, 164.5, 155.5, 134.6, 130.1, 129.0, 126.7, 82.0, 79.6, 53.7, 47.8, 30.2, 28.4, 28.0, 23.2, 20.4. **HRMS** (ESI): calcd. for $C_{23}H_{34}N_2NaO_7^+$ $[M+Na]^+$ 473.2258, found 473.2259.

Synthesis of compound 7.

To a solution of diacetal **S2** (1.12 g, 3.83 mmol, 1.0 equiv) in CH_2Cl_2 (15 mL), was added DIBAL-H (1.5 M in toluene, 5.4 mL, 8.04 mmol, 2.1 equiv) at 0 ℃. After 2 h, the reaction was quenched with saturated sodium potassium tartrate (10 mL) at 0 ℃. The organic layer was collected and the aqueous layer was extracted with CH_2Cl_2 (20 mL × 3). The combined organic layers were washed with brine, dried over Na_2SO_4 and concentrated *in vacuo*. The residue was purified with silica gel flash chromatography to afford **7** (991 mg, 98%) as a colorless oil. R_f = 0.4 (silica gel, petroleum ether / EtOAc = 5:1); $[\alpha]_D^{20}$ = -101.3 (*c* 0.4, $CHCl_3$); ^{1}**H NMR** (400 MHz, $CDCl_3$) δ 4.22 (dd, *J* = 17.4, 10.4 Hz, 1H), 4.08 (dd, *J* = 9.8, 7.9 Hz, 1H), 3.92 (t, *J* = 11.3 Hz, 1H), 3.52 (td, *J* = 11.4, 4.3 Hz, 1H), 3.43–3.34 (m, 1H), 3.26 (s, 6H), 2.84 (dd, *J* = 10.0 Hz, 2.5 Hz, 1H), 2.75 (t, *J* = 9.0 Hz, 1H), 2.69 (t, *J* = 10.5 Hz, 1H), 1.30 (s, 6H). ^{13}**C NMR** (100 MHz, $CDCl_3$) δ 100.4, 100.2, 74.6, 69.0, 65.3, 48.3, 48.2, 41.7, 27.3, 18.0. **HRMS** (ESI): calcd. for $C_{11}H_{20}NaO_5S^+$ $[M+Na]^+$ 287.0924, found 287.0920.

Synthesis of compound 11.

Compound **6** (3.38 g, 7.50 mmol, 1.0 equiv) was dissolved in TFA (15 mL) and H_2O (0.67 mL, 37.5 mmol, 5.0 equiv) and stirred at room temperature for 12 h. The reaction mixture was concentrated and the residue was dissolved in dry CH_3OH (30 mL) and evaporated under reduced pressure. This procedure was repeated three times to produce **10**, which was used in the next step without further purification.

To a solution of **10** (919 mg, 2.25 mmol, 1.0 equiv) in DMF (25 mL) was stirred at -15 ℃, to which was added Et_3N (0.94 mL, 6.75 mmol, 3.0 equiv) and Fmoc-OSu (911 mg, 2.70 mmol, 1.2 equiv). The reaction mixture was stirred overnight, and then acidified to pH 5 with 1 M aqueous HCl. Solvent was removed under reduced pressure. The residue was diluted with EtOAc, washed with 1 M aqueous HCl and brine, dried over Na_2SO_4, and concentrated *in vacuo*. Column chromatography using CH_2Cl_2 / CH_3OH as eluent yielded **11** (1.06 g, 91%, 2 steps) as a white foam. R_f = 0.5 (silica gel, CH_2Cl_2 / CH_3OH = 15:1); $[\alpha]_D^{20}$ = -2.4 (*c* 0.7, $CHCl_3$); ^{1}**H NMR** (400 MHz, $CDCl_3$) δ 8.04 (d, *J* = 7.1 Hz, 2H), 7.72 (d, *J* = 6.8 Hz, 2H), 7.65 – 7.51 (m, 3H), 7.49 – 7.40 (m, 2H), 7.39 – 7.31 (m, 2H), 7.30 – 7.18 (m, 2H), 6.18 – 5.71 (m, 1H), 4.53 – 4.20 (m, 3H), 4.10 (brs, 1H), 4.00 – 3.64 (m, 2H), 2.15 – 1.53 (m, 7H). ^{13}**C NMR** (100 MHz, $CDCl_3$) δ 175.2, 164.6, 156.4, 144.0, 143.8, 141.4, 134.8, 130.2, 129.1, 127.8, 127.2, 126.5, 125.3, 120.0, 67.2, 53.5, 47.2, 29.5, 23.4, 20.4. **HRMS** (ESI): calcd. for $C_{29}H_{28}N_2NaO_7^+$ $[M+Na]^+$ 539.1789, found 539.1796.

Synthesis of compound 12.

Thionyl chloride (145 μL, 2.0 mmol, 1.0 equiv) was added to a solution of 1*H*-benzotriazole (952 mg, 8.0 mmol, 4.0 equiv) in THF (10 mL), and the reaction mixture was stirred for 20 min at room temperature. The reaction mixture was cooled in an ice-bath, and **11** (1.03 g, 2.0 mmol, 1.0 equiv) dissolved in THF (5 mL) was added dropwise. After stirring for 0.5 h at room temperature, the white precipitate formed during the reaction was filtered off, and the filtrate was concentrated under reduced pressure. The residue was diluted with EtOAc, and the white precipitate formed was filtered off again. Removing solvents under reduced pressure gave the crude active amide which was used in the following reaction.

The crude active amide obtained from above was added to a solution of **10** (898 mg, 2.2 mmol, 1.1 equiv) with Et_3N (0.86 mL, 6.2 mmol, 3.1 equiv) in CH_3CN–H_2O (20 mL / 8 mL) at -15 ℃. The reaction mixture was stirred at room temperature for 2 h. CH_3CN in the solution was removed under reduced pressure, and EtOAc was added. The organic solution was washed with 0.5 M aqueous HCl and brine, dried over Na_2SO_4, and concentrated *in vacuo*. Column chromatography using CH_2Cl_2 / CH_3OH as eluent yielded **12** (1.41 g, 89%) as a white foam. R_f = 0.4 (silica gel, CH_2Cl_2 / CH_3OH = 15:1); $[\alpha]_D^{20}$ = 29.8 (*c* 0.1, $CHCl_3$); **^{1}H NMR** (400 MHz, $CDCl_3$) δ 8.05 (d, *J* = 7.1 Hz, 4H), 7.72 (d, *J* = 7.0 Hz, 2H), 7.68 – 7.53 (m, 4H), 7.52 – 7.40 (m, 5H), 7.40 – 7.30 (m, 2H), 7.26 (t, *J* = 7.2 Hz, 2H), 6.05 (d, *J* = 7.8 Hz, 1H), 4.55 (brs, 1H), 4.47 (brs, 1H), 4.27 (d, *J* = 5.0 Hz, 2H), 4.15 (t, *J* = 7.0 Hz, 2H), 3.82 (brs, 2H), 3.66 (d, *J* = 12.5 Hz, 1H), 2.16 – 1.90 (m, 8H), 1.87 – 1.63 (m, 6H). **^{13}C NMR** (100 MHz, $CDCl_3$) δ 173.9, 172.6, 164.5, 156.5, 144.0, 143.8, 141.3, 134.7, 130.2, 129.0, 127.8, 127.2, 126.5, 125.3, 120.0, 67.2, 53.6, 52.2, 47.2, 30.4, 28.8, 23.6, 23.2, 20.3. **HRMS** (ESI): calcd. for $C_{43}H_{44}N_4NaO_{11}^+$ $[M+Na]^+$ 815.2899, found 815.2899.

Synthesis of compound 13.

To a solution of compound **4** (1.28 g, 1.20 mmol, 1.0 equiv), H-Ser(*t*Bu)-O*t*Bu·HCl (260 mg, 1.32 mmol, 1.1 equiv) and HATU (685 mg, 1.80 mmol, 1.5 equiv) in DMF (10 mL), was added DIPEA (398 μL, 2.40 mmol, 2.0 equiv) dropwise at -15 ℃. The reaction mixture was stirred at -15 ℃ for 2 h, and then acidified to pH 5 with 1 M aqueous HCl. The solvent was removed under reduced pressure, and EtOAc was added. The organic solution was washed with 0.5 M aqueous HCl, saturated aqueous $NaHCO_3$, brine, dried over Na_2SO_4, and concentrated *in vacuo*. Column chromatography using CH_2Cl_2/CH_3OH as eluent yielded **13** (1.38 g, 95%) as a white foam. R_f = 0.4 (silica gel, CH_2Cl_2 / CH_3OH = 20:1); $[\alpha]_D^{20}$ = -12.2 (*c* 0.1, $CHCl_3$); **^{1}H NMR** (400 MHz, $CDCl_3$) δ 8.07 (d, *J* = 7.5 Hz, 6H), 7.73 (d, *J* = 7.5 Hz, 2H), 7.69 – 7.56 (m, 5H), 7.54 – 7.44 (m, 6H), 7.37 (t, *J* = 7.3 Hz, 2H), 7.31 – 7.24 (m, 3H), 7.20 (d, *J* = 7.7 Hz, 1H), 5.97 (s, 1H), 4.52 (brs, 1H), 4.49 – 4.42 (m, 2H), 4.41 – 4.23 (m, 3H), 4.18 (t, *J* = 6.9 Hz, 1H), 4.15 – 4.05 (m, 1H), 3.98 (brs, 2H), 3.93 – 3.80 (m, 3H), 3.80 – 3.60 (m, 3H), 2.12 – 1.89 (m, 12H), 1.73 (brs, 9H), 1.45 (s, 9H). **^{13}C NMR** (100 MHz, $CDCl_3$) δ 173.3, 171.9, 171.3, 169.2, 164.8, 164.7, 164.6, 157.0, 143.9, 143.8, 141.4, 134.8, 130.2, 129.1, 127.8, 127.2, 126.6,

125.3, 120.1, 82.2, 67.3, 63.2, 55.7, 54.7, 53.7, 52.8, 47.2, 29.7, 28.5, 28.1, 24.0, 23.8, 23.7, 20.5, 20.4. **HRMS** (ESI): calcd. for $C_{64}H_{73}N_7NaO_{17}^+$ $[M+Na]^+$ 1234.4955, found 1234.4961.

Synthesis of compound 14.

To an ice-cold solution of triphenylphosphine (15.3 g, 58.5 mmol, 2.2 equiv) in THF (100 mL), diisopropyl azodicarboxylate (11.6 mL, 58.5 mmol, 2.2 equiv) was added over 5 min. After stirring for 30 min, compound **9** (5.00 g, 26.6 mmol, 1.0 equiv) was added, and stirring was continued for 10 min. To the resulting yellow suspension, a solution of thioacetic acid (4.1 mL, 58.5 mmol, 2.2 equiv) in THF (20 mL) was added dropwise and stirring was continued for another 4 h at 0 ℃. During this time the yellow suspension cleared, and an orange solution was obtained. At the end of the reaction the solvent was removed under reduced pressure, and the resulting yellowish residue was purified by silica gel flash chromatography to produce **S1** (89%, 5.82 g) as a white solid. R_f = 0.6 (silica gel, petroleum ether / EtOAc = 2:1); **m.p.** 67–69 ℃; $[\alpha]_D^{20}$ = -81.7 (*c* 0.3, $CHCl_3$); **^{1}H NMR** (400 MHz, $CDCl_3$) δ 4.81 (s, 2H), 4.57 – 4.48 (m, 1H), 3.34 (dd, *J* = 13.9, 7.4 Hz, 1H), 3.26 (dd, *J* = 13.9, 6.7 Hz, 1H), 2.38 (s, 3H), 1.48 (s, 3H), 1.40 (s, 3H). **^{13}C NMR** (100 MHz, $CDCl_3$) δ 195.1, 173.3, 114.4, 77.5, 76.4, 76.3, 30.6, 27.9 26.8, 26.0. **HRMS** (ESI): calcd. for $C_{10}H_{14}NaO_5S^+$ $[M+Na]^+$ 269.0454, found 269.0446.

To a solution of **S1** (5.0 g, 20.3 mmol, 1.0 equiv) in CH_2Cl_2 (40 mL), TFA (8 mL) and H_2O (1.8 mL) were added at room temperature. After stirring for 5 h, the reaction mixture was diluted with toluene, and concentrated *in vacuo*. Colum chromatography using CH_2Cl_2/CH_3OH as eluent yielded the title compound **14** (3.76 g, 90%) as a white solid. R_f = 0.4 (silica gel, CH_2Cl_2 / CH_3OH = 15:1); **m.p.** 115–118 ℃; $[\alpha]_D^{20}$ = -40.4 (*c* 0.3, CH_3OH); **^{1}H NMR** (400 MHz, CD_3OD) δ 4.50 (d, *J* = 4.6 Hz, 1H), 4.41 (td, *J* = 7.0, 2.8 Hz, 1H), 4.33 (dd, *J* = 4.6, 2.9 Hz, 1H), 3.33–3.26 (m, 1H), 3.21 (dd, *J* = 13.8, 6.8 Hz, 1H), 2.36 (s, 3H). **^{13}C NMR** (100 MHz, CD_3OD) δ 196.6, 177.7, 80.1, 72.2, 71.0, 30.4, 28.2. **HRMS** (ESI): calcd. for $C_7H_{10}NaO_5S^+$ $[M+Na]^+$ 229.0141, found 229.0137.

Synthesis of compound 15.

To a solution of **14** (3.55 g, 17.2 mmol, 1.0 equiv) and TsCl (3.94 g, 20.6 mmol, 1.2 equiv) in CH_3CN (35 mL) was added a solution of DABCO (2.12 g, 18.9 mmol, 1.1 equiv) in CH_3CN (35 mL) dropwise over 30 min. The resultant solution was stirred at room temperature for further 30 min, and then concentrated *in vacuo*. The residue was purified by silica gel flash chromatography to give **15** (5.39 g, 87%) as a white solid. R_f = 0.5 (silica gel, petroleum ether / EtOAc = 2:1); **m.p.** 140–142 ℃; $[\alpha]_D^{20}$ = -69.2 (*c* 0.3, $CHCl_3$); **^{1}H NMR** (400 MHz, $CDCl_3$) δ 7.86 (d, *J* = 8.2 Hz, 2H), 7.37 (d, *J* = 8.1 Hz, 2H), 5.07 (d, *J* = 4.3 Hz, 1H), 4.71–4.62 (m, 1H), 4.44 (td, *J* = 7.0, 2.5 Hz, 1H), 3.46 (d, *J* = 2.0 Hz, 1H), 3.29 (d, *J* = 7.1 Hz, 2H), 2.45 (s, 3H), 2.37 (s, 3H). **^{13}C NMR** (100 MHz, $CDCl_3$) δ 196.3, 168.5, 146.3, 131.7, 130.2, 128.4, 78.6, 75.5, 68.9, 30.6, 26.8, 21.9. **HRMS**

(ESI): calcd. for $C_{14}H_{16}NaO_7S_2^+$ $[M+Na]^+$ 383.0230, found 383.0227.

Synthesis of compound 16.

To a solution of **15** (3.85 g, 10.7 mmol, 1.0 equiv) in CH_3OH (15 mL) was added K_2CO_3 (2.22 g, 16.0 mmol, 1.5 equiv) at -30 ℃. After being stirred for 3 h, the resultant solution was concentrated *in vacuo*. The residue was purified by silica gel flash chromatography to give **16** (1.54 g, 81%) as a white solid. R_f = 0.3 (silica gel, petroleum ether / EtOAc = 1:3); **m.p.** 86–88 ℃; $[\alpha]_D^{20}$ = 114.6 (*c* 0.3, $CHCl_3$); **^{1}H NMR** (400 MHz, CD_3OD) δ 4.38 (d, *J* = 3.1 Hz, 1H), 4.30 (t, *J* = 4.3 Hz, 1H), 4.22 (d, *J* = 4.7 Hz, 1H), 3.73 (s, 3H), 3.27 (dd, *J* = 11.0, 4.9 Hz, 1H), 2.72 (d, *J* = 11.0 Hz, 1H). **^{13}C NMR** (100 MHz, CD_3OD) δ 173.1, 80.2, 78.3, 52.9, 52.8, 36.3. **HRMS** (ESI): calcd. for $C_6H_{10}NaO_4S^+$ $[M+Na]^+$ 201.0192, found 201.0188.

Synthesis of compound 17.

To a solution of **7** (903 mg, 3.42 mmol, 1.0 equiv) in CH_2Cl_2 (10 mL) was added *m*-CPBA (590 mg, 3.42 mmol, 1.0 equiv) at 0 ℃, and the mixture was stirred at the same temperature for 30 min. The reaction was quenched by addition of saturated aqueous $Na_2S_2O_3$ and saturated aqueous $NaHCO_3$, and partitioned between EtOAc and H_2O. The separated organic layer was washed with saturated aqueous $NaHCO_3$ and brine. The combined organic phases were dried over Na_2SO_4, concentrated *in vacuo*. The residue was purified with silica gel flash chromatography to afford **17** (910 mg, 95%) as a white solid. R_f = 0.3 (silica gel, petroleum ether / EtOAc = 1:2); **m.p.** 69–71 ℃; $[\alpha]_D^{20}$ = -59.6 (*c* 0.2, $CHCl_3$); **^{1}H NM**R (400 MHz, $CDCl_3$) δ 4.83 (ddd, *J* = 12.3, 10.3, 6.7 Hz, 1H), 4.33 – 4.22 (m, 1H), 4.22 – 4.13 (m, 1H), 3.84 (dd, *J* = 10.1, 8.7 Hz, 1H), 3.81 – 3.73 (m, 1H), 3.28 (s, 3H), 3.23 (s, 3H), 3.09 – 2.94 (m, 2H), 2.89 – 2.73 (t, *J* = 12.2, 1H), 1.32 (s, 3H), 1.30 (s, 3H). **^{13}C NMR** (100 MHz, $CDCl_3$) δ 100.7, 100.6, 71.0, 68.5, 58.6, 57.6, 51.3, 48.4, 48.3, 17.9, 17.8. **HRMS** (ESI): calcd. for $C_{11}H_{20}NaO_6S^+$ $[M+Na]^+$ 303.0873, found 303.0871.

Synthesis of compound 18.

Uracil (672 mg, 6.0 mmol, 2.0 equiv) was dissolved in toluene (20 mL). DIPEA (2.0 mL, 12.0 mmol, 4.0 equiv) and TMSOTf (4.3 mL, 24.0 mmol, 8.0 equiv) were added dropwise and vigorous stirring at room temperature. The resulting suspension was stirred for 30 min to give a biphasic system, which was homogenized by adding CH_2Cl_2 (20 mL). A solution of the sulfoxide **17** (841 mg, 3.0 mmol, 1.0 equiv) in CH_2Cl_2 (20 mL) was added dropwise and the reaction mixture was stirred for another 30 min at room temperature before the addition of DIPEA (2.0 mL, 12.0 mmol, 4.0 equiv) in toluene (10 mL). Stirring was continued for 2 h and then chilled water and saturated aqueous $NaHCO_3$ were added. After 15 min, the reaction mixture was filtered through a short pad of celite, which was further washed with CH_2Cl_2. The aqueous phase was extracted with CH_2Cl_2, and the combined organic phases were dried over Na_2SO_4, concentrated *in vacuo*. The residue was purified with silica gel flash chromatography to afford **18** (483 mg,

43%) as a white solid. R_f = 0.4 (silica gel, petroleum ether / EtOAc = 1:2); and ***epi*-18** (483 mg, 43%) as a white solid, R_f = 0.3 (silica gel, petroleum ether / EtOAc = 1:2).

18: **m.p.** 232–233 ℃; **$[\alpha]_D^{20}$** = -179.7 (*c* 0.1, $CHCl_3$); **^{1}H NMR** (400 MHz, CD_3OD) δ 8.22 (d, *J* = 8.1 Hz, 1H), 6.07 (d, *J* = 9.3 Hz, 1H), 5.81 (d, *J* = 8.1 Hz, 1H), 4.54 (t, *J* = 9.9 Hz, 1H), 4.21 (dd, *J* = 10.2, 7.6 Hz, 1H), 3.95 (dd, *J* = 11.7, 5.1 Hz, 1H), 3.85 (dd, *J* = 11.7, 3.7 Hz, 1H), 3.50 – 3.44 (m, 1H), 3.28 (s, 3H), 3.13 (s, 3H), 1.30 (s, 3H), 1.27 (s, 3H). **^{13}C NMR** (100 MHz, CD_3OD) δ 165.5, 152.6, 142.6, 104.0, 101.7, 101.5, 73.5, 71.2, 62.5, 57.8, 48.4, 48.2, 47.9, 18.0, 18.0. **HRMS** (ESI): calcd. for $C_{15}H_{22}N_2NaO_7S^+$ [M+Na]$^+$ 397.1040, found 397.1034.

***epi*-18**: **m.p**. 103–105 ℃; **$[\alpha]_D^{20}$** = 17.5 (*c* 0.2, $CHCl_3$); **^{1}H NMR** (400 MHz, $CDCl_3$) δ 9.59 (brs, 1H), 7.84 (d, *J* = 8.2 Hz, 1H), 6.07 (d, *J* = 6.8 Hz, 1H), 5.76 (d, *J* = 8.2 Hz, 1H), 4.45 (dd, *J* = 11.3, 6.9 Hz, 1H), 4.33 (dd, *J* = 11.2, 7.4 Hz, 1H), 4.00 – 3.91 (m, 1H), 3.70 – 3.55 (m, 2H), 3.24 (s, 6H), 2.87 (dd, *J* = 9.2, 3.5 Hz, 1H), 1.29 (s, 3H), 1.22 (s, 3H). **^{13}C NMR** (100 MHz, $CDCl_3$) δ 163.6, 151.4, 141.5, 102.1, 101.0, 100.7, 71.2, 69.9, 63.8, 56.8, 48.5, 43.0, 17.7, 17.7. **HRMS** (ESI): calcd. for $C_{15}H_{22}N_2NaO_7S^+$ [M+Na]$^+$ 397.1040, found 397.1037.

Synthesis of compound 19.

To a solution of compound **18** (355 mg, 0.95 mmol, 1.0 equiv) in CH_3CN (6 mL), were added imidazole (142 mg, 2.09 mmol, 2.2 equiv), TBSCl (315 mg, 2.09 mmol, 2.2 equiv) and DMAP (12 mg, 0.095 mmol, 0.1 equiv) at 0 ℃. The resultant solution was stirred at room temperature for 2 h. The reaction was quenched with H_2O (10 mL) and extracted with EtOAc. The combined organic layers were washed with brine, dried over Na_2SO_4, concentrated *in vacuo*. The residue was purified with silica gel flash chromatography to afford **19** (454 mg, 98%) as a white foam. R_f = 0.4 (silica gel, petroleum ether / EtOAc = 2:1); **$[\alpha]_D^{20}$** = -108.1 (*c* 0.3, $CHCl_3$); **^{1}H NMR** (400 MHz, $CDCl_3$) δ 8.69 (brs, 1H), 8.22 (d, *J* = 8.2 Hz, 1H), 6.15 (d, *J* = 8.9 Hz, 1H), 5.77 (d, *J* = 8.1 Hz, 1H), 4.49 (t, *J* = 9.7 Hz, 1H), 4.19 (dd, *J* = 10.2, 7.8 Hz, 1H), 4.14 (dd, *J* = 10.9, 2.1 Hz, 1H), 3.76 (dd, *J* = 10.9, 2.6 Hz, 1H), 3.40 – 3.34 (m, 1H), 3.24 (s, 3H), 3.06 (s, 3H), 1.27 (s, 6H), 0.96 (s, 9H), 0.17 (s, 3H), 0.15 (s, 3H). **^{13}C NMR** (100 MHz, $CDCl_3$) δ 163.3, 151.0, 141.3, 103.3, 100.6, 100.4, 73.5, 69.7, 62.5, 56.9, 48.1, 47.8, 46.6, 26.2, 18.9, 17.9, 17.7, -5.1, -5.5. **HRMS** (ESI): calcd. for $C_{21}H_{36}N_2NaO_7SSi^+$ [M+Na]$^+$ 511.1905, found 511.1910.

Synthesis of compound 20.

To a solution of **S3** (253 mg, 0.52 mmol, 1.0 equiv) in *N*, *N*-dimethylacetamide (DMAc) (5 mL) was added methyl iodide (97 µL, 1.56 mmol, 3 equiv). The reaction was stirred at room temperature for 3.5 h. The solvent was removed under vacuum. The residue was purified by silica gel flash chromatography to give **20** (250 mg, 96%) as a colorless oil. R_f = 0.3 (silica gel, CH_2Cl_2 / CH_3OH =15:1); **$[\alpha]_D^{20}$** = -73.1 (*c* 0.2, $CHCl_3$);

^{1}**H NMR** (400 MHz, $CDCl_3$) δ 8.23 (d, *J* = 7.6 Hz, 1H), 6.67 (d, *J* = 7.5 Hz, 1H), 6.14 (d, *J* = 8.9 Hz, 1H), 4.49 (t, *J* = 9.6 Hz, 1H), 4.25 – 4.14 (m, 1H), 4.10 (d, *J* = 10.6 Hz, 1H), 3.74 (d, *J* = 10.8 Hz, 1H), 3.68 (s, 3H), 3.37 (d, *J* = 7.2 Hz, 1H), 3.22 (s, 3H), 3.04 (s, 3H), 1.25 (s, 6H), 0.94 (s, 9H), 0.15 (s, 3H), 0.13 (s, 3H). ^{13}**C NMR** (100 MHz, $CDCl_3$) δ 158.5, 148.6, 140.4, 100.6, 100.4, 97.0, 73.4, 69.6, 62.3, 58.7, 48.1, 48.0, 46.8, 32.5, 26.1, 18.8, 17.8, 17.6, -5.1, -5.5. **HRMS** (ESI): calcd. for $C_{22}H_{40}N_3O_6SSi^+$ $[M+H]^+$ 502.2402, found 502.2403.

Synthesis of compound 22b.

To a solution of **20** (140 mg, 0.28 mmol, 1.0 equiv) in THF (4 mL) was added Et_3N (39 µL, 0.28 mmol, 1.0 equiv) and compound **21** (130 mg, 0.56 mmol, 2.0 equiv), the solution was heated to 70 °C. The reaction was monitored by TLC analysis and upon consumption of starting material was cooled to room temperature. The solvent was removed under reduced pressure. The residue was purified by silica gel flash chromatography to give **22b** (179 mg, 96%) as a white foam. R_f = 0.5 (silica gel, petroleum ether / EtOAc = 2:1); $[\alpha]_D^{20}$ = -145.4 (*c* 0.1, $CHCl_3$); **Mixture of rotamers**. ^{1}**H NMR** (400 MHz, $CDCl_3$) δ 7.82 (d, *J* = 8.3 Hz, 1H), 7.25 and 7.17 (d, *J* = 6.4 Hz, 2H), 6.86 (d, *J* = 8.3 Hz, 2H), 6.81 and 6.61 (d, *J* = 8.3 Hz, 1H), 6.20 (d, *J* = 9.1 Hz, 1H), 5.53 and 5.26 (brs, 1H), 4.51 (t, *J* = 9.7 Hz, 1H), 4.40 (d, *J* = 5.7 Hz, 2H), 4.22 – 4.15 (m, 1H), 4.09 (dd, *J* = 10.8, 2.4 Hz, 1H), 3.82 – 3.73 (m, 4H), 3.42 –3.32 (m, 4H), 3.24 (s, 3H), 3.07 (s, 3H), 1.27 (s, 3H), 1.26 (s, 3H), 0.95 (s, 9H), 0.16 (s, 3H), 0.14 (s, 3H). ^{13}**C NMR** (100 MHz, $CDCl_3$) δ 162.7, 159.1, 156.5, 151.3, 135.6, 131.1, 129.2, 114.2, 100.6, 100.4, 98.9, 72.7, 69.8, 62.6, 57.6, 55.4, 48.1, 46.5, 44.2, 30.3, 26.2, 18.9, 17.9, 17.7, -5.0, -5.3. **HRMS** (ESI): calcd. for $C_{31}H_{48}N_4NaO_8SSi^+$ $[M+Na]^+$ 687.2854, found 687.2860.

Synthesis of compound 22c.

To a solution of **20** (140 mg, 0.28 mmol, 1.0 equiv) in THF-H_2O (2 mL/2 mL) was added $NaHCO_3$ (94 mg, 1.12 mmol, 4.0 equiv), followed by Fmoc-Cl (145 mg, 0.56 mmol, 2.0 equiv). The reaction mixture was stirred at room temperature overnight, when TLC showing starting material disappeared, diluted with water (3 mL), extracted with EtOAc. The combined organic phases were washed with brine and dried over Na_2SO_4, concentrated *in vacuo*. The residue was purified by silica gel flash chromatography to give **22c** (182 mg, 90%) as a light brown oil. R_f = 0.5 (silica gel, EtOAc / petroleum ether = 5:1). $[\alpha]_D^{20}$ = -169.4 (*c* 0.1, $CHCl_3$); ^{1}**H NMR** (400 MHz, $CDCl_3$) δ 7.97 (d, *J* = 7.9 Hz, 1H), 7.77 (d, *J* = 7.4 Hz, 2H), 7.67 (d, *J* = 7.1 Hz, 2H), 7.40 (t, *J* = 7.3 Hz, 2H), 7.31 (t, *J* = 7.0 Hz, 2H), 6.56 (d, *J* = 7.9 Hz, 1H), 6.23 (d, *J* = 9.0 Hz, 1H), 4.52 (t, *J* = 9.6 Hz, 1H), 4.46 (d, *J* = 7.0 Hz, 2H), 4.34 (t, *J* = 6.6 Hz, 1H), 4.25 – 4.17 (m, 1H), 4.11 (dd, *J* = 9.2 Hz, 2.2 Hz, 1H), 3.78 (dd, *J* = 10.7 Hz, 2.0 Hz, 1H), 3.52 (s, 3H), 3.41 – 3.35 (m, 1H), 3.25 (s, 3H), 3.09 (s, 3H), 1.28 (s, 6H), 0.95 (s, 9H), 0.16 (s, 3H), 0.14 (s, 3H). ^{13}**C NMR** (100 MHz, $CDCl_3$) δ 161.9, 158.7, 150.8, 144.2, 144.1, 141.4, 141.4, 137.2, 127.8, 127.2, 127.2, 125.5, 125.4, 120.1, 100.6, 100.5, 98.6, 73.0, 69.8, 68.2, 62.6, 57.9, 48.1, 47.1, 46.6, 30.9, 26.2, 18.9, 17.9, 17.7, -5.0, -5.4. **HRMS** (ESI): calcd. for $C_{37}H_{49}N_3NaO_8SSi^+$ $[M+Na]^+$

746.2902, found 746.2893.

Synthesis of compound 25a.

To a solution of compound **3a** (30 mg, 0.063 mmol, 1.0 equiv), compound **2** (73 mg, 0.063 mmol, 1.0 equiv) and HATU (36 mg, 0.095 mmol, 1.5 equiv) in DMF (4 mL), were added DIPEA (22 μL, 0.126 mmol, 2.0 equiv) dropwise at -15 ℃. The reaction was stirred for 2 h. After adding 2 M aqueous HCl into the mixture, the solvent was removed under reduced pressure. The residue was diluted with EtOAc, washed with 0.5 M aqueous HCl, saturated aqueous $NaHCO_3$, brine and dried over Na_2SO_4, concentration *in vacuo*. The residue was purified by silica gel flash chromatography to produce **25a** (94 mg, 93%) as a white foam. R_f = 0.4 (silica gel, CH_2Cl_2 / CH_3OH = 15:1). **$[\alpha]_D^{20}$** = -72.4 (*c* 0.1, $CHCl_3$); **^{1}H NMR** (400 MHz, $CDCl_3$) δ 8.17 (d, *J* = 8.1 Hz, 1H), 8.97 – 8.11 (m, 6H), 7.82 (brs, 1H), 7.72 (d, *J* = 7.4 Hz, 2H), 7.68 – 7.55 (m, 5H), 7.48 (brs, 7H), 7.35 (t, *J* = 7.2 Hz, 2H), 7.29 – 7.22 (m, 2H), 6.29 (brs, 1H), 6.13 (d, *J* = 9.0 Hz, 1H), 5.81 (d, *J* = 8.0 Hz, 1H), 4.67 – 4.51 (m, 3H), 4.44 (brs, 3H), 4.30 (brs, 2H), 4.25 – 4.11 (m, 3H), 4.08– 3.94 (m, 2H), 3.92 – 3.70 (m, 6H), 3.65 (s, 4H), 3.27 (s, 3H), 3.21 (s, 3H), 3.02 (s, 3H), 2.16 – 1.57 (m, 21H), 1.24 (s, 3H), 1.21 (s, 3H). **^{13}C NMR** (100 MHz, $CDCl_3$) δ 174.3, 173.5, 171.9, 170.8, 170.5, 164.9, 164.6, 162.8, 157.3, 151.8, 143.8, 143.7, 141.3, 141.3, 139.0, 134.9, 130.1, 129.1, 127.8, 127.2, 127.2, 126.4, 126.2, 125.2, 125.1, 120.1, 102.8, 100.4, 100.4, 72.4, 69.5, 68.6, 67.4, 62.5, 57.1, 56.4, 55.8, 55.0, 54.8, 53.5, 52.6, 48.1, 47.7, 47.1, 46.9, 29.7, 28.8, 28.1, 27.9, 24.5, 24.2, 23.9, 20.4, 20.3, 17.7, 17.7. **HRMS** (ESI): calcd. for $C_{79}H_{92}N_{10}NaO_{25}S^+$ $[M+Na]^+$ 1635.5848, found 1635.5857.

Synthesis of compound 25b.

25b was synthesized according to the procedures for the synthesis of **25a** from **3b** and **2**.

Analytical data for **25b**: (Yield = 91%), white foam. R_f = 0.4 (silica gel, CH_2Cl_2 / CH_3OH = 15:1). **$[\alpha]_D^{20}$** = -73.8 (*c* 0.2, $CHCl_3$). **Mixture of rotamers.** **^{1}H NMR** (400 MHz, $CDCl_3$) δ 8.06 (t, *J* = 7.9 Hz, 6H), 7.95 (d, *J* = 8.3 Hz, 1H), 7.80 (br s, 1H), 7.73 (d, *J* = 7.4 Hz, 1H), 7.69 – 7.57 (m, 5H), 7.54 – 7.44 (m, 6H), 7.42 (brs, 2H), 7.37 (t, *J* = 7.4 Hz, 2H), 7.31 – 7.27 (m, 1H), 7.21 and 7.13 (d, *J* = 8.3 Hz, 2H), 6.90 – 6.75 (m, 2H), 6.71 and 6.62 (d, *J* = 8.3 Hz, 1H), 6.24 (brs, 1H), 6.11 (d, *J* = 9.1 Hz, 1H), 5.49 (t, *J* = 5.4 Hz, 1H), 5.05 and 4.97 (s, 1H), 4.69 – 4.51 (m, 3H), 4.51 – 4.39 (m, 3H), 4.39 – 4.26 (m, 4H), 4.25 – 4.15 (m, 3H), 4.09 – 3.92 (m, 2H), 3.91 – 3.72 (m, 9H), 3.72 – 3.59 (m, 4H), 3.35 and 3.30 (s, 3H), 3.22 (s, 3H), 3.05 and 3.00 (s, 3H), 2.11 – 1.65 (m, 21H), 1.25 (s, 3H), 1.22 (s, 3H). **^{13}C NMR** (100 MHz, $CDCl_3$) δ 174.4, 173.6, 171.8, 170.8, 170.7, 165.0, 164.7, 162.8, 159.0, 157.4, 156.3, 151.3, 143.9, 143.8, 141.4, 141.4, 135.8, 134.9, 131.1, 130.2, 129.1, 127.9, 127.3, 127.2, 126.6, 126.5, 126.4, 125.3, 125.2, 120.1, 114.1, 100.4, 99.0, 72.1, 69.7, 68.7, 67.5, 62.6, 57.2, 56.6, 55.8 55.4, 55.2, 55.1, 52.7, 48.2, 47.9, 47.1, 44.2, 30.1, 29.8, 28.9, 27.8, 27.7, 24.7, 24.3, 24.0, 20.5, 20.4, 17.8, 17.7. **HRMS** (ESI): calcd. for $C_{88}H_{102}N_{12}NaO_{26}S^+$ $[M+Na]^+$ 1797.6641, found 1797.6646.

Synthesis of compound 25c.

25c was synthesized according to the procedures for the synthesis of **25a** from **3c** and **2**.

Analytical data for **25c**: (Yield = 90%), white foam. R_f = 0.4 (silica gel, CH_2Cl_2 / CH_3OH = 15:1); **$[\alpha]_D^{20}$** = -39.4 (*c* 0.1, $CHCl_3$); **^{1}H NMR** (400 MHz, $CDCl_3$) δ 8.04 (q, *J* = 7.7 Hz, 7H), 7.77 (d, *J* = 7.4 Hz, 2H), 7.72 (d, *J* = 7.6 Hz, 2H), 7.62 (d, *J* = 7.1 Hz, 7H), 7.52 – 7.43 (m, 7H), 7.42 – 7.33 (m, 4H), 7.32 – 7.26 (m, 3H), 6.22 (brs, 1H), 6.18 – 6.06 (m, 2H), 4.67 – 4.53 (m, 3H), 4.53 – 4.36 (m, 5H), 4.35 – 4.25 (m, 3H), 4.25 – 4.13 (m, 3H), 4.10 – 3.93 (m, 2H), 3.87 – 3.73 (m, 5H), 3.72 – 3.61 (m, 4H), 3.41 (s, 3H), 3.23 (s, 3H), 3.05 (s, 3H), 2.12 – 1.61 (m, 21H), 1.26 (s, 3H), 1.23 (s, 3H). **^{13}C NMR** (100 MHz, $CDCl_3$) δ 174.4, 173.5, 171.9, 170.9, 170.5, 164.9, 164.7, 161.8, 158.0, 157.4, 150.9, 144.1, 144.0, 143.8, 143.7, 141.5, 141.4, 141.4, 141.4, 137.1, 134.9, 134.8, 130.2, 129.1, 127.9, 127.8, 127.2, 127.2, 127.2, 126.5, 126.4, 126.3, 125.3, 125.2, 120.1, 100.5, 100.4, 98.6, 72.5, 69.6, 68.6, 67.6, 67.5, 62.5, 57.2, 56.4, 55.9, 55.8, 55.2, 54.9, 52.7, 48.2, 47.9, 47.2, 47.1, 30.6, 29.8, 28.8, 27.8, 27.7, 24.6, 24.2, 23.9, 20.4, 20.4, 17.8, 17.7. **HRMS** (ESI): calcd. for $C_{94}H_{103}N_{11}NaO_{26}S^+$ $[M+Na]^+$ 1856.6689, found 1856.6677.

Synthesis of compound S2.

(±)-Camphorsulfonic acid (158 mg, 0.68 mmol, 0.1 equiv) was added to a solution of **16** (1.22 g, 6.85 mmol, 1.0 equiv), 2,3-butanedione (0.72 mL, 8.22 mmol, 1.2 equiv) and trimethyl orthoformate (3.00 mL, 27.4 mmol, 4.0 equiv) in dry methanol (20 mL). The mixture was heated under reflux for 24 h. The reaction was neutralised with triethylamine (0.5 mL) and the solvents removed under reduced pressure. The residue was purified by silica gel flash chromatography to give the diacetal **S2** (1.72 g, 86%) as a white solid. R_f = 0.4 (silica gel, petroleum ether / EtOAc = 10:1); **m.p.** 130–131 ℃; **$[\alpha]_D^{20}$** = -11.9 (*c* 0.3, $CHCl_3$); **^{1}H NMR** (400 MHz, $CDCl_3$) δ 4.75 (td, *J* = 10.4, 7.2 Hz, 1H), 4.03 (dd, *J* = 10.2, 7.6 Hz, 1H), 3.79 (d, *J* = 7.6 Hz, 1H), 3.74 (s, 3H), 3.28 (s, 3H), 3.26 (s, 3H), 2.95 (dd, *J* = 8.9, 7.3 Hz, 1H), 2.79 (dd, *J* = 10.5, 9.2 Hz, 1H), 1.30 (s, 3H), 1.28 (s, 3H). **^{13}C NMR** (100 MHz, $CDCl_3$) δ 172.0, 100.4, 100.0, 73.4, 68.6, 52.7, 48.3, 48.2, 42.8, 27.7, 18.0, 17.8. **HRMS** (ESI): calcd. for $C_{12}H_{20}NaO_6S^+$ $[M+Na]^+$ 315.0873, found 315.0867.

Synthesis of compound S3.

A mixture of **19** (317 mg, 0.65 mmol, 1.0 equiv), Et_3N (180 μL, 1.30 mmol, 2.0 equiv), DMAP (159 mg, 1.30 mmol, 2.0 equiv), and 2,4,6-triisopropylbenzenesulfonyl chloride (394 mg, 1.30 mmol, 2.0 equiv) in CH_3CN (5 mL) was stirred at room temperature for 1 h. The mixture was cooled in an ice-bath. Concentrated NH_4OH (2 mL) was added, and the mixture was stirred at room temperature for 20 h. The mixture was evaporated, and the residue was partitioned between EtOAc and H_2O. The organic layer was washed with saturated aqueous $NaHCO_3$ and brine, dried over Na_2SO_4, and concentrated *in vacuo*. The residue was purified by silica gel flash chromatography to give **S3** (285 mg, 90%) as a colorless oil. R_f = 0.3 (silica gel, CH_2Cl_2 / CH_3OH = 15:1); **$[\alpha]_D^{20}$** = -93.6 (*c* 0.1, $CHCl_3$); **^{1}H NMR** (400 MHz, $CDCl_3$) δ 8.16 (d, *J* = 7.5 Hz, 1H), 6.31 (d, *J* = 9.1 Hz, 1H),

5.86 (d, *J* = 7.4 Hz, 1H), 4.43 (t, *J* = 9.7 Hz, 1H), 4.18 (dd, *J* = 10.2, 7.8 Hz, 1H), 4.09 (dd, *J* = 10.8, 3.1 Hz, 1H), 3.79 (dd, *J* = 10.8, 2.9 Hz, 1H), 3.38 – 3.32 (m, 1H), 3.20 (s, 3H), 3.04 (s, 3H), 1.26 (s, 3H), 1.24 (s , 3H), 0.96 (s, 9H), 0.16 (s, 3H), 0.14 (s, 3H). **^{13}C NMR** (100 MHz, $CDCl_3$) δ 164.8, 142.6, 100.5, 100.4, 95.9, 73.9, 69.9, 62.9, 57.2, 48.0, 47.9, 46.5, 26.2, 18.9, 17.9, 17.7, -5.0, -5.4. **HRMS** (ESI): calcd. for $C_{21}H_{37}N_3NaO_6SSi^+$ $[M+Na]^+$ 510.2064, found 510.2066.

Supplementary Reference

1. Benz, G. *et al*. Constitution of the deferriform of the albomycins δ_1, δ_2 and ε. *Angew. Chem. Suppl.* 1322−1335 (1982).
