# Supplementary material for: Total synthesis and antimicrobial evaluation of natural albomycins against clinical pathogens
Source: Nat Commun. 2018 Sep 4;9:3445. doi: 10.1038/s41467-018-05821-1 (PMC6123416; doi:10.1038/s41467-018-05821-1)
Supplement: Supplementary file 2 — Peer Revew File [file 41467_2018_5821_MOESM2_ESM.pdf]

Reviewers' comments:

Reviewer #1 (Remarks to the Author):

The authors present the total synthesis on an important antibiotic that might play a pivotal role in anti-infectives research. The paper is basically concerned with the total synthesis of this natural product. Its features are the combination of different natural products classes as a nucleotides and a peptide are combined. The point worth mentioning is the fact that the nucleoside consists of a thiotetrahydrofurane. The use of lyxose as the starting material is a smart choice and in general the construction of the nucleosidic part is creative and elegant. A very nice piece of synthesis! Based on the importance of the molecule and the very well designed synthesis I recommend publication.

Reviewer #2 (Remarks to the Author):

The manuscript by Lin et al. describes the total synthesis of a class of natural products in which some members have antibacterial activity, the albomycins. The paper provides solid synthetic chemical steps in good yields and has identified a platform that can not only synthesize these natural products but could be used to prepare novel analogs that could have improved properties. While the chemistry is well presented, the biological evaluation of these analogs is relatively minimal, and the microbiological testing sections need considerable attention. Given the potential importance of these new analogs at identifying a novel class of clinically useful molecules, more substantial biological testing is warranted. Other minor points are below or in the attached PDF document.

The abstract claims that albomycin ε (1c) is a potent natural product, although the data presented demonstrate that it is inactive, and its activity is stated in the text as being previously unknown. This contrasts with the claim made in the abstract and needs to be reworded with more clarity.
The claim that siderophore uptake compounds are more potent (lower MICs) than those drugs entering through diffusion is not a universal fact – some classes entering through diffusion are very potent. This sentence needs to be clarified differently or removed.
While the 1955 manuscript cited (#10) does indeed state that the MTD for albomycin could not be obtained, the statement should be framed as "…up to the doses tested". Further, since science has evolved significantly over the last 6 decades, these researchers are in a position to update the MTD from their synthetically made molecule and should be encouraged to do so.
What was the yield of the formation of the correct epimer of tripeptide 4 from intermediate 11 prior to the condensation step?
MIC values are typically provided as μg/mL or mg/L units rather than μM and should be changed in the Table and text to the standard format.
The MIC data for the standard ATCC25922 E. coli isolate should be included or else the genotype of strain BJ5183 needs to be referenced. In addition, the data for the S. aureus control strain ATCC29213, also as a representative of a methicillin-sensitive isolate, should be included.
The MIC value for the inactive compound/isolate combinations should be presented as >X, where X is the highest concentration tested rather than NA.
Figure 6 – what is the definition of "susceptible" and "resistant" in this figure? Microbiological data such as this is typically represented in a tabular format where the population statistics of MIC50, MIC90 and MIC range are calculated for the drug of interest and the comparators – and if required, the distribution of isolates is provided as the number of isolates that had a particular MIC value across the range tested. In all cases, the MIC value should be in μg/mL or mg/L units rather than μM. This enables the reader to interpret the relative resistance levels of the population using the interpretive criteria or breakpoints of CLSI or EUCAST.
There are several inconsistencies with respect to the listed MIC method and the standardized

microdilution protocol that could impact the validity of the microbiological data. Firstly, the CLSI guidelines for broth microdilution assays suggests a final concentration of ~5x105 CFU/mL being used in the assay plates (typically ~100 µL or 5x104 CFU are used per well of a 96=well plate). The methods section suggests that a lower inoculum was used – is this the case? Secondly, the growth media is listed as Mueller-Hinton broth, whereas the standardized protocol states that cation-adjusted Mueller-Hinton broth (CA-MHB) be used for the species (except N. gonorrheae) tested here. Lastly, did the reading of the MIC values at 18-24h alter with the species being tested, as S. aureus and the Enterobacteriaceae are 16-20h whereas S. pneumoniae and N. gonorrhoeae are 20-24 hours.
What is the mechanism of resistant to albomycin in the two S. pneumoniae isolates (S15 and 29 in supplemental table)? As the clinical breakpoint has not been determined for albomycin, testing at higher concentrations should be performed with the hope of obtaining a susceptibility endpoint for these isolates and a rationale for their mechanism of resistance, possibly a mutation in the seryl-tRNA-synthetase gene, would help understand the potential value of these agents. A subsequent bioinformatic analysis across the public S. pneumoniae genome sequences would help understand what the prevalence of resistance could be – as 2/17 random isolates = >10% which would significantly hinder clinical development and is suggestive of a pre-existing resistance mechanism in the population.

The chemistry is provided in sufficient detail that it should be able to be reproduced, and the microbiological testing is standard and could be reproduced (although inclusion of a standard E. coli isolate as suggested is warranted).

Reviewer #3 (Remarks to the Author):

Summary:

He and coworkers report the first total syntheses of albomycins δ1, δ2, and ε in a convergent manner with high yielding steps. Structure-activity relationships in these albomycins were identified through evaluation of the MIC data in both Gram-negative and Gram-positive strains. This revealed the importance of the nucleobase substitution on their antimicrobial activity and their selectivity in Gram-negative and Gram-positive strains. They demonstrate that albomycin δ2 exhibited superior antimicrobial activities (MIC data) against 27 clinical isolates of Streptococcus pneumoniae and Staphylococcus aureus (including MRSA strains) relative to vancomycin, penicillin G, and ciprofloxacin.

Rating:

Significance: High
Novelty: High
Broad Interest: High
Scholarly Presentation: Moderate

Comments:

The first total syntheses of the albomycins presented in this work are highly significant because these molecules are promising as antibiotic candidates, yet their isolation procedure from Streptomyces griseus is tedious and non-scalable. Although the synthetic route to these molecules has a high step count, each individual step is high yielding and their overall strategy is convergent and would allow for the synthesis of analogs for further investigation. The authors established the importance of the C4 nucleobase on albomycin antimicrobial activity and species selectivity. It is significant that the MIC values of albomycin δ2 against 27 clinical isolates of S. pneumonia and S. aureus (including MRSA strains) were largely superior to ciprofloxacin, vancomycin, and penicillin G.

Critiques:

There are many grammatical errors throughout the manuscript that should be addressed through a careful proofread. One repeated error is the absence of an “s” on the end of words, often due to incorrect verb conjugation. There are two such errors in the first two sentences of the abstract, for example.

Albomycins have been successfully used in humans (see Brit. Med. J. 1955, 1177-1179), and the authors should definitely point this out in the beginning of their manuscript.

During the discussion of the synthesis, the authors have a tendency to talk about failed reactions not present in the schemes before discussing the successful reaction in the scheme. This happens several times in the paper, and can be very confusing to the reader. One standout example is the discussion of the transformation of 11 to 4, in which the authors refer to “Miller’s protocol” but do not define this beyond a citation. The authors should feel free to discuss failed reactions (many readers will be interested in them), but care should be taken to achieve clarity, perhaps by including some failed reactions within the schemes.

The authors should comment in the manuscript about iron content in their MIC studies. Mueller Hinton broth contains free iron, which can lead to inconsistencies between in vitro and in vivo data in siderophore-containing antibiotics, especially for Gram-negative bacteria (see Antimicrob. Agents Chemother. 2016, 60, 7396-7401). It is possible that if the authors use iron-depleted MH broth for MIC microdilution studies, the activities of albomycins might be significantly enhanced. At the very minimum, the authors should indicate whether iron-depleted media was used.

Minor Critiques:

- WHO should be defined when it is first used.
- “Log” or "log10" should be used in the y-axis label Fig. 6 instead of “lg,” which has varying international definitions.
- In the general methods section, “residual protium” should be used instead of “residual solvent” when referring to NMR internal standards.
- The key for Fig. 6 should be revised: “susceptible” and “resistant” are ambiguous terms. MSSA/MRSA could be used for S. aureus, and DSSP/DRSP is standard for S. pneumoniae (unfortunately, the latter terms are also ambiguous, but they are commonly used).

Conclusion:

This reviewer recommends this manuscript for publication in Nature Communications.

Reviewer #1 (Remarks to the Author):

The authors present the total synthesis on an important antibiotic that might play a pivotal role in anti-infectives research. The paper is basically concerned with the total synthesis of this natural product. Its features are the combination of different natural products classes as a nucleotides and a peptide are combined. The point worth mentioning is the fact that the nucleoside consists of a thiotetrahydrofurane. The use of lyxose as the starting material is a smart choice and in general the construction of the nucleosidic part is creative and elegant. A very nice piece of synthesis! Based on the importance of the molecule and the very well designed synthesis I recommend publication.

Reviewer #2 (Remarks to the Author):

The manuscript by Lin et al. describes the total synthesis of a class of natural products in which some members have antibacterial activity, the albomycins. The paper provides solid synthetic chemical steps in good yields and has identified a platform that can not only synthesize these natural products but could be used to prepare novel analogs that could have improved properties. While the chemistry is well presented, the biological evaluation of these analogs is relatively minimal, and the microbiological testing sections need considerable attention. Given the potential importance of these new analogs at identifying a novel class of clinically useful molecules, more substantial biological testing is warranted. Other minor points are below or in the attached PDF document.

1. The abstract claims that albomycin ε (**1c**) is a potent natural product, although the data presented demonstrate that it is inactive, and its activity is stated in the text as being previously unknown. This contrasts with the claim made in the abstract and needs to be reworded with more clarity.

Response: The phrase "we report the first total synthesis of the extremely potent natural antibiotics, albomycins δ_1 (**1a**), δ_2 (**1b**) and ε (**1c**)" has been reworded to "we report the first total synthesis of natural albomycins δ_1 (**1a**), δ_2 (**1b**) and ε (**1c**)".

2. The claim that siderophore uptake compounds are more potent (lower MICs) than those drugs entering through diffusion is not a universal fact – some classes entering through diffusion are very potent. This sentence needs to be clarified differently or removed.

Response: The description "which are much lower than those of antibiotics entering cells through diffusion" has been removed.

3. While the 1955 manuscript cited (#10) does indeed state that the MTD for albomycin could not be obtained, the statement should be framed as "…up to the doses tested". Further, since science has evolved significantly over the last 6 decades, these researchers are in a position to update the MTD from their synthetically made molecule and should be encouraged to do so.

Response: The phrase "even it was too safe to determine its lethal dose for experimental animals" has been revised to "it was well tolerated and safe up to a maximum dose evaluated in mice[10]".

4. What was the yield of the formation of the correct epimer of tripeptide **4** from intermediate **11** prior to the condensation step?

Response: The yield was 77% over two steps, and it has been added in the revised manuscript.

5. MIC values are typically provided as µg/mL or mg/L units rather than µM and should be changed in the Table and text to the standard format.

Response: MIC values have been changed to µg/mL now.

6. The MIC data for the standard ATCC25922 *E. coli* isolate should be included or else the genotype of strain BJ5183 needs to be referenced. In addition, the data for the *S. aureus* control strain ATCC29213, also as a representative of a methicillin-sensitive isolate, should be included.

Response: The MIC data for the standard strains such as *E. coli* ATCC 25922, *S. pneumoniae* ATCC 49619 and *S. aureus* ATCC 29213 have been included in Table 3. A reference (ref. 42) have been cited for the genotype of *E. coli* BJ 5183 in the revised manuscript.

7. The MIC value for the inactive compound/isolate combinations should be presented as >X, where X is the highest concentration tested rather than NA.

Response: "NA" has been replaced with ">512".

8. Figure 6 – what is the definition of "susceptible" and "resistant" in this figure? Microbiological data such as this is typically represented in a tabular format where the population statistics of MIC50, MIC90 and MIC range are calculated for the drug of interest and the comparators – and if required, the distribution of isolates is provided as the number of isolates that had a particular MIC value across the range tested. In all cases, the MIC value should be in µg/mL or mg/L units rather than µM. This enables the reader to interpret the relative resistance levels of the population using the interpretive criteria or breakpoints of CLSI or EUCAST.

Response: Figure 6 has been replaced with Table 2. Words "susceptible" and "resistant" are no longer used, and potential confusions are avoided. The microbiological data are now presented in tabular format (Table 2) as suggested. The MIC values are in µg/mL now.

9. There are several inconsistencies with respect to the listed MIC method and the standardized microdilution protocol that could impact the validity of the microbiological data. Firstly, the CLSI guidelines for broth microdilution assays suggests a final concentration of ~$5x10^5$ CFU/mL being used in the assay plates (typically ~100 µL or $5x10^4$ CFU are used per well of a 96=well plate). The methods section suggests that a lower inoculum was used – is this the case? Secondly, the growth media is listed as Mueller-Hinton broth, whereas the standardized protocol states that cation-adjusted Mueller-Hinton broth (CA-MHB) be used for the species (except *N. gonorrhoeae*) tested here. Lastly, did the reading of the MIC values at 18-24 h alter with the species being tested, as *S. aureus* and the Enterobacteriaceae are 16-20 h whereas *S. pneumoniae* and *N. gonorrhoeae* are 20-24 hours.

Response: MIC values were determined according to the recommendations of the Clinical and Laboratory Standard Institute (CLSIM07). Except for *N. gonorrhoeae*, all strains were tested by broth microdilution assays at a final concentration of $5x10^5$ CFU/mL. We have revised the info in the text.

For *N. gonorrhoeae*, MIC values were determined by the standard agar dilution method on GC medium base with Isovitalex (Topbio, Shandong, China) at 37 °C with 5% CO_2; for *S. pneumoniae*, MIC values were tested using 96-well microdilution plates in Mueller-Hinton broth II with 5% horse-blood (Solarbio, Beijing, China) at 37 °C with 5% CO_2; other strains were detected by the standard 96-well microdilution method in Mueller-Hinton broth II (Solarbio, Beijing, China) at 37 °C.

The CLSIM07 states that cation-adjusted Mueller-Hinton broth (CAMHB) is used for these species. The Mueller-Hinton broth II is cation-adjusted Mueller-Hinton broth (CAMHB).

For *S. aureus*, the MIC values were read at 18-20 h; For Enterobacteriaceae, it was about 20 h; For *S. pneumoniae* and *N. gonorrhoeae*, it was 20-24 h. Therefore, the reading of the MIC values was performed at 18-24 h.

10. What is the mechanism of resistant to albomycin in the two *S. pneumoniae* isolates (S15 and 29 in supplemental table)? As the clinical breakpoint has not been determined for albomycin, testing at higher concentrations should be performed with the hope of obtaining a susceptibility endpoint for these isolates and a rationale for their mechanism of resistance, possibly a mutation in the seryl-tRNA-synthetase gene, would help understand the potential value of these agents. A subsequent bioinformatic analysis across the public *S. pneumoniae* genome sequences would help understand what the prevalence of resistance could be – as 2/17 random isolates = >10% which would significantly hinder clinical development and is suggestive of a pre-existing resistance mechanism in the population.

Response: A reported albomycin resistance mechanism of some pathogens rapidly grown on rich media agar plates was mutations in an uptake transporter gene like TonA. No mutated serRS gene that is resistant to albomycin, has been evolved from an initially susceptible serRS. The transporter mutation likely most often occurs in laboratory based *in-vitro* experiments. Both transporter and serRS mutations should bear a fitness cost to mutants in animal models or humans.

Higher concentrations of albomycin δ_2 have been tested for *S. pneumoniae* isolates S15 and 29, the MIC values were 8 μg/mL and 16 μg/mL, respectively. However, in iron-deficient Mueller-Hinton II broth media, the antibiotic activity was significantly increased, and the MIC values were 0.125 μg/mL and 0.5 μg/mL respectively. These data indicate that a pre-existing resistance mechanism caused by the aforementioned genetic variations in the population may not occur.

The chemistry is provided in sufficient detail that it should be able to be reproduced, and the microbiological testing is standard and could be reproduced (although inclusion of a standard *E. coli* isolate as suggested is warranted).

Reviewer #3 (Remarks to the Author):

Summary:

He and coworkers report the first total syntheses of albomycins δ_1, δ_2, and ε in a convergent manner with high yielding steps. Structure-activity relationships in these albomycins were

identified through evaluation of the MIC data in both Gram-negative and Gram-positive strains. This revealed the importance of the nucleobase substitution on their antimicrobial activity and their selectivity in Gram-negative and Gram-positive strains. They demonstrate that albomycin δ_2 exhibited superior antimicrobial activities (MIC data) against 27 clinical isolates of *Streptococcus pneumoniae* and *Staphylococcus aureus* (including MRSA strains) relative to vancomycin, penicillin G, and ciprofloxacin.

Rating:

Significance: High
Novelty: High
Broad Interest: High
Scholarly Presentation: Moderate

Comments:

The first total syntheses of the albomycins presented in this work are highly significant because these molecules are promising as antibiotic candidates, yet their isolation procedure from *Streptomyces griseus* is tedious and non-scalable. Although the synthetic route to these molecules has a high step count, each individual step is high yielding and their overall strategy is convergent and would allow for the synthesis of analogs for further investigation. The authors established the importance of the C4 nucleobase on albomycin antimicrobial activity and species selectivity. It is significant that the MIC values of albomycin δ_2 against 27 clinical isolates of *S. pneumonia* and *S. aureus* (including MRSA strains) were largely superior to ciprofloxacin, vancomycin, and penicillin G.

Critiques:

1. There are many grammatical errors throughout the manuscript that should be addressed through a careful proofread. One repeated error is the absence of an “s” on the end of words, often due to incorrect verb conjugation. There are two such errors in the first two sentences of the abstract, for example.

Response: We have addressed grammatical errors in the revised manuscript.

2. Albomycins have been successfully used in humans (see Brit. Med. J. 1955, 1177-1179), and the authors should definitely point this out in the beginning of their manuscript.

Response: The relevant statement about “albomycins have been successfully used in humans” has been added in the revised manuscript.

3. During the discussion of the synthesis, the authors have a tendency to talk about failed reactions not present in the schemes before discussing the successful reaction in the scheme. This happens several times in the paper, and can be very confusing to the reader. One standout example is the discussion of the transformation of 11 to 4, in which the authors refer to “Miller’s protocol” but do not define this beyond a citation. The authors should feel free to discuss failed reactions (many

readers will be interested in them), but care should be taken to achieve clarity, perhaps by including some failed reactions within the schemes.

Response: a) The “Miller’s protocol” has been replaced with “under active ester-mediated coupling conditions (DCC, NHS)[20]”, which could be much clearer to the reader. b) The “Huge efforts were made to selectively protect the 2'-OH of **16** as an ester, but no satisfactory result was obtained” was deleted to avoid possible confusion. c) The “commonly used aminolysis of *N*4-phenoxycarbonyl did not take place due to the steric hindrance of the N3 methyl group” has been revised to “aminolysis of *N*4-phenoxycarbonyl[37] did not take place due to the steric hindrance of the N3 methyl group”. A citation has been given here.

4. The authors should comment in the manuscript about iron content in their MIC studies. Mueller Hinton broth contains free iron, which can lead to inconsistencies between *in vitro* and *in vivo* data in siderophore-containing antibiotics, especially for Gram-negative bacteria (see Antimicrob. Agents Chemother. 2016, 60, 7396-7401). It is possible that if the authors use iron-depleted MH broth for MIC microdilution studies, the activities of albomycins might be significantly enhanced. At the very minimum, the authors should indicate whether iron-depleted media was used.

Response: Iron-depleted media has been used, and the data (Table 3) and comments have been included in the revised manuscript.

Minor Critiques:

5. WHO should be defined when it is first used.

Response: WHO has been defined in the revised manuscript.

6. “Log” or "log10" should be used in the y-axis label Fig. 6 instead of “lg,” which has varying international definitions.

Response: We have deleted Fig. 6, so this potential confusion is avoided.

7. In the general methods section, “residual protium” should be used instead of “residual solvent” when referring to NMR internal standards.

Response: The “residual solvent” has been replaced with “residual protium”.

8. The key for Fig. 6 should be revised: “susceptible” and “resistant” are ambiguous terms. MSSA/MRSA could be used for S. aureus, and DSSP/DRSP is standard for *S. pneumoniae* (unfortunately, the latter terms are also ambiguous, but they are commonly used).

Response: We have deleted Fig. 6, so this potential confusion is avoided.

Conclusion:

This reviewer recommends this manuscript for publication in *Nature Communications*.

REVIEWERS' COMMENTS:

Reviewer #3 (Remarks to the Author):

The authors did a very comprehensive job of addressing all of the reviewers' comments. Only a couple of comments on the revised manuscript:

First: the new tables express MIC values in ug/mL, which is standard. However, usually these are not expressed with several significant digits. Since a microdilution assay has a margin of error of 1-2 dilutions, three significant digits is misleading, and implies higher accuracy than the assay is capable of achieving. For example, an MIC value of 0.5 ug/mL should be expressed as 0.5, not 0.500. The same applies to all similar values. Common values: 64, 32, 16, 8, 4, 2, 1, 0.5, 0.25, 0.125, 0.0625 etc.

The inserted sentences about iron content in the media are somewhat confusing. I suggest the following revised language, or similar:

The influence of iron concentration on the antibacterial activity was also studied by conducting assays in iron-rich and iron-deficient media (Table 3). The antibiotic activity of albomycin δ2 against S. pneumoniae strains was significantly increased in iron-deficient media, which most closely mimics the physiological situation in a human host, wherein iron is sequestered in macromolecules such as heme.(INSERT REFERENCE) Two isolates, strains S15 and 29, which showed the most resistance to albomycin δ2 in iron-rich media, became highly susceptible under iron-depleted conditions. The MIC values of albomycin δ2 against S. aureus and E. coli strains were not influenced by iron concentration. The three control antibiotics did not show any dependence on iron concentration in any of the strains tested.

After these minor corrections, this reviewer recommends publication in Nature Communications.

Dear Referees:

On behalf of my co-authors, we thank you very much for your very helpful comments and suggestions concerning our manuscript entitled “Total Synthesis and Antimicrobial Evaluation of Natural Albomycins against Clinical Pathogens”. Those comments are very valuable and very helpful for revising our manuscript. We have made revisions accordingly, which we hope meets with approval.

Thank you and best regards.

Reviewer #3 (Remarks to the Author):

The authors did a very comprehensive job of addressing all of the reviewers' comments. Only a couple of comments on the revised manuscript:

1. First: the new tables express MIC values in ug/mL, which is standard. However, usually these are not expressed with several significant digits. Since a microdilution assay has a margin of error of 1-2 dilutions, three significant digits is misleading, and implies higher accuracy than the assay is capable of achieving. For example, an MIC value of 0.5 ug/mL should be expressed as 0.5, not 0.500. The same applies to all similar values. Common values: 64, 32, 16, 8, 4, 2, 1, 0.5, 0.25, 0.125, 0.0625 etc.

Response: MIC values have been expressed with common values instead of four significant digits.

2. The inserted sentences about iron content in the media are somewhat confusing. I suggest the following revised language, or similar:

The influence of iron concentration on the antibacterial activity was also studied by conducting assays in iron-rich and iron-deficient media (Table 3). The antibiotic activity of albomycin δ_2 against *S. pneumoniae* strains was significantly increased in iron-deficient media, which most closely mimics the physiological situation in a human host, wherein iron is sequestered in macromolecules such as heme. (INSERT REFERENCE) Two isolates, strains S15 and 29, which showed the most resistance to albomycin δ_2 in iron-rich media, became highly susceptible under iron-depleted conditions. The MIC values of albomycin δ_2 against *S. aureus* and *E. coli* strains were not influenced by iron concentration. The three control antibiotics did not show any dependence on iron concentration in any of the strains tested.

Response: The inserted sentences about iron content in the media have been replaced with the above language suggested by the reviewer and reference 43 has been inserted.

Reference 43: Ito, A. *et al.* Siderophore cephalosporin cefiderocol utilizes ferric iron transporter systems for antibacterial activity against *Pseudomonas aeruginosa*. *Antimicrob. Agents Chemother,* **60,** 7396−7401 (2016).

After these minor corrections, this reviewer recommends publication in Nature Communications.
